# A novel and ubiquitous miRNA-involved regulatory module ensures precise phosphorylation of RNA polymerase II and proper transcription

**DOI:** 10.1371/journal.ppat.1012138

**Published:** 2024-04-19

**Authors:** Zhiwen Wang, Shan Zhong, Sicong Zhang, Borui Zhang, Yang Zheng, Ye Sun, Qinghua Zhang, Xili Liu

**Affiliations:** 1 China Agricultural University, Beijing, China; 2 Sanya Institute of China Agricultural University, Sanya, China; 3 State Key Laboratory or Crop Stress Resistance and High-Efficiency Production, Northwest A&F University, Yangling, China; Purdue University, UNITED STATES

## Abstract

Proper transcription orchestrated by RNA polymerase II (RNPII) is crucial for cellular development, which is rely on the phosphorylation state of RNPII’s carboxyl-terminal domain (CTD). Sporangia, developed from mycelia, are essential for the destructive oomycetes *Phytophthora*, remarkable transcriptional changes are observed during the morphological transition. However, how these changes are rapidly triggered and their relationship with the versatile RNPII-CTD phosphorylation remain enigmatic. Herein, we found that *Phytophthora capsici* undergone an elevation of Ser5-phosphorylation in its uncanonical heptapeptide repeats of RNPII-CTD during sporangia development, which subsequently changed the chromosomal occupation of RNPII and primarily activated transcription of certain genes. A cyclin-dependent kinase, PcCDK7, was highly induced and phosphorylated RNPII-CTD during this morphological transition. Mechanistically, a novel DCL1-dependent microRNA, pcamiR1, was found to be a feedback modulator for the precise phosphorylation of RNPII-CTD by complexing with PcAGO1 and regulating the accumulation of PcCDK7. Moreover, this study revealed that the pcamiR1-CDK7-RNPII regulatory module is evolutionarily conserved and the impairment of the balance between pcamiR1 and *PcCDK7* could efficiently reduce growth and virulence of *P*. *capsici*. Collectively, this study uncovers a novel and evolutionary conserved mechanism of transcription regulation which could facilitate correct development and identifies pcamiR1 as a promising target for disease control.

## Author summary

Ser5 phosphorylation (S5p) on the carboxyl-terminal domain of RNA polymerase II (RNPII-CTD) is essential for RNPII activation and enables it properly transcribes genes. Sporangium is dispensable for the development and infection of *Phytophthora*, it differentiates from hypha rapidly and undergoes remarkable transcriptional changes during the morphological transition. However, the regulatory machinery of the changes and the role of RNPII-CTD-Ser5p in the transition remain a mystery. Herein, a novel microRNA-involved module, pcamiR1-CDK7-RNPII, which could rapidly and precisely ensure transcriptional plasticity by regulating the phosphorylation of an uncanonical heptapeptide repeats of RNPII-CTD is reported. The discoveries could broaden the understanding of transcriptional regulation through a novel way, also could strongly benefit the control of many human and plant diseases based on the evolutionary conserved microRNA with a safe and convenient way.

## Introduction

MicroRNA (miRNA) is an important regulator involved in gene silencing, which is an essential mechanism of post-transcriptional gene regulation for cellular development and stress responses. MiRNAs originate from single-stranded RNA precursors (pre-miRNAs) and are transcribed by RNA polymerase II (RNPII) to produce hairpin primary miRNA transcripts (pri-miRNAs) [1]. Drosha, Dicer or Dicer-like (DCL) proteins, which belong to the endoribonuclease III family in different species, function as microprocessors to generate mature miRNA duplexes from the pri-miRNAs or pre-miRNAs [2]. The mature miRNAs are then loaded into ARGONAUTE proteins (AGOs) to form RNA-induced silencing complexes (RISCs), finally repressing the expression of target genes with sequences complementary to the miRNAs [3]. miRNAs mediate gene silencing by promoting translational inhibition or mRNA degradation [4]. In Animalia, miRNA usually binds to mRNA through its 2–8 nt seed sequence, inducing target degradation by accelerating deadenylation or translational repression through the inhibition of ribosome assembly and translation in microsomal/membrane-bound polysomes, respectively [5]. Meanwhile, miRNA could perfectly bind to mRNA to form miRNA-mRNA duplexes and initiate target degradation through endonucleolytic cleavage catalyzed by AGOs in plants [6]. The biogenetic pathway of miRNA in microbes was first identified in the model filamentous fungus *Neurospora crassa* and appears to be more complex and diverse than in animals and plants [7], as reflected by the involvement of many components other than Dicers, such as quelling-deficient 2 (QDE-2) and the exonuclease QIP [8]. Recent studies mainly focused on the function of miRNAs in the infection process of microbes; such miRNAs were found in *Puccinia striiformis* f. sp. *tritici*, *Verticillium dahliae*, *Valsa mali*, and *Fusarium oxysporum* and could repress the expression of fungal virulence genes or plant resistance genes [8–10]. In oomycetes, the only confirmed miRNA miR8788 was identified in *Phytophthora infestans* and was found to target an alpha/beta hydrolase-type encoding gene in host, *StABH1*, whose suppression promotes pathogen growth in potato [11]. Although the key gene-silencing components found in eukaryotic organisms are also present in *Phytophthoras*, such as DCLs and AGOs, some silencing-related proteins such as HUA enhancer 1, RNA polymerase IV, Drosha, and ERI1 have not been identified in their genomes [12], which implies that *Phytophthoras* have an unusual gene-silencing mechanism. Moreover, it is worth exploring whether miRNA could control cell development by modulating endogenous genes in microbes apart from cross-kingdom regulation, especially those responsible for the formation of important development- and pathogenesis-related apparatuses in microorganisms.

Transcription orchestrated by RNPII is one of the most important cellular events, it could be categorized as initiation, elongation, and termination states according to the chromosomal occupation of RNPII [13]. The multi-subunit RNPII is evolutionarily conserved from microbes to mammals. Its largest subunit, Rpb1, contains a carboxyl-terminal domain (CTD) consisting of conserved heptapeptide repeats with the consensus sequence Y_1_S_2_P_3_T_4_S_5_P_6_S_7_ in higher organisms [14]. The number of repeats ranges from 26 in yeast to 52 in mammals, and the repeats are extensively modified post-translationally, principally by the phosphorylation of Ser5 (S5p) and Ser2 (S2p) [15]. In mammals, RNPII CTD is largely unphosphorylated when it is silent but converted to S5p following transcriptional activation; further elongation in the gene correlates with a reduction in S5p but an increase in S2p. Phosphorylation of RNPII is indispensable for proper transcription activation, especially the S5p, which initiates the transcription of certain genes and subsequently triggers the further phosphorylation of RNPII on Ser2, Ser7, Thr4, and Tyr1 (Y1p) [16]. Interestingly, heptapeptide repeats are absent in many lower organisms like fungi and bacteria, but uncanonical heptapeptide with a single nucleotide substitution of alanine instead of serine in the seventh position are existed in *Phytophthora* RNPII-CTD, whether the unique CTD could affect the role of RNPII in transcription remains to be explored.

The phosphorylation of RNPII CTD depends on CTD kinases (often members of cyclin-dependent kinases family [CDKs]) and its dephosphorylation rely on phosphatases; the interplay between them provides a means for coupling and coordinating specific stages of transcription by recruiting various transcription factors or mediators required for proper gene expression [17]. CDKs are a family of serine/threonine protein kinases that interact with cyclins and lie at the heart of eukaryotic cell cycle control [18]. It is important for CDKs to be activated at the proper time and modulated to a precise level during various stages of the cell cycle to ensure normal growth. The Ras superfamily of small GTPases, including Ras and Rho families, act as a signaling switch of phosphorylation state by regulating the expression of CDKs and CTD phosphatases FCP1 or RPAP2 through an indirect effect [19]. However, the direct regulator of CDKs expression remains enigmatic, the rapid and precise regulation is necessary to ensure transcriptional accuracy. Moreover, the dysregulation of transcription mediated by overactive CDKs is associated with the dysfunction of cell cycle regulation, which causes many diseases in humans, including cancer [20]. CDK7 is expected to become an important target for cancer treatment. Some CDK7 inhibitors, such as THZ1, could function as efficient drugs to control cancer cell proliferation or death [19]. As the initial step of RNPII-CTD phosphorylation, S5p modulated by CDK7 is dispensable for proper transcription [21]. However, the interaction between CDK7 and the unique YSPTSPA heptapeptide repeats in *Phytophthora* RNPII is unclear, the exploration of CDK7 repressor could further the development of new anti-tumor drugs.

*Phytophthora* is a genus of oomycetes that resemble true fungi in morphology and pathogenesis, yet is phylogenetically distinct from fungi and could form some specific apparatus like sporangium [22]. This commonly-held misconception lead to a reduced effectiveness by using conventional fungicides to prevent *Phytophthora*, thus it demands the identification of novel and primary targets to precisely control these pathogens [23]. Over a hundred species of *Phytophthora* have been reported, including the notorious plant pathogen *Phytophthora capsici*, which is a destructive plant pathogen that causes root, fruit, and foliar diseases on more than 100 important crops [24]. *Phytophthora* can reproduce sexually to yield oospores and asexually to produce sporangia, with the production of asexual sporangia predominating in nature [25]. Sporangium is the primary infective propagule of *Phytophthora*, in response to moist, cold conditions, mature sporangium cleaves into biflagellate zoospores, which swim, encyst, and germinate to form mycelia or a specialized infection structure (appressorium) on hosts or hydrophobic surfaces [26]. Sporangium can also germinate directly to produce mycelia or form an appressorium [27]. After establishing haustoria in host cells, *Phytophthora* sporulates within 2–4 days of infection under ideal conditions, with the emergence of more sporangia, so that multiple infection cycles can spread the disease rapidly through an entire field; thus, the impairment of sporangia production could destroy the virulence of *Phytophthora* [28]. Apart from environmental factors, the rapid formation of sporangia in *Phytophthora* species (usually < 6 hours after sporangiophore development initiates) is controlled by various genes. RNA-seq of *P*. *infestans* life stages showed that > 22% (> 4,000) genes were upregulated in sporangia compared to hypha, more than one-third of these genes were upregulated > 100-fold [29]. Many genes, including those encoding cell cycle regulators, PiCdc14 [30] and PcSDA1 [31], G protein β and γ subunits [32], Myb and MADS-box transcription factors [33], the loricrin-like protein PiLLP [34], the mating pheromone-induced death 1 protein PpMID1 [35], and the catalase PiCAT2 [36], are involved in sporangia development in *Phytophthora*. However, despite its importance in the *Phytophthora* disease cycle, the primary regulator of the dramatically transcriptional changes during sporangia formation remains poorly understand, the clarification of the regulatory mechanism could strongly benefit the design of oomycetes-specific microbicide to combat with the diseases through inhibiting the development of *Phytophthora*.

As a model *Phytophthora* species, *P*. *capsici* has a broad range of hosts and a feasible gene editing system to perform functional genomics studies [37]. Herein, using *P*. *capsici* as a model, it was demonstrated that RNPII CTD phosphorylation plasticity regulated by the novel microRNA-CDK7 module played a key role in sporangia development in *Phytophthora*. The uncanonical heptapeptide repeats of RNPII CTD in *P*. *capsici* exhibited rapid and precise phosphorylation dynamics during the morphological transition. Moreover, multiple sequencing and genetic analyses revealed that a novel DCL1-dependent microRNA, pcamiR1, acted as a feedback regulator to modulate the phosphorylation state of RNPII through the accumulation of CDK7. Conjoint analysis by chromatin immunoprecipitation sequencing (ChIP-seq) and RNA-seq uncovered that diverse transcriptome profiles were primarily triggered by different phosphorylation states of RNPII through their characteristic chromosomal occupations, which ultimately resulted in the normal/abnormal differentiation from vegetative growth to sporangia in *Phytophthora* and induced the upregulation of pcamiR1 and CDK7 indirectly during this developmental transition. Otherwise, it was confirmed that the pcamiR1-CDK7-RNPII axis was conserved in mammalian cells and pcamiR1 was a promising target for disease control. Therefore, our study illustrated a novel and conserved mechanism of transcriptional regulation via an efficient RNAi pathway.

## Results

### *P*. *capsici* undergone remarkable transcriptional changes and rapid elevation of RNPII CTD phosphorylation during the morphological transition from hypha growth to sporangia development

**Fig 1:**
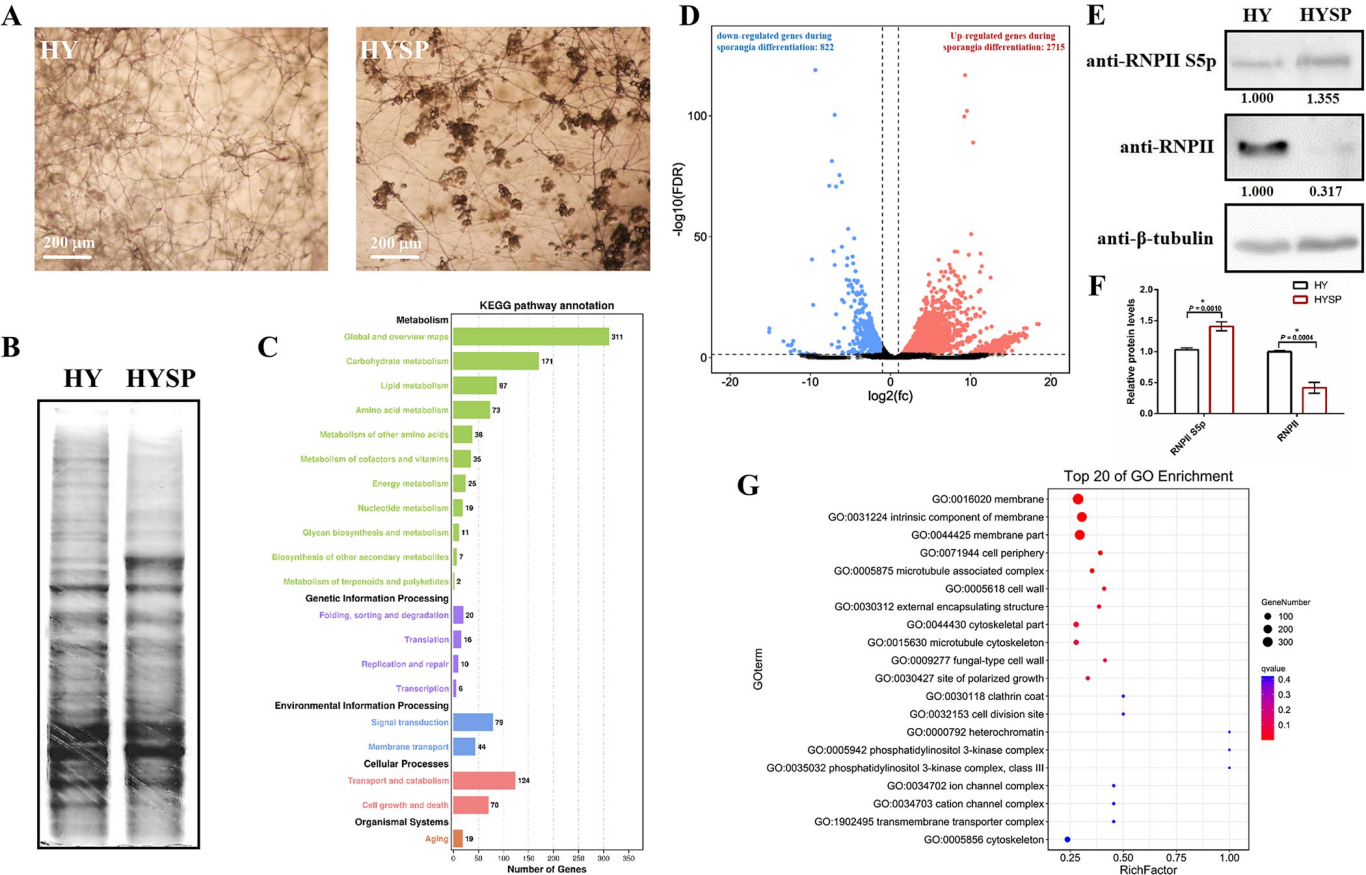
*Phytophthora capsici* undergoes dramatic changes in transcription and RNPII Ser5 phosphorylation during sporangia formation. (A) Images of *P*. *capsici* during sporangia formation. The left panel shows the hypha growth state (HY) of *P*. *capsici*. The image was taken 2 days after *P*. *capsici* was inoculated on V8 medium and incubated at 25°C in the dark. The right panel shows the sporangia formation state (sporangia were produced at the tips of the hyphae; HYSP) of *P*. *capsici*. The image was taken 3 days post inoculation under the same conditions. Scale bar: 200 μm. (B) SDS-PAGE analysis of the proteomic profiles of *P*. *capsici* before (HY) or after (HYSP) sporangia development. (C, D, and G) Transcriptomic changes in *P*. *capsici* during sporangia formation. D shows the gene profile with altered expression levels during *P*. *capsici* sporangia formation, while C and G show the enriched KEGG and GO pathways of the altered genes. (E and F) RNPII undergoes an elevation of Ser5 phosphorylation in the CTD during sporangia formation. The relative intensity of Ser5-phosphorylated RNPII and unphosphorylated RNPII in the hypha stage (HY) and sporulated hypha stage (HYSP) was quantified with ImageJ (E). Data presented in F are as the mean ± SD of three replicates. Statistical significance compared to the HY was determined using Student’s t-test (* *P* < 0.01). β-tubulin was used as the loading control and its intensity in each sample was used to normalize the data between samples.

*P*. *capsici* remains hypha growth state within 48 h in V8 liquid medium at 25°C in the dark. Sporangia formation is a rapid process (within a few hours) and their emergence usually occurs after 60 h, and plentiful sporangia generate between 72 h and 120 h, differing in various isolates. Here, we focused on the transition from hypha growth to sporangia differentiation. Hypha tissues of LT1534 at 48 h (without sporangium) and 72 h (with plenty of sporangia) were collected and analyzed (Fig 1A). SDS-PAGE showed that the protein expression profile was significantly changed during the morphological transition in *P*. *capsici* (Fig 1B). Transcriptomic analysis showed remarkable transcriptional changes during sporangia development, reflected by the 2,715 upregulated genes and 822 downregulated genes in the transition (sporulated hypha /hypha-only, fold change ≥ 2 and false discovery rate [FDR] < 0.05, Figs 1D and A in S1 Text), which account for ~20% of the total number of the genes annotated in *P*. *capsici* genome and this is similar to the transcriptomic change in *P*. *infestans*. Further bioinformatics analysis indicated that the genes differentially expressed in the two stages were mainly involved in membrane composition, cytoskeleton regulation, signal transduction, cellular transportation, and metabolism (Figs 1C, 1G and A in S1 Text).

Transcription is orchestrated by RNPII, no antibody is available to simultaneously detect phosphorylated and unphosphorylated RNPII. RNPII-S5p is the primary step of phosphorylation and activates transcription of certain genes, the phosphorylation on other RNPII-CTD sites follows the activation and is happened at the binding loci of RNPII-S5p, thus S5p is the most representable sign of actively transcribing genes and the phosphorylation state of RNPII. Here it was showed that RNPII S5p was obviously elevated during the transition, while as a comparison, the level of unphosphorylated RNPII was dramatically reduced (Fig 1E and 1F), confirming that unphosphorylated RNPII undergone a dramatically elevated phosphorylation during sporangia differentiation, and the unphosphorylated RNPII may be further degraded in this process. Thus, we next explored how RNPII affected the morphological transition.

### PcDCL1 participated in sporangia differentiation and RNPII phosphorylation in *P*. *capsici*

**Fig 2:**
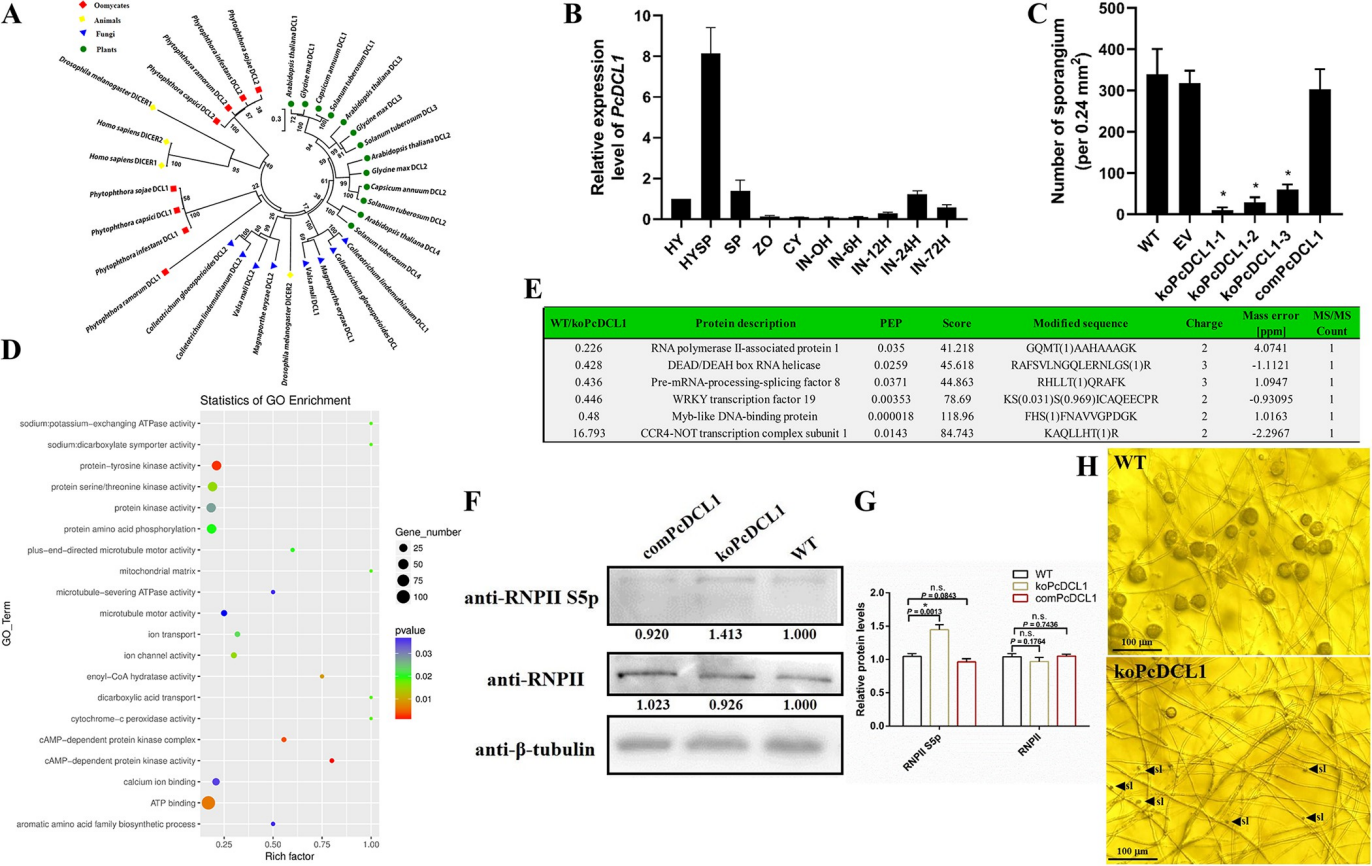
PcDCL1 is crucial for sporangia development in *Phytophthora capsici* through precisely phosphorylating certain proteins including RNPII. (A) Phylogenetic analysis of DCLs from different species. The phylogenetic tree was constructed by the neighbor-joining method. (B) The relative gene expression level of *PcDCL1* at different life stages and infective stages. HY: hypha; HYSP: sporangia-generated hypha; SP: sporangia only; ZO: zoospores; CY: cysts; IN-0H/6H/12H/24H/72H: *P*. *capsici* inoculated onto chili leaves for 0, 6, 12, 24 or 72 h. The expression level in the HY stage was set as 1.0 and relative gene expression level was calculated by the 2^-ΔΔCt^ method. The housekeeping gene *WS21* was used as an internal control. (C) Sporangia development was severely impaired in *PcDCL1* knockout mutants. The number of sporangia was measured and compared in wild-type *P*. *capsici* LT1534 (WT), empty vector isolate (EV), three *PcDCL1* knockout mutants (koPcDCL1-1/2/3), and a *PcDCL1* complementary isolate (comPcDCL1). Due to the relatively slower growth rate of *PcDCL1* knockout mutants (on average about 50% slower in koPcDCL1 than other isolates), for koPcDCL1-1/2/3, the sporangia measurement was performed 7 days after inoculation on V8 medium; for other isolates, the same experiment was performed 3.5 d after inoculation. Data are presented as the mean ± SD of 10 biological replicates. Statistical significance compared to the WT was determined using Student’s t-test (* *P* < 0.05). (D) Gene ontology analysis of the genes differently expressed in WT and koPcDCL1. The analysis was based on the molecular function of the genes. (E) Phosphoproteomics analysis showed that the representative proteins with the largest differences in phosphorylation levels between WT and koPcDCL1 were mainly involved in RNA processing and DNA binding. The phosphorylated proteins from the sporangia-generated hypha stage of WT and koPcDCL1 were extracted and detected by mass spectrometry. (F and G) RNPII Ser5 phosphorylation was elevated in koPcDCL1 than WT and comPcDCL1. The relative intensity of Ser5-phosphorylated RNPII and unphosphorylated RNPII in the sporulated hypha stage of WT, koPcDCL1, and comPcDCL1 was quantified with ImageJ (F). The WT band detected by each antibody was given a value of 1.00. Data presented in G are as the mean ± SD of three replicates. Statistical significance compared to the WT was determined using Student’s t-test (* *P* < 0.01). β-tubulin was used as the loading control and its intensity in each sample was used to normalize the data between samples. (H) The morphology of wild-type LT1534 and koPcDCL1 in sporulated hypha stage (HYSP). LT1534 was grown for 3.5 days and koPcDCL1 was grown for 7 days on V8 medium under light, respectively. Almost no canonical sporangium but a plenty of abnormal hyphal branches (ab; indicated by black triangles) that might be the precursors of sporangiophores were observed only in koPcDCL1 mutants.

Functional sRNAs play a crucial role in the process of morphogenesis across many species and participate in RNPII regulation [38]. DCLs serve as essential effectors for generating these sRNAs, prompting us to investigate their involvement in RNPII modulation and sporangia differentiation. Sequence analysis revealed that *Phytophthora* contains two *DCL* genes [39]. Phylogenetic analysis indicated that all *Phytophthora* DCL1 proteins are clustered into one clade and are close to canonical Dicers or DCLs in mammals, plants, and fungi, while *Phytophthora* DCL2 proteins are distinct from DCLs in other species (Fig 2A) and are not responsible for canonical miRNA processing (using miR8788 as an indicator; Fig B in S1 Text), which imply DCL1 is the main effector to process miRNAs in *Phytophthora*. Expression profiling showed that *PcDCL1* was significantly upregulated during sporangia formation (Fig 2B). The formation of sporangia starts with differentiation of hypha into sporangiophores, which are contiguous and similar in structure to vegetative hyphae. The tip of the sporangiophore expands to become a sporangium, followed by the movement of nuclei into sporangium. Interestingly, further knockout of *PcDCL1* (koPcDCL1) confirmed that it is indispensable for sporangia development in *P*. *capsici* (Figs 2C and C in S1 Text), plentiful sporangiophore-like structures were observed in koPcDCL1 (Fig 2H). Hypha growth, virulence and oospore production were also impaired in koPcDCL1, and koPcDCL1 was incapable of producing zoospores despite a few sporangia were formed (Fig D in S1 Text). Regarding sporangia production in inoculated plants, no sporangia were observed, probably because koPcDCL1 could rarely cause lesions in inoculated plants. These data indicated that PcDCL1 is an important regulator of sporangia development, only the precursor of sporangium, sporangiophore-like structures, could be formed from hypha without PcDCL1.

To discover the underlying mechanism of sporangia formation regulated by DCL1, RNA-seq was performed. As shown in Fig 2D, protein phosphorylation-related pathways were intensively changed in koPcDCL1 compared with WT. Furthermore, phosphoproteome analysis showed that many transcription-associated proteins, including RNPII-associated protein 1, comprised the phosphorylated proteins with the most significant intensity variation between koPcDCL1 and WT (koPcDCL1/WT > 2 or < 0.5, Fig 2E), implying that the phosphorylation state variation of the transcription executor, RNPII, induces phenotypic defects in koPcDCL1. As the initial and most crucial step of RNPII phosphorylation, we further confirmed that the RNPII S5p was greatly elevated in koPcDCL1, and unphosphorylated RNPII remain stable in the mutant (Fig 2F and 2G). All the data indicate that DCL1 could mediate sporangia formation through the modulation of RNPII phosphorylation state.

### PcamiR1 is a novel DCL1-dependent miRNA acted as key regulator of sporangia formation and RNPII CTD phosphorylation in *P*. *capsici*

**Fig 3:**
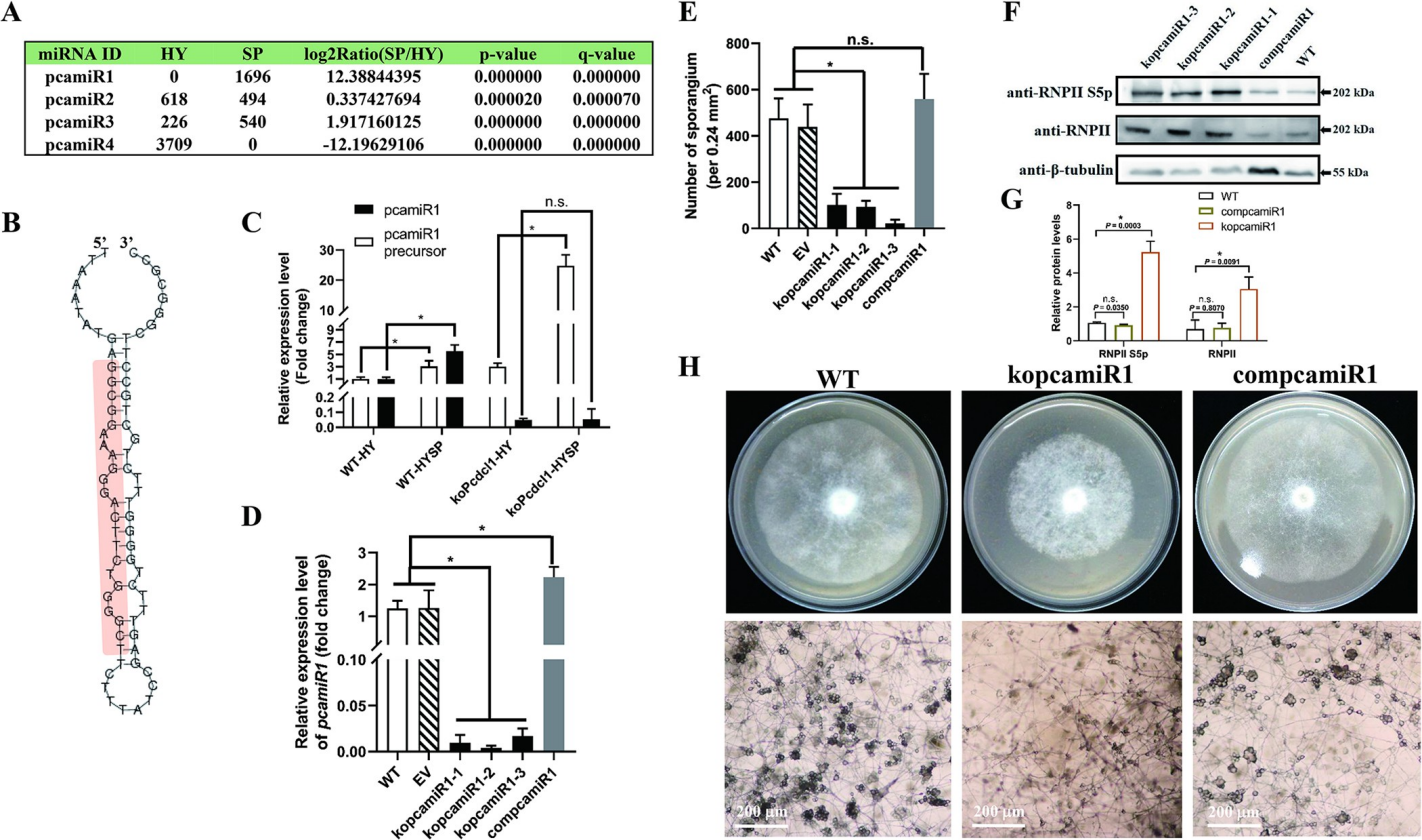
pcamiR1 is a novel DCL1-dependent microRNA that plays an important role in sporangia development in *P*. *capsici*. (A) Small RNA-seq revealed that four novel microRNAs were differentially expressed between the hypha stage (HY) and sporangia stage (SP). By secondary structure prediction of the DNA sequence from which small RNAs originated, pcamiR1 was identified as the only microRNA specifically and highly expressed in the sporangia stage. (B) The secondary structure of pcamiR1 precursor was predicted by Mirdeep and Mireap. (C) Relative gene expression level of pcamiR1 and its precursor in the hypha (HY) stage or sporulated hypha (HYSP) stage of wild-type *P*. *capsici* LT1534 (WT) and *PcDCL1* knockout mutant koPcDCL1. For each gene, the expression level of WT in the HY stage was given a value of 1.0 and relative gene expression level was calculated by the 2^-ΔΔCt^ method. The housekeeping genes *WS21* and *5S rRNA* were used as internal controls to quantify the pcamiR1 precursor and pcamiR1, respectively. (D) Relative gene expression level of pcamiR1 in the HYSP stage of WT, empty vector isolate (EV), kopcamiR1 mutants, and pcamiR1 complementary isolate compcamiR1. The expression level in WT was given a value of 1.0 and relative gene expression level was calculated by the 2^-ΔΔCt^ method. The housekeeping gene *5S rRNA* was used as an internal control. (E) Sporangia development was impaired in kopcamiR1 mutants. The number of sporangia in WT, EV, three pcamiR1 knockout mutants (kopcamiR1-1/2/3) and compcamiR1 was measured and compared. Due to the relatively slower growth rate of kopcamiR1 (on average about 30% slower in kopcamiR1 than other isolates), for kopcamiR1-1/2/3, the sporangia measurement was performed 6 days after inoculation onto V8 medium; for other isolates, the same experiment was performed 4 days after inoculation. Data in C, D, and E are presented as the mean ± SD of 3 (C and D) or 10 (E) biological replicates. Statistical significance compared to the WT or koPcdcl1 was determined using Student’s t-test (* *P* < 0.05). (F and G) RNPII Ser5 phosphorylation was significantly elevated in kopcamiR1 compared to WT and compcamiR1. Data presented in G are as the mean ± SD of three replicates. Statistical significance compared to the WT was determined using Student’s t-test (* *P* < 0.01). β-tubulin was used as the loading control and its intensity in each sample was used to normalize the data between samples. (H) Phenotypes of kopcamiR1. The upper lane shows the hypha growth status of WT, kopcamiR1, and compcamiR1; the lower lane shows the sporulated hypha growth status of the same isolates. The images were taken 6 days after kopcamiR1 was inoculated and 4 days after WT or compcamiR1 were inoculated.

Because DCL1 regulates the accumulation of miRNAs globally, we performed small RNA-seq (sRNA-seq) to examine the changes in miRNA expression during the transition from hypha growth to sporangia formation. Obviously different sRNA expression profiles were identified in these two stages, reflected by the different length distributions, nucleotide biases and genomic originations (Fig E in S1 Text). Except for miR8788, none of other miRNA is identified and validated in *Phytophthora*; thus, we mainly focused on the novel miRNAs discovered through a strict secondary structure prediction. As shown in Fig 3A, sRNA-seq revealed four novel miRNAs with more than 100 copies (low copies miRNAs may be gene fragments digested by RNase and without any function) in the hypha or sporangia stages of *P*. *capsici*. Among them, only pcamiR1 was highly expressed in the sporangia stage, whereas it could not be detected in the hypha stage, which implied that the developmental stage-specific miRNA is important for sporangia differentiation. The precursor of pcamiR1 (Fig 3B) was located in the noncoding region of scaffold_14: 73,272–73,346 (JGI database), and a promoter located in 73,051–73,100 was predicted via Promoter Scan, which indicates that the pcamiR1 precursor could be normally transcribed despite the lack of a protein-coding sequence. We further confirmed that pcamiR1 is a DCL1-dependent miRNA by detecting the abundance of the precursor and mature transcripts of pcamiR1 in both WT and DCL1-deficient *P*. *capsici* before or after sporangia formation by RT-qPCR and northern blotting, although northern blotting was not sensitive enough to detect the relatively low abundance of miRNA in *Phytophthora* compared with other species even after strong exposure (Fig F in S1 Text). As shown in Fig 3C, both the pcamiR1 precursor and mature transcript were significantly upregulated in the sporangia formation stage compared to the hyphal stage in LT1534 and were positively related. However, in koPcDCL1, the absence of mature pcamiR1 was accompanied by much higher amounts of precursor in the sporangia formation stage. These results clearly show that pcamiR1 is a canonical DCL1-dependent miRNA but not any other non-coding RNAs.

To confirm the role of pcamiR1 in sporangia differentiation, we generated pcamiR1 knockout mutant kopcamiR1 and the complementary mutant compcamiR1 by deleting or subsequently inserting the primary hairpin sequence of pcamiR1, respectively (Figs 3D and G in S1 Text). Compared with the WT and empty vector (EV) control, kopcamiR1 was defective and displayed significantly fewer sporangia (Fig 3E and 3H). Similar to koPcDCL1, there were also plentiful sporangiophores-like structures specifically observed in kopcamiR1 (Fig H in S1 Text). Conversely, compcamiR1, with a slightly higher expression level in the sporulated hyphae stage (1.8 fold compared to WT), displayed the same amount of sporangia as WT and EV (Fig 3D, 3E and 3H). These results demonstrate that alteration of pcamiR1 abundance affects sporangia development in *P*. *capsici*. Interestingly, like koPcDCL1 mutants, hypha growth, zoospore production, and virulence were also severely damaged in kopcamiR1 mutants (Figs 3H and G in S1 Text), and no sporangia emerged in kopcamiR1-inoculated plants due to its inability to cause any canonical lesions in the plant.

We then determined whether pcamiR1 regulates the phosphorylation of RNPII CTD. KopcamiR1 mutants showed markedly higher phosphorylation levels on RNPII CTD compared with WT and compcamiR1, while the unphosphorylated RNPII level was also increased in kopcamiR1 mutants (Fig 3F and 3G). Collectively, these results demonstrate that the regulation of RNPII CTD phosphorylation by pcamiR1 is critical for sporangia formation.

### PcamiR1 could efficiently repress *PcCDK7* translation through complexing with PcAGO1

**Fig 4:**
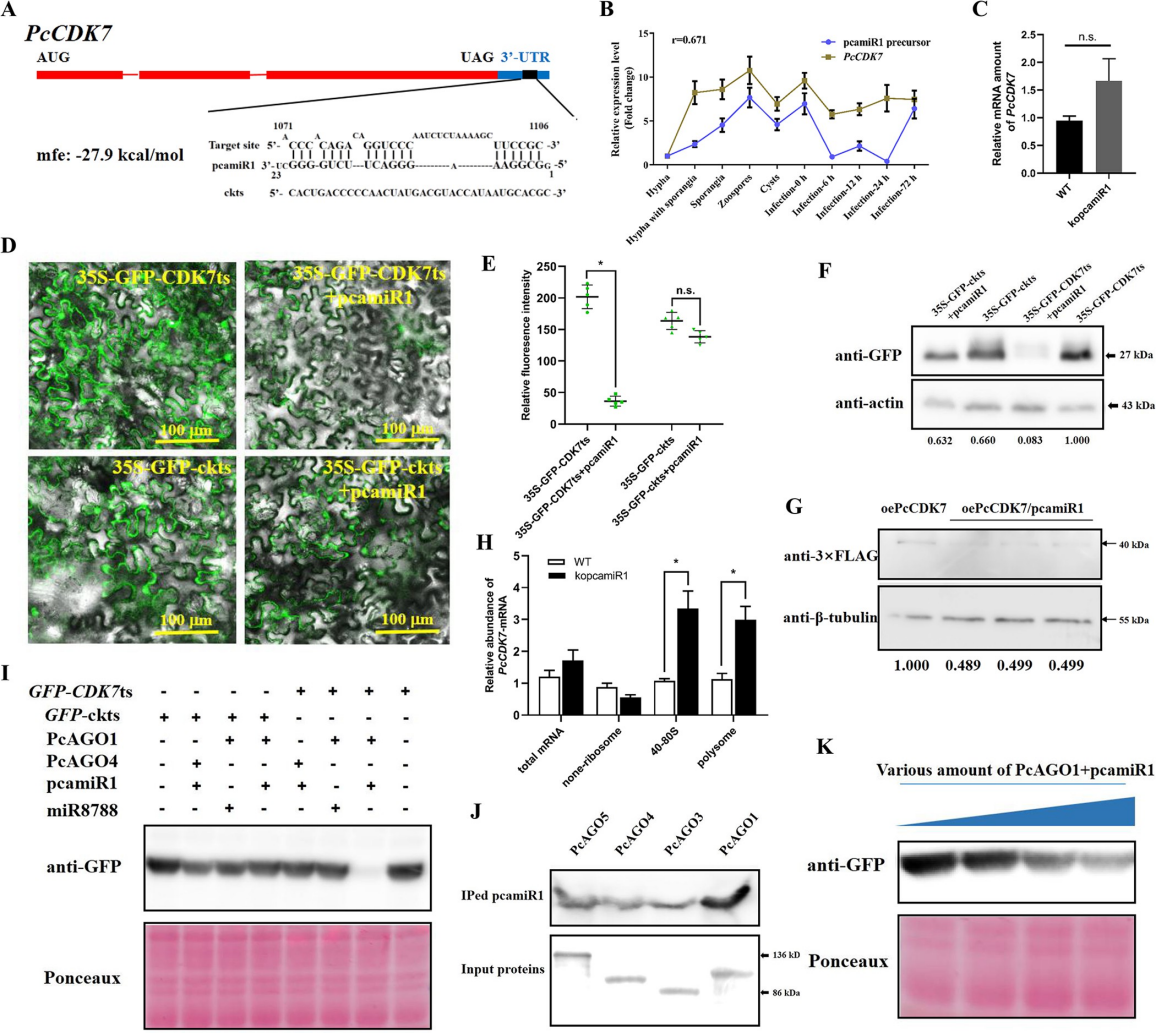
pcamiR1 could efficiently repress the expression of *PcCDK7* through ribosome occupancy associated translational inhibition. (A) Illustration of the predicted binding site between pcamiR1 and the 3’-UTR of *PcCDK7* or its mutated sequence (ckts). (B) The stringently positive correlation of the gene expression levels between *PcCDK7* and the pcamiR1 precursor at different life stages and infective stages. HY: hypha; HYSP: sporulated hypha; SP: sporangia only; ZO: zoospores; CY: cysts; IN-0H/6H/12H/24H/72H: *P*. *capsici* inoculated onto chili leaves for 0, 6, 12, 24 or 72 h. The expression level in the HY stage was given a value of 1.0 and relative gene expression level was calculated by the 2^-ΔΔCt^ method. The housekeeping gene *WS21* was used as an internal control. (C) Gene expression was not influenced in the pcamiR1 knockout mutant (kopcamiR1) compared to wild-type *P*. *capsici* LT1534 (WT). Statistical significance compared to the WT was determined using Student’s t-test (* *P* < 0.05). (D, E and F) The GFP tandem reporter system showed that the GFP signal was sharply weakened when the pcamiR1 target sequence of *PcCDK7* (CDK7ts) and pcamiR1 were co-expressed. Both GFP fluorescence (D and E) and GFP protein density (F) were assessed; 35S promoter-driven *GFP* was linked with CDK7ts or a target region mutated CDK7ts sequence (ckts), and pcamiR1 was expressed using a frequently used plant artificial miRNA expression vector (pEG-amiR171). The GFP signal was detected by confocal microscopy 2 days after *Nicotiana benthamiana* infiltration, the GFP intensity of five images of each treatment were calculated by ImageJ. Data are presented as the mean ± SD. Statistical significance compared to each control were determined using Student’s t-test (* *P* < 0.01). For western blotting, proteins from leaves were extracted and the house keeping protein actin was used as the loading control, its intensity in each sample was used to normalize the data between samples. The band intensity was calculated by ImageJ. Scale bar: 100 μm. (G) *In vivo* translational inhibition assay of pcamiR1 on *PcCDK7*. PcamiR1 was overexpressed in FLAG-tagged PcCDK7 overexpression isolate, the translated PcCDK7 protein was detected by western blotting. *P*. *capsici* house keeping protein β-tubulin was used as the loading control, its intensity in each sample was used to normalize the data between samples. The band intensity was calculated by ImageJ. (H) Ribosome occupancy is the key to result in the translational inhibition of pcamiR1 on *PcCDK7*. Relative abundance of *PcCDK7*-mRNA in total RNA, none-ribosome fraction, 40-80S ribosome fraction, or polysome fraction was calculated by the 2^-ΔΔCt^ method by comparing its abundance with the amount of the housekeeping gene *WS21* in corresponding fractions. One of the WT samples in each fraction was set as 1.0 and statistical significance was determined using Student’s t-test (* *P* < 0.05). (I and K) pcamiR1 could efficiently repress the translation of *PcCDK7* by binding to its 3’-UTR. The *in vitro* gene silencing assay was performed based on WGE. pcamiR1 and a control miRNA (miR8788), which is the only miRNA identified in *Phytophthora* so far (confirmed in *P*. *capsici* by small RNA-seq), PcAGO1 and the control protein PcAGO4, and GFP-CDK7ts and the control target site GFP-ckts were separately paired and equally incubated in the system. The GFP abundance was detected by western blotting. To further validate the translation inhibition effect, the RISC (pcamiR1 + PcAGO1) was added to an equal amount of GFP-CDK7ts DNA at different concentrations, and a positive relationship was found between the RISC concentration and gene silencing efficiency. The protein loading control is shown by Ponceau staining. (J) pcamiR1 preferentially bound to the argonaute protein PcAGO1. All the four canonical AGOs in *P*. *capsici* were labeled with 3×FLAG and expressed using the wheat germ cell-free expression system (WGE). pcamiR1 mimics were incubated with equal amounts of the four AGOs and co-immunoprecipitated after 3×FLAG bead enrichment. The abundance of pcamiR1 binding to each AGO protein was detected by northern blotting.

To explore how pcamiR1 modulates the phosphorylation state of RNPII CTD, we performed target prediction of all sequenced miRNAs in the hypha and sporangia stages of *P*. *capsici*, including pcamiR1 (all the potential target genes of pcamiR1 were listed in S1 Table). We found that among the RNPII CTD phosphorylation-related genes, only *CDK7* was potentially regulated by pcamiR1 through 3’-UTR binding (Fig 4A). The translational inhibition mediated by 3’-UTR imperfect match is rarely reported in species except animals. To explore the regulatory effect of pcamiR1 on *PcCDK7*, the expression correlation between *PcCDK7* and the pcamiR1 precursor was validated by RT-qPCR, which showed a strong positive correlation between the two genes (correlation coefficient = 0.671, Fig 4B). The data imply that pcamiR1 is a potential feedback regulator that precisely represses the overly upregulated *PcCDK7* during sporangia development.

To avoid the interference induced by endogenous interaction between pcamiR1 and *PcCDK7*, we test whether pcamiR1 could repress the expression of *PcCDK7* in plant cell, the 3’-UTR of *PcCDK7* containing putative pcamiR1 target site or the mutated target site was fused to *GFP* at its 3’ terminus (35S-GFP-CDK7ts or 35S-GFP-ckts, respectively) and transfected *Nicotiana benthamiana* with or without pcamiR1 expression (Fig F in S1 Text). The GFP signal and protein abundance were clearly decreased in 35S-GFP-CDK7ts-expressing plants after the expression of pcamiR1, while they remained stable in 35S-GFP-ckts-expressing plants whether pcamiR1 was expressed or not (Fig 4D, 4E and 4F), indicating that pcamiR1 could effectively inhibit the expression of *PcCDK7* possibly by complexing with *N*. *benthamiana* AGOs. To further explore whether pcamiR1 mediates gene silencing through target cleavage, RT-qPCR was performed to quantify the mRNA amount of *PcCDK7*. As shown in Fig 4C, *PcCDK7* mRNA was slightly upregulated in kopcmiR1 compared to the WT isolate; however, they did not differ significantly, implying that the mediation of gene silencing of *PcCDK7* by kopcmiR1 is independent of mRNA cleavage and translation repression may be crucial for pcamiR1-induced gene silencing. To confirm this, the protein expression level of PcCDK7 in pcamiR1-overexpression mutants was examined (Fig I in S1 Text). As shown in Fig 4G, pcamiR1 could significantly repress the expression of PcCDK7 *in vivo*. Moreover, as ribosome occupancy is the major factor to result in different translation efficiencies [40], here we confirmed that the rates of 40–80S ribosome and polysome occupied *PcCDK7*-mRNA in LT1534 were significantly less than that in kopcamiR1 mutants (Fig 4H), especially in the polysome portion, which usually contains the most actively translating mRNAs. These data strongly indicated that pcamiR1 could be an effective repressor of *PcCDK7* at translational level.

AGOs, the effector proteins of miRNAs, were indispensable for successful RNAi [41]. We firstly demonstrated that pcamiR1 could efficiently assembled with PcAGO1 but not the other three PcAGOs (AGO2 is absent in *P*. *capsici* genome compared with *P*. *infestans*; Fig 4J). PcAGO1 is the most conserved AGO protein in *Phytophthora*, and this result is consistent with the fact that canonical miRNAs have a tendency to load into AGO1 [42]. We then investigated the gene silencing efficiency of the pcamiR1-PcAGO1 complex using an *in vitro* translation system. The 3’-UTR of *PcCDK7* containing putative pcamiR1 target site or the mutated target site were fused to *GFP* at the 3’ terminus (GFP-CDK7ts or GFP-ckts) and then incorporated into pF3A, and the *in vitro* translation efficiency assay was conducted in different systems. Although the *PcCDK7* transcript amount was slightly higher in the absence of pcamiR1 (Fig 4C), the protein abundance was dramatically decreased in the contemporaneous presence of the pcamiR1-PcAGO1 complex and GFP-CDK7ts (Fig 4I). Meanwhile, the expression of GFP remained stable in other systems in which the pcamiR1-PcAGO1 complex and GFP-CDK7ts did not co-occur, either pcamiR1 was substituted with the known *Phytophthora* miRNA miR8788 or GFP-CDK7ts was substituted with GFP-ckts, even PcAGO1 was substituted with PcAGO4, all these combinations could release the translational repression induced by pcamiR1-PcAGO1 (Fig 4I). We further demonstrated that the translational inhibition effect was based on the abundance of the pcamiR1-AGO1 RISC complex *in vitro*, reflected by the observation that the more pcamiR1-AGO1 added, the better the gene silencing efficiency on *GFP* (Fig 4K). While the expression of GFP remained stable despite increasing amount of miR8788-AGO1 was added (Fig J in S1 Text). All these results strongly suggest that the translation of *PcCDK7* is repressed by pcamiR1 and involves RISC.

### PcCDK7 is a key regulator of sporangia formation in *P*. *capsici*

**Fig 5:**
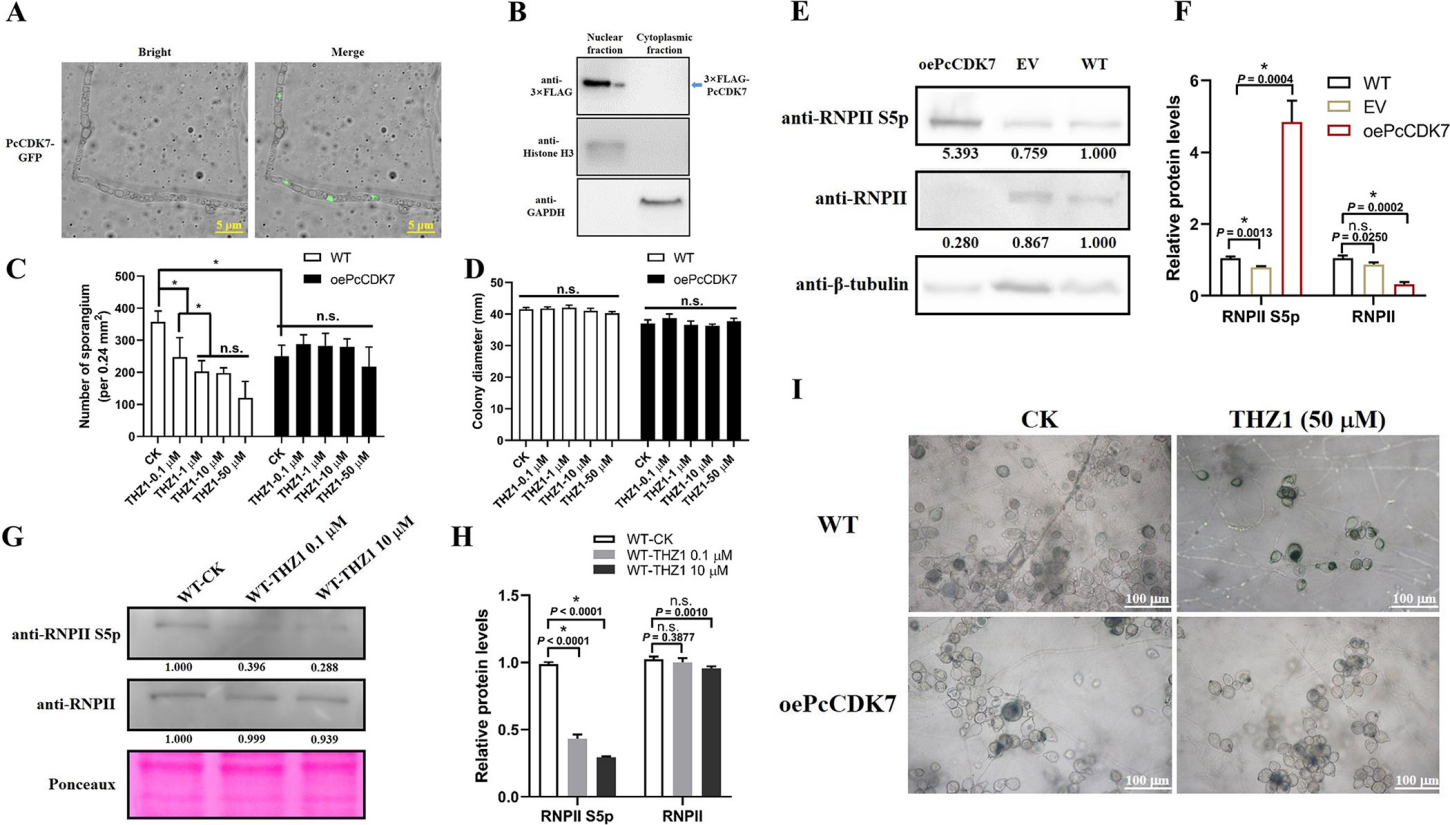
PcCDK7 directly phosphorylates RNPII and modulates sporangia formation in *P*. *capsici*. (A and B) PcCDK7 was localized in the nucleus. The subcellular localization of PcCDK7 was examined by confocal observation (A) and western blotting based on nuclear-/cytoplasmic-fraction isolation (B). Histone H3 and GAPDH were used as markers of nucleus and cytoplasm, respectively. (C) CDK7 inhibitor THZ1 had no effect on hypha growth in wild-type *P*. *capsici* LT1534 (WT) and the PcCDK7 overexpression isolate oePcCDK7. CK represents the DMSO treatment. (D and I) THZ1 could efficiently inhibit sporangia formation in WT but not in oePcCDK7. Sporangia development was impaired in WT grown on V8 medium with THZ1 added, while oePcCDK7 was not influenced by THZ1. Measurement and imaging were performed 4 days after the isolate was inoculated onto the medium. Data are presented as the mean ± SD of 10 biological replicates. Statistical significance compared to water treatment was determined using Student’s t-test (* *P* < 0.05). Scale bar: 100 μm. (E and F) RNPII Ser5 phosphorylation was elevated in oePcCDK7 than in WT and empty vector isolate (EV). The relative intensity of Ser5-phosphorylated RNPII and unphosphorylated RNPII in the sporangia-generated hypha stage of WT, EV, and oePcCDK7 was quantified with ImageJ. The WT band detected by each antibody was given a value of 1.00. Data presented in F are as the mean ± SD of three replicates. Statistical significance compared to the WT was determined using Student’s t-test (* P < 0.01). β-tubulin was used as the loading control and its intensity in each sample was used to normalize the data between samples. (G and H) THZ1 could repress RNPII Ser5 phosphorylation in *P*. *capsici*. LT1534 was grown on V8 medium with or without THZ1 for 5 days, the sporulated hypha was collected and used for protein extraction, and the relative intensity of Ser5-phosphorylated RNPII and unphosphorylated RNPII was quantified by western blotting. The WT-CK band detected by each antibody was given a value of 1.00. Data presented in H are as the mean ± SD of three replicates. Statistical significance compared to the WT-CK was determined using Student’s t-test (* P < 0.01). The protein loading control is shown by Ponceau staining.

PcCDK7 located in the nucleus of *P*. *capsici* (Fig 5A and 5B), which indicated that it could possibly interact with the canonical nuclear protein RNPII. To confirm the relationship between PcCDK7 and sporangia formation, we attempted to generate PcCDK7 knockout mutants. Possibly due to its indispensable role in cell cycle regulation, no homozygous *PcCDK7* knockout isolate could be obtained even after more than 10 attempts. Instead, a PcCDK7 overexpression isolate, oePcCDK7, was obtained after several attempts (Fig I in S1 Text). We then showed that the sporangium number was decreased by 30.07% in oePcCDK7 compared to WT (Fig 5D and 5I), and western blotting further confirmed that the phosphorylation state of the RNPII CTD was strongly elevated in oePcCDK7 (Fig 5E and 5F). Collectively, these results suggest that PcCDK7 is a direct RNPII phosphorylation modulator and hyper-phosphorylated RNPII could severely impair sporangia formation.

Recalling the known role of THZ1 in inhibiting the phosphorylation of the RNPII CTD by competitive binding to CDK7, we hypothesized that THZ1 could specifically inhibit sporangia formation in *P*. *capsici*. To address this, we measured the sporangia number in WT *P*. *capsici* LT1534 and oePcCDK7 in the presence of different concentrations of THZ1 compared to DMSO treatment (CK). As shown in Fig 5D and 5I, the production of sporangia was significantly impaired in LT1534 under THZ1 treatment, and the inhibitory effect was positively correlated with the drug amount. However, the sporangia formation of oeCDK7 was not affected by the same treatments, possibly due to the higher expression of the drug target CDK7 in the mutant compared to WT. Interestingly, the hypha growth rate was not impaired even when LT1534 or oeCDK7 were treated with high concentrations of THZ1 (Fig 5C), which further indicates that RNPII phosphorylation is closely associated with sporangia formation but not hypha growth. Meanwhile, western blotting confirmed that THZ1 could efficiently repress RNPII Ser5 phosphorylation but did not influence the abundance of unphosphorylated RNPII (Fig 5G and 5H). Together, these results demonstrate that CDK7 plays a key role in regulating the morphological transition from vegetative growth to sporangia formation through modulating RNPII phosphorylation.

### PcCDK7 interacted with and phosphorylated the unique RNPII CTD in *Phytophthora*, the effects were not strictly dependent on the specific sequence of heptapeptide repeats and Ser2/Ser5 were important for the precise phosphorylation

**Fig 6:**
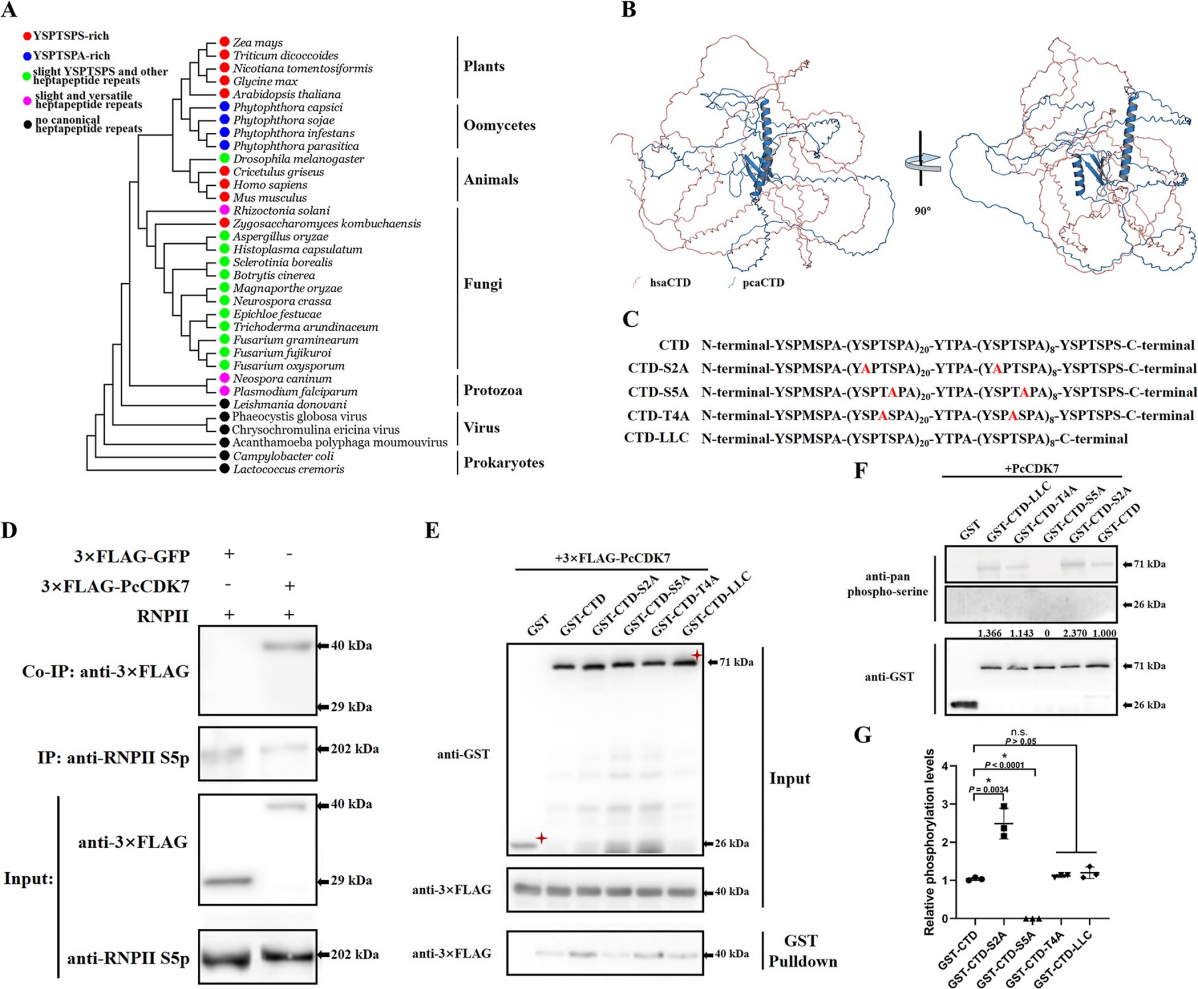
PcCDK7 interacts with the characteristic RNPII CTD and upregulates Ser5 phosphorylation in *Phytophthora*. (A) Phylogenetic analysis of the largest subunit of RNPII from different species. The RNPII CTD is located in the C-terminal of the largest subunit of RNPII, which harbors heptapeptide repeats in most higher organisms and *Phytophthora* species. The phylogenetic tree was constructed based on the amino acid sequences of the largest subunit of RNPII from different species with MEGA 5.0 using the neighbor-joining method. (B) Structural comparison of RNPII CTD from *Homo sapiens* (hsaCTD) and *Phytophthora capsici* (pcaCTD). About 400 amino acids of RNPII CTD from the two species were extracted and modeled by AlphaFold. HsaCTD has a loose and liner structure, while pcaCTD is more compact and could form canonical secondary structures in its central region. (C) Sketch map of the canonical *Phytophthora* RNPII CTD and four mutated RNPII CTDs. The red letters indicate mutated amino acids compared to the wild-type CTD. (D) The interaction between PcCDK7 and RNPII was verified via *in vivo* Co-IP assay. Total proteins for each sample were extracted from each isolate expressing 3×FLAG-PcCDK7 or 3×FLAG-GFP. The immune complexes were pulled down using anti-FLAG magnetic beads and immunoblotted with an anti-FLAG and anti-RNPII Ser5p antibody. 3×FLAG-GFP was used as the negative control. (E) PcCDK7 interacted with RNPII CTD regardless of amino acid substitutions or lack of the only canonical heptapeptide YSPTSPS. 3×FLAG-PcCDK7 and GST-CTD or the mutated GST-CTDs were expressed by *Escherichia coli* isolate BL21. The *in vitro* pull-down assay was performed using GST-sepharose beads after incubating PcCDK7 with GST-labeled proteins or GST. Anti-FLAG and anti-GST Ser5p antibodies were used to detect the interaction. GST was used as the negative control. (F and G) PcCDK7 could efficiently phosphorylate RNPII CTD unless the Ser5 in the heptapeptide repeats was mutated. A pan-phosphorylated-Ser antibody was used to detect the phosphorylation states of the native RNPII CTD and mutated CTDs after incubation with PcCDK7 (upper panel). Data presented in G as the mean ± SD of three replicates. Statistical significance compared to the GST-CTD was determined using Student’s t-test (* P < 0.01). Each protein sample was normalized by immunoblotting with the anti-GST antibody as the loading control (lower panel).

RNPII CTD located in the C-terminal of the largest subunit of RNPII, heptapeptide repeats harbored by RNPII-CTD are widespread in higher organisms but the sequence is versatile in different species. As shown in Fig 6A, the largest RNPII subunit of *Phytophthora* is closely related with higher organisms like animals and plants, both of which contain conserved heptapeptide repeats with only an amino acid substitution in the terminal position of the heptapeptide repeats (alanine in *Phytophthora* and serine in plants or mammals). Intriguingly, although the evolutionary status of *Phytophthora* is closer to other lower organisms like fungi, the RNPII CTD is more highly evolved in *Phytophthora* species, since no abundant canonical heptapeptide repeats exist in other lower species (Fig 6A). To better identify the structural difference of RNPII CTD between *Phytophthora* and mammals, ~ 400 amino acids of RNPII CTD from *Homo sapiens* (HsaCTD) and *P*. *capsici* (PcCTD) were extracted and their structures were predicted by AlphaFold (https://alphafold.ebi.ac.uk/). As shown in Fig 6B, HsaCTD is simply composed of long loops, it is incompact and may be able to provide more platforms for protein/nucleic acid binding. Interestingly, PcCTD contains two α-helix and three β-sheet in the central region, its volume and superficial area is smaller than HsaCTD, which make PcCTD more compact and differ from HsaCTD. Whether the uncanonical RNPII CTD of *Phytophthora* could affect its function is further studied.

In mammals, CDK7 could interact with and phosphorylates RNPII. However, whether the uncanonical heptapetide repeats YSPTSPA or further mutation of the repeats could affect the interaction remains enigmatic. To confirm this, an *in vivo* co-IP assay was performed. As shown in Fig 6D, 3×FLAG-fused PcCDK7 could be co-immunoprecipitated with the RNPII CTD after immunoprecipitation with anti-S5 phosphorylated RNPII beads but not the control 3×FLAG-fused GFP. The physical association between CDK7 and the RNPII CTD was further validated by an *in vitro* GST pull-down assay, which demonstrated that PcCDK7 could interact with RNPII CTD and even with its mutated proteins, which had an additional amino acid substitution in their multicopy heptapeptide sequence (S2A/S5A/T4A other than S7A compared with the canonical heptapeptide; Figs 6C, 6E, and K in S1 Text). Moreover, considering that only the last heptapeptide copy of the *P*. *capsici* RNPII CTD comprises the canonical YSPTSPS sequence, we also checked the interaction between PcCDK7 and the Loss of Last Copy RNPII CTD protein (CTD-LLC, with the deletion of the last copy of the heptapeptide YSPTSPS). As shown in Fig 6E, the deletion did not impair the interaction, which indicates that PcCDK7 could strongly interact with the RNPII CTD heptapeptide repeats regardless of some amino acid substitutions therein, and YSPTSPA repeats are sufficient for the interaction when replacing the canonical YSPTSPS repeats. To further explore whether this physical interaction could influence RNPII CTD phosphorylation, an enzymatic activity assay was performed. As shown in Fig 6F and 6G, purified RNPII CTD/CTD-S2A/CTD-T4A/CTD-LLC could all be phosphorylated by CDK7 *in vitro*, with RNPII CTD-S5A being the only exception; this result validates that CDK7 is a specific kinase of RNPII CTD Ser5. Interestingly, CTD-S2A was more intensively phosphorylated than WT CTD and other mutated CTD proteins, which implies that Ser2 is important for the precise phosphorylation of Ser5 in the CTD by PcCDK7. Collectively, these data indicate that PcCDK7 could interact with the unique RNPII CTD in *Phytopthora* and further phosphorylates it on Ser5, and some amino acid substitutions in the heptapeptide repeats did not affect the physical interaction between CTD and PcCDK7 but the Ser2/Ser5 mutation could impact the precise phosphorylation on CTD.

### Ser5 phosphorylation state of RNPII modulated by pcamiR1-CDK7 axis determines its chromosomal occupation, which then triggers the pronounced transcriptional plasticity during sporangia development and indirectly induced the overexpression of pcamiR1 and *CDK7*

The transcriptional plasticity during sporangia development in LT1534 and kopcamiR1 were examined. Unlike LT1534, the transcriptional profiles of hypha samples and sporulated hypha samples in kopcamiR1 were very close (correlation value > 0.972); the correlation coefficient (r) values were significantly greater than those between other samples (Fig L in S1 Text). This confirms that without pcamiR1, the phosphorylation states of RNPII were consistently high before or after sporangia formation and thus induce similar transcriptomic profiles in the two stages in kopcamiR1, which indicates that the phosphorylation state of RNPII is crucial for global transcriptional regulation. The data further indicate that three types of gene expression profiles could be delineated based on the transcriptome data: WT-HY represents the hypo-phosphorylation state of RNPII (hypo-PhosRNPII) and WT-HYSP represents the moderately phosphorylated state of RNPII (moder-PhosRNPII), while both kopcamiR1-HY and kopcamiR1-HYSP (with similar transcriptional profiles, Fig L in S1 Text) could represent the hyper-phosphorylated state of RNPII (hyper-PhosRNPII). The influenced pathways based on gene expression changes between WT-HY and WT-HYSP were analyzed previously (Fig 1C and 1G), and the pathway changes between WT-HYSP and kopcamiR1-HY/HYSP are shown in Fig M in S1 Text; catalytic pathways, cell signaling, and cell development were the main shifting pathways between the samples.

**Fig 7:**
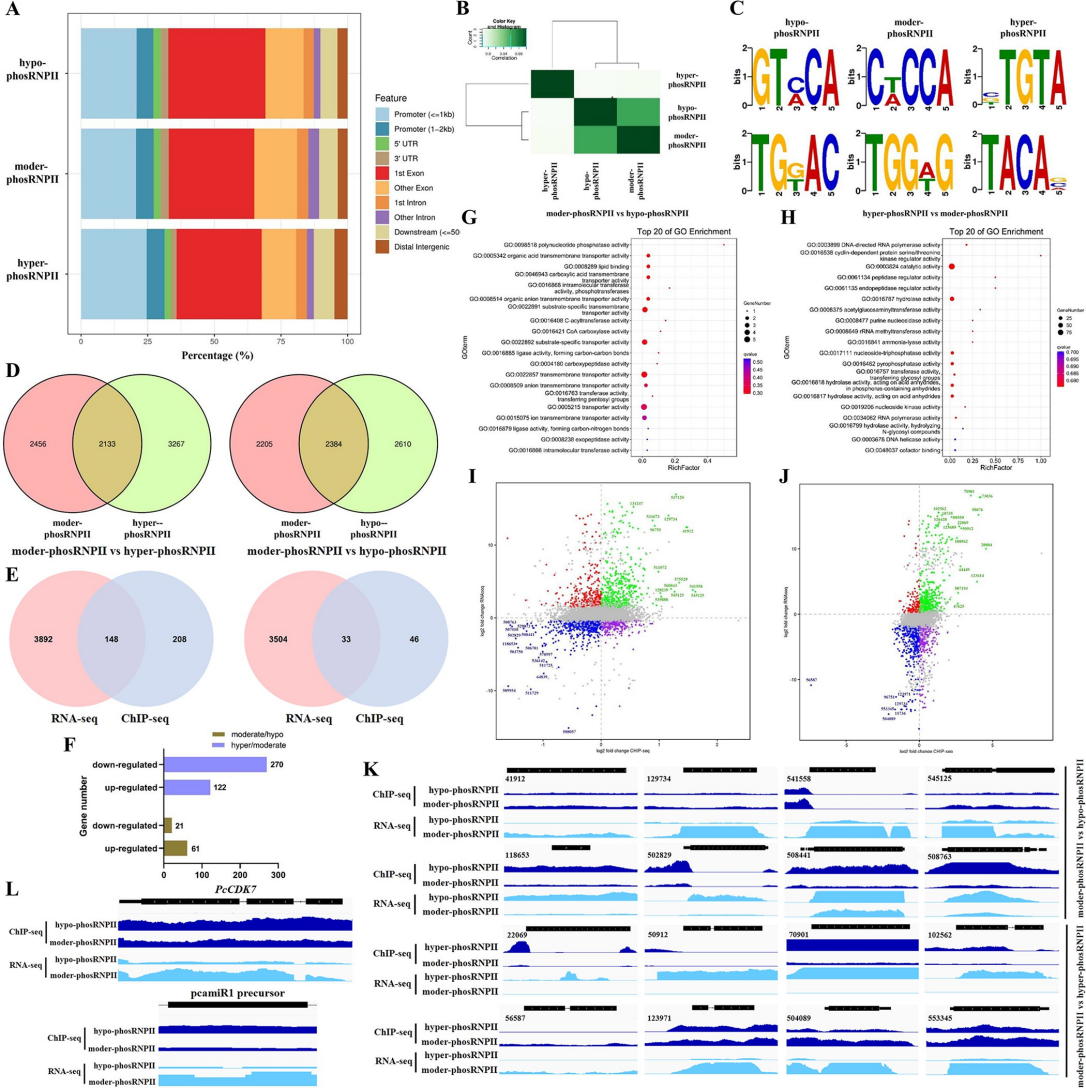
The phosphorylation state of RNPII determines its chromosomal occupation and induces different transcriptomes. (A) The phosphorylation state of RNPII determines its chromosomal occupation. Immunoprecipitated DNA from hypo-/moderately/hyper-phosphorylated RNPII was sequenced and identified. The distribution of the identified peaks in different genome regions was assessed by BWA and MACS. (B) Similarity analysis of the three levels of phosphorylated RNPII’s binding profiles. (C) Identification of the binding motifs of RNPII with different phosphorylation levels using the multiple EM for motif elicitation (MEME) program. The top two motifs for each phosphorylation level are shown. (D) Venn diagram showing the peak distributions in moderately phosphorylated RNPII vs hypo-phosphorylated RNPII or hyper-phosphorylated RNPII. The analysis was performed by MAnorm, a region of overlap ≥ 50% of the peak was considered the common peak, and peaks with |M| ≥ 1 and −log10^p-value^ ≥ 3 in the comparison were considered as differential peaks. (E) Venn diagram showing the number of genes both differentially expressed and differentially bound by RNPII in the two compared groups. Differentially expressed genes were defined as |log2^fc^| > 1 and FDR < 0.05 in an RNA-seq comparison. (F) The number of peak-related genes differentially bound by RNPII in the two compared groups. Peak-related genes were confirmed by their genomic location and gene annotation, the binding abundance was calculated based on the peak distribution in each gene. (G and H) Pathway enrichment analysis of genes differentially bound by RNPII in the two compared groups. The analysis was based on gene ontology analysis. (I and J) Four-quadrant diagram analysis showed the genes with the most significant changes in both RNA-seq and ChIP-seq. The labeled genes were uniformly variable in two sequencing datasets. Genes with log2^fc^ > 1 and M > 0.5 (moderate/hypo) or 1 (hyper/moderate) are labeled with green numbers and genes with log2^fc^ < −1 and M < −0.5 (moderate/hypo) or −1 (hyper/moderate) are labeled with blue numbers in the two compared groups. (K) Representative genes with the most significant changes in both RNA-seq and ChIP-seq in the two compared groups and (L) *PcCDK7* and pcamiR1 precursor gene in hypha (hypo-phosRNPII) and sporulated hypha (moder-phosRNPII) stages. ChIP-seq data (dark blue) and RNA-seq data (light blue) were depicted in the Integrative Genomics Viewer (IGV) browser. Black boxes represent the open reading frames of the genes.

RNPII lies the heart of transcription, its phosphorylation is the necessary prerequisite of active transcription. As S5p is the first step of RNPII-CTD phosphorylation and the phosphorylation on other sites follows RNPII-S5p’s initial chromosomal binding, while unphosphorylated RNPII is almost inactive in transcribing, here the chromosomal occupation of RNPII-S5p which could mostly represent the DNA binding preference of phosphorylated RNPII was studied to explore the actively transcribing genes in samples with different RNPII phosphorylation states. ChIP-seq assays of hypo-/moder-/hyper-PhosRNPII were performed. It was found that RNPII exhibited diverse occupations on different gene elements when phosphorylated at different levels. Additionally, compared to hypo-phosRNPII (which prefers the first exon region), moder-phosRNPII was significantly enriched in exons except for the first one; hyper-phosRNPII was more obviously located in the ≤ 1 kb region of genic promoters compared with moder-phosRNPII (Fig 7A). These results implied a transition from transcription initiation to transcription elongation during sporangia development, and hyper-phosphorylated RNPII was prone to transcribe new genes compared with hypo-/moder-phosRNPIIs. As shown in Fig 7B, enriched binding peaks in hypo-phosRNPII and moder-phosRNPII were more similar but differed greatly from peaks from hyper-phosRNPII. Moreover, similar binding motifs, GT(C/A)CA and C(T/A)CCA or TG(G/T)AC and TGG(A/T)G, were found in hypo-phosRNPII and moder-phosRNPII (Fig 7C). However, hyper-phosRNPII was more significantly enriched in the (C/G/T)TGTA or TACA(G/C/A) motifs, which further confirmed that the binding preference was dramatically changed when RNPII was heavily phosphorylated. Over 2,000 different or common peaks were identified in the moder-phosRNPII vs hyper-phosRNPII or hypo-phosRNPII groups (Fig 7D). After assigning these peaks to annotated genes, 21 and 270 downregulated genes and 61 and 122 upregulated genes, respectively, were identified in the moderate/hypo group or hyper/moderate group (Fig 7F). The differently expressed genes in the moderate/hypo group were mainly involved in the transportation and metabolism of ions, organic compounds, nucleotides, and lipids (Fig 7G), while the genes in the hyper/moderate group participated in transcription, cell signaling, and epigenetic pathways (Fig 7H). These data indicate that RNPII with different phosphorylation states have different binding preferences for various genes and their internal elements, inducing remarkable transcriptional changes during development.

The primary transcriptional change is directly induced by the chromosomal occupation of RNPII; thereafter, the primary upregulated or downregulated genes could provoke secondary transcriptional changes by regulating downstream gene expression, which gradually increases the variation in transcriptome. To identify the very upstream genes that triggered the significant transcriptomic changes in samples harboring hypo-/moder-/hyper-PhosRNPII and ultimately regulating sporangia development in *P*. *capsici*, a combined analysis based on ChIP-seq data and RNA-seq data was performed. As shown in Fig 7E, 33 and 148 genes were changed in both RNA-seq and ChIP-seq in the moder-phosRNPII vs hyper-phosRNPII or hypo-phosRNPII groups, respectively. To collectively analyze all the genes identified according to RNA-seq and ChIP-seq, a four-quadrant analysis was performed, which revealed that macromolecular binding, transferase activity, structural molecule activity, and heterocyclic compound binding were among the pathways containing the majority of genes differentially expressed and bound by RNPII during sporangia formation, while small molecule binding, transferase activity, and organic cyclic compound-binding-related genes were discretely bound and expressed when RNPII was hyper-phosphorylated compared to the moderate state (Figs 7I, 7J, and N in S1 Text). A positive correlation was found between the two high-throughput datasets, reflected by the location of more genes in the first and third quadrants and fewer in the second and fourth quadrants; this indicated that the binding abundance of RNPII on certain- gene was positive related to its expression level. We next extracted the genes with significant changes in both RNPII binding abundance and expression level (the most discrete genes in the four-quadrant diagram, mainly including genes with |log2^fc^| ≥ 1 in RNA-seq of both groups, with |M| ≥ 0.5 in the moder/hypo group and |M| ≥ 1 in the hyper/moder group for ChIP-seq, while *P* and FDR were both ≤ 0.01 in the two groups for RNA-seq and ChIP-seq). During sporangia development, the primary upregulated genes included sorting nexin-1, smok kinase, GAF genes, START genes, CRM1 exportin 1, histidine phosphatase, and 12-oxophytodienote reductase 1P, which are mainly involved in cell membrane formation, cell signaling, transportation of biomacromolecules (including lipid, proteins, and RNAs), and hormone synthesis (Table A in S1 Text and Fig 7K). The primary downregulated genes included *LRP1*, carbohydrate binding gene, pho-5, ferric reductase, fcyB, aquaporins, beta-glucosidase and three elicitins, which mainly participate in peroxide hydrolysis, deciphering glycans, and cellulose hydrolysis. The majority of the downregulated genes were involved in transportation (including sugars, oligosaccharides, amino acids, nucleosides, iron, and water) and, along with elicitins, indicated that glycan hydrolysis and virulence factors may be important for normal hypha growth in *P*. *capsici* (Table A in S1 Text and Fig 7K). Compared to moder-phosRNPII, the primary upregulated genes in hyper-phosRNPII were focused on epigenetic regulation (including retrotransposon, reverse transcriptase, and histone methyltransferase), cytoskeleton formation, MAPK signaling, and transcription regulation, while the primary downregulated genes were involved in nucleolar disassembly and reassembly, pre-mRNA splicing, transportation of metals, proteins or RNAs, and kinesin-related microtubule-dependent plus-end motion (Table B in S1 Text and Fig 7K). Taken together, RNPII phosphorylation state determined its chromosomal occupation preference and subsequently affected the expression of some primary genes, which then acted as initiators and amplified transcriptional changes during the morphological transitions of *P*. *capsici*.

In accordance with Fig 3C, the upregulation of *PcCDK7* and pcamiR1 precursor during sporangia development were further confirmed by RNA-seq (Fig 7L). Interestingly, the abundance of the two genes in the chromosomal immunoprecipitate of hypo-phosRNPII (RNPII in hypha stage) were both slightly higher than that in moder-phosRNPII component (RNPII in sporulated hypha stage; Fig 7L). These indicated the overexpression of *PcCDK7* and pcamiR1 during sporangia development was not directly triggered by the primarily transcriptional regulation through RNPII binding, instead, secondary regulators like some transcription factors, may upregulated by RNPII and subsequently leaded to the overexpression of the two genes during the morphological transition. Moreover, the spontaneously jointly upregulation and further inhibition of pcamiR1 on *PcCDK7* expression could be an efficient feedback regulatory machinery to ensure the precise phosphorylation state of RNPII.

### Application of pcamiR1 antagomir was a promising strategy for efficient disease control

**Fig 8:**
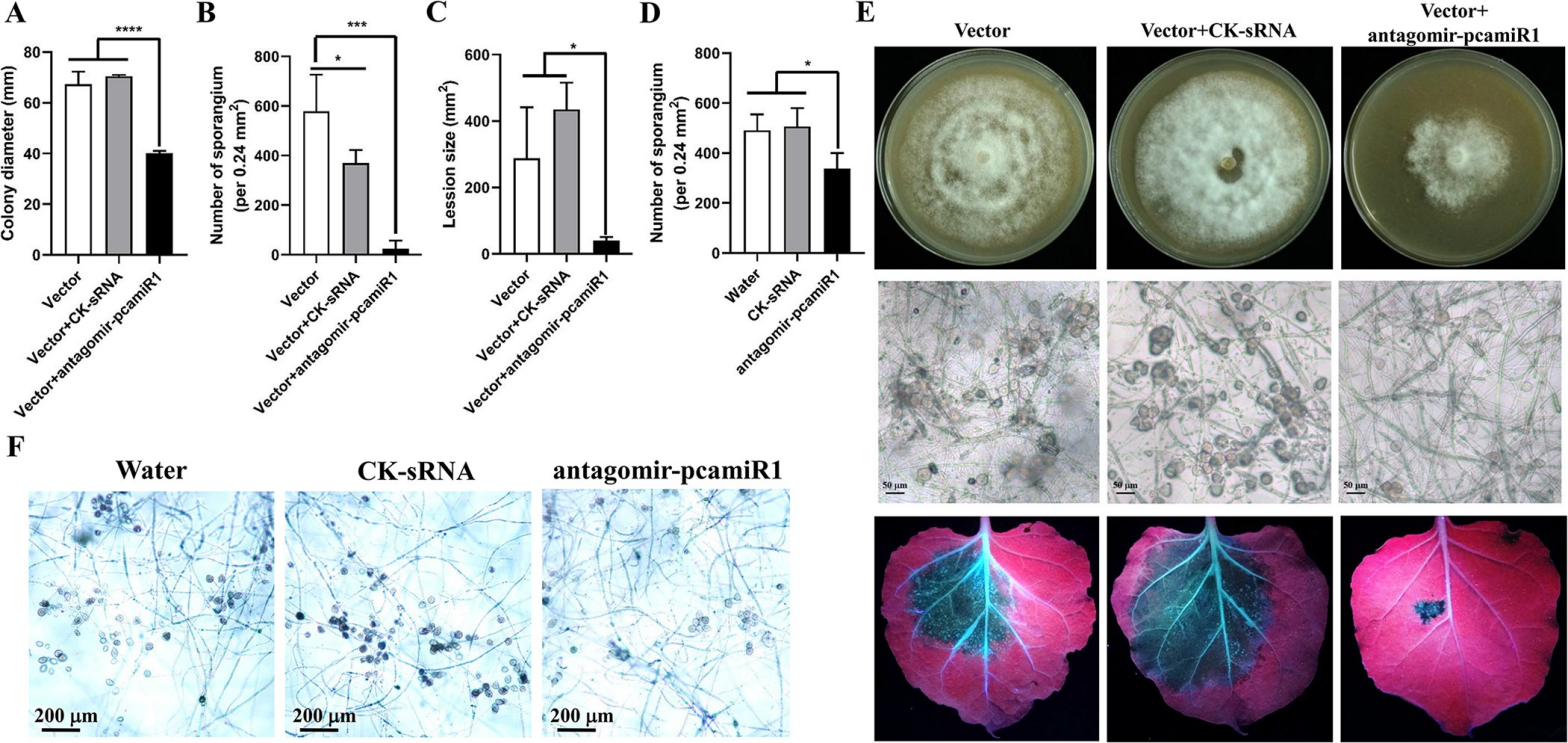
Application of pcamiR1 antagomir could be a promising strategy for *Phytophthora capsici* control. (A, B, C and E) Hypha growth rate (A), sporangia production (B), and virulence (C) of *NPTII*-harboring vector transformants (Vector) or transformants of *P*. *capsici* harboring vector and pcamiR1 antagomir sRNA or control sRNA (CK-sRNA). Colony diameter was measured (A) and photos were taken (upper panel in E) after *P*. *capsici* was grown on V8 medium for 3 days. Sporangia were numerated from five randomly selected fields of view under a microscope for three different isolates of each type of transformant (B), and the enumeration and imaging (middle panel in E) were performed 5 days after Vector or CK-sRNA isolates were grown on V8 and 10 days after pcamiR1 antagomir isolates were grown on V8. Scale bar: 50 μm. Mycelial plugs were used to inoculate detached leaves of *Nicotiana benthamiana*, and lesion size was measured (C) and photos were taken (lower panel in E) at 3 dpi under UV to better visualize the lesions. All the data in A, B, and C are presented as the mean ± SD. Statistical significance compared to the vector and vector + CK-sRNA was determined using Student’s t-test (* *P* < 0.05, *** *P* < 0.001, **** *P* < 0.0001). (D and F) Sporangia development was severely impaired after *in vitro* application of pcamiR1 antagomir sRNA on *P*. *capsici*. Sporangia were numerated from 10 randomly selected fields of view under a microscope for wild-type *P*. *capsici* LT1534 after treatment with water, 200 nM control sRNA (CK-sRNA) or pcamiR antagomir sRNA. Enumeration (D) and imaging (F) were performed 6 days after treatment in liquid V8. Scale bar: 200 μm. Data are presented as mean ± SD. Statistical significance compared to water and CK-sRNA treatment was determined using Student’s t-test (* *P* < 0.05).

Having confirmed that pcamiR1 is essential for *P*. *capsici* development, to test whether the inhibition of pcamiR1 holds promise for the control of plant diseases caused by *P*. *capsici*, we directly introduced synthesized pcamiR1 antagomir (a miRNA mimic with the reverse-complementary sequence of pcamiR1, which is predicted to exclusively target pcamiR1 in *P*. *capsici* without affecting any other genomic element or miRNA) into *P*. *capsici* to mimic the effects of *in vitro* application of miRNA antagomir. The synthesized sRNA was introduced into *P*. *capsici* together with a plasmid conferring resistance to G418; transformants that gained G418 resistance potentially also took up the target sRNAs. As controls, plasmid only or plasmid combined with a sRNA with a scrambled pcamiR1 sequence were also introduced into *P*. *capsici*. Compared with transformants harboring plasmid and control sRNA, transformants harboring pcamiR1 antagomir exhibited a moderate decrease in mycelial growth and significant defects in sporangia development even though the pcamiR1 antagomir transformants were cultivated for a longer time than other isolates as they grew more slowly. Importantly, the introduction of pcamiR1 antagomir nearly abolished the virulence of *P*. *capsici* (Fig 8A, 8B, 8C, and 8E). These data indicate that direct inhibition of pcamiR1 could be effective in reducing the survival fitness of *P*. *capsici*. Because *P*. *capsici* has the ability to take up environmental sRNAs [43], we further tested the effect of the antagomir applied *in vitro* on *P*. *capsici*. As shown in Fig 8D and 8F, a > 30% reduction in sporangia number was observed in antagomir pcamiR1-treated *P*. *capsici* LT1534 compared with water or control sRNA treatments. Taken together, due to the important role of pcamiR1 in *P*. *capsici*, the pcamiR1-targeting antagomir-based strategy could be promising for the control of plant diseases caused by *P*. *capsici*. Besides, these data further indicate pcamiR1 is dispensable for development and infection in *Phytophthora*.

### The miRNA-CDK7-RNPII regulatory module was conserved

**Fig 9:**
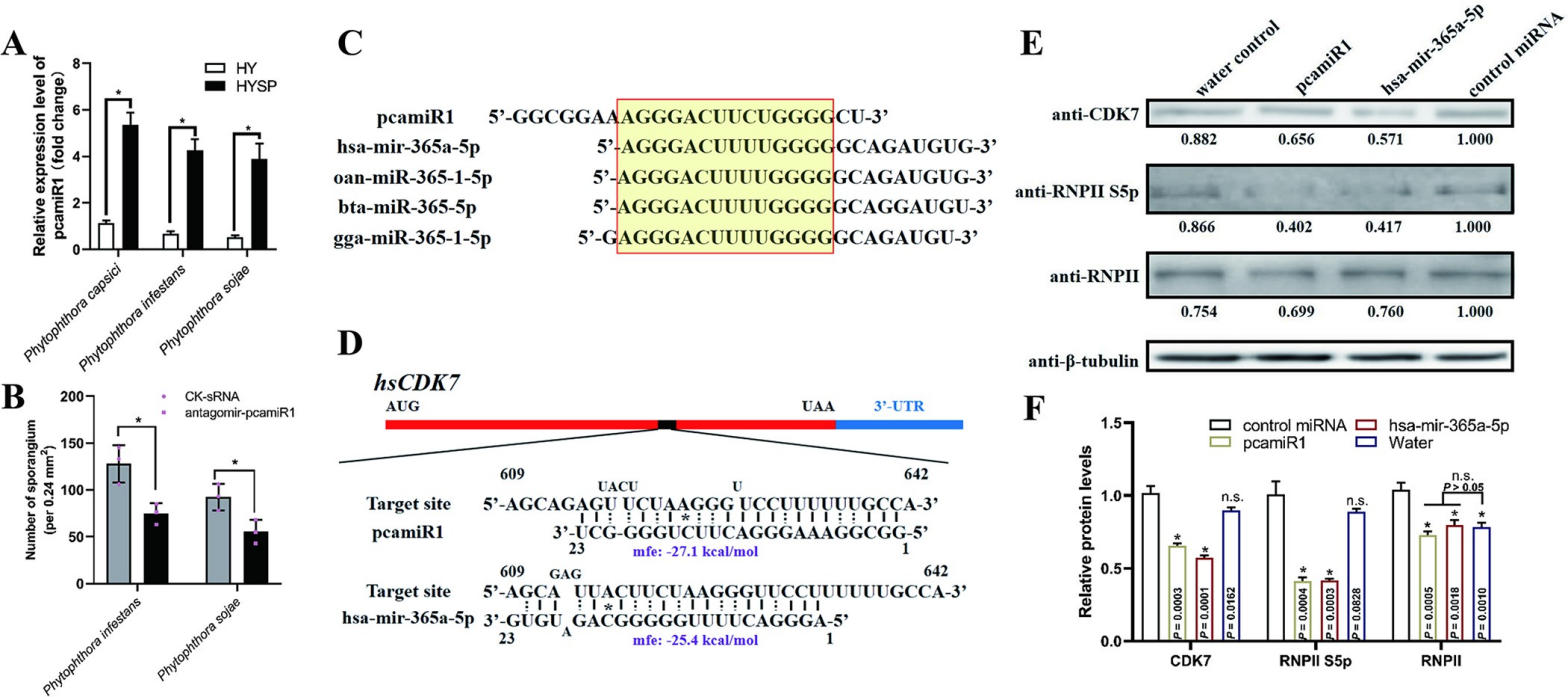
The microRNA-CDK7-RNPII module is conserved. (A) pcamiR1 is conserved in *Phytophthora*. PcamiR1 was detected in hypha (HY) or sporulated hypha (HYSP) stage of *Phytophthora capsici*, *Phytophthora infestans*, and *Phytophthora sojae*, respectively. The expression level of *P*. *capsici* in the HY stage was given a value of 1.0 and relative gene expression level was calculated by the 2^-ΔΔCt^ method. The housekeeping gene 5S rRNA were used as internal controls to quantify pcamiR1. (B) Sporangia development was severely impaired after *in vitro* application of pcamiR1 antagomir sRNA on *P*. *infestans* and *P*. *sojae*. Sporangia were numerated from 10 randomly selected fields of view under a microscope after treatment with 200 nM control sRNA (CK-sRNA) or pcamiR antagomir sRNA. Data are presented as mean ± SD. Statistical significance compared to CK-sRNA treatment was determined using Student’s t-test (* *P* < 0.05). (C) pcamiR1 analogues were identified in different species belong to animalia. The pcamiR1 analogue miR-365a-5p and miR-365-1-5p were identified in 14 species by searching in miRBase 22.1. A part of the miRNAs are shown and the identical sequences among different miRNAs are highlighted. hsa: *Homo sapiens*; oan: *Ornithorhynchus anatinus*; bta: *Bos taurus*; gga: *Gallus gallus*. (D) Base pairing and predictive binding energy of pcamiR1 or has-mir-365a-5p with their predicted target sites in *hsCDK7* in *H*. *sapiens*, respectively. (E and F) *CDK7* expression and RNPII Ser5 phosphorylation were inhibited by pcamiR1 and hsa-mir-365a-5p. The relative intensity of hsCDK7, Ser5-phosphorylated RNPII, and unphosphorylated RNPII was quantified 48 h after transfection by ImageJ (E). The control miRNA treated samples detected by each antibody was given a value of 1.00. Data presented in F are as the mean ± SD of three replicates. Statistical significance compared to the control miRNA sample was determined using Student’s t-test (* *P* < 0.01). β-tubulin was used as the loading control and its intensity in each sample was used to normalize the data between samples.

MiRNA is well known for its evolutionary conservation among different species. Here we confirmed that the mature pcamiR1 was also presented in other *Phytophthora* species (*Phytophthora infestans* and *Phytophthora sojae*), both with a much higher expression level in sporangia formation stage than hypha-only stage (Fig 9A); but it could not be detected in the fungi *Fusarium graminearum*, *Botrytis cinerea* and *Magnaporthe grisea* (Table C in S1 Text), which indicated that pcamiR1 is a *Phytophthora*-specific miRNA. Moreover, the treatment of *P*. *infestans* and *P*. *sojae* hypha with exogenous antagomir-pcamiR1 could significantly induce the impairment of sporangium development (Fig 9B), which is consistent with that in *P*. *capsici* (Fig 8D and 8F). This indicated that pcamiR1 could be functional in sporangia formation in all *Phytophthora* species. To further investigate whether the involvement of the miRNA-CDK7-RNPII regulatory module is conserved in transcriptional control in other species, we BLAST-searched pcamiR1 in miRBase 22.1 (http://www.mirbase.org/). Probably because miRNA is rarely reported in species closely related to *P*. *capsici*, only a mammal-specific miRNA, miR-365-5p, was identified as the analogue of pcamiR1 in 14 mammalian species; its function remains largely unknown. Hsa-mir-365a-5p is the analogue of pcamiR1 in *Homo sapiens*, with a similar sequence for 14 nt (Fig 9C). Bioinformatics analysis revealed that both pcamiR1 and hsa-mir-365a-5p could strongly bind to the coding sequence of *hsCDK7* (mfe < −25 kcal/mol, Fig 9D). This differs from most functional miRNAs in mammals, which mainly target the UTR sequence of target genes and subsequently inhibit their translation. To test whether pcamiR1 and hsa-mir-365a-5p could modulate RNPII CTD phosphorylation by repressing the expression of *hsCDK7*, we transfected human 293T cells with pcamiR1 and hsa-mir-365a-5p. The expression of hsCDK7 was more severely reduced in 293T cells after pcamiR1 and hsa-mir-365 treatment than control miRNA treatment (Fig 9E and 9F). Furthermore, the phosphorylation of the RNPII CTD also specifically decreased in line with hsCDK7 accumulation (Fig 9E and 9F). Meanwhile, the downregulation of hsCDK7 expression and the phosphorylation state of RNPII after pcamiR1/hsa-mir-365a-5p treatment were both not as significant as in *P*. *capsici*, probably because the miRNA binding sites were not located in the conserved 3’-UTR of the gene and thus impaired the silencing efficacy. These data provide genetic evidence that the novel miRNA-CDK7-RNPII transcriptional regulatory pathway is conserved and novel microRNA-based regulation could be promising for the control of diseases caused by disordered RNPII phosphorylation. Meanwhile, the weak effect of pcamiR1 in mammal cell imply that it could be a safe fungicide when be properly modified and applied.

## Discussion

RNPII is recruited to actively transcribing genes when its CTD is properly phosphorylated [44]. According to our data, sorting nexin, smok kinase, histidine phosphatase, and four START-like domain-harboring genes are preferably bind and transcribed by moderately phosphorylated RNPII, which implies that membrane coat assembly, signal transduction, and the cellular transportation of many materials, especially lipids and sterols, are important for sporangia formation in *Phytophthora*. Kinases and sterols are usually involved in signal transduction [45], and their upregulation may be the key to initiate the transcriptomic changes during sporangia development. Moreover, as lipids and sterols are the precursors of hormones, the central upregulation of START-like domain-harboring genes may be essential for hormone synthesis, which mediates the morphological transition in *Phytophthora* [46]. Interestingly, many aquaporin genes (*AQPs*) and elicitin genes were the primary genes to form the transcriptional pattern in the hypha growth stage. AQPs are associated with growth in plants, which is accompanied by cell division or differentiation and high water consumption [47]. Hypha is a fast vegetative growth stage in filamentous microorganisms, and many nutrients, especially water, are heavily required at this stage, AQPs may play a key role in water ingestion and utilization in hypha growth in *Phytophthora*. On the other hand, elicitins are well known for inducing hypersensitivity responses in plants, such as INF1 in *P*. *infestans* [48]. Similar to our findings, the highest expression levels of *INF1* were also observed in hypha grown of *P*. *infestans in vitro* [48]. This implies that elicitins have potential role in hypha growth apart from virulence in *Phytophthora*, which may possibly related to their function in exogenous sterol transportation and cell membrane formation in the sterol-auxotrophic organisms [49]. Widely different from moderately/hypo-phosphorylated RNPII, hyper-phosphorylated RNPII is enriched in many epigenetic pathway-related genes, such as three reverse transcriptase genes and four chromosomal modification-related genes. Reverse transcriptase is the key enzyme required for the conversion of RNA to DNA, while chromosomal modification genes could determine whether the local chromatin assumes a more compact or more relaxed state [50]. The preference to bind and express these genes when RNPII is hyper-phosphorylated indicates that epigenetic regulation could be a compensatory effect when RNPII is improperly heavily phosphorylated. However, the exact roles of these genes in the development are mysteries, they could be ideal targets to design *Phytophthora*-specific antimicrobials due to their indispensable roles.

The core regulator of large-scale transcriptional or proteomic changes during specific organ development is poorly studied [51]. RNPII could determine the primarily induced genes and then provokes secondary transcriptional changes, which were gradually creates transcriptional profiles. The identification of the upstream regulator of the transcriptional changes during *Phytophthora* sporangia development has great significance because it could benefit the elucidation of complex transcriptional networks and identify the key inducer of this morphological transition. Meanwhile, the rapid elevation of the RNPII-CTD S5p during the morphological transition of *Phytophthora* was accompanied by remarkable transcriptional changes, which triggered by the chromosomal occupation preference of differentially phosphorylated RNPII. Numerous ChIP-seq studies revealed that RNPII-S5p is enriched at the promoter proximal region [52], but it is also present during elongation and co-transcriptional splicing besides transcription initiation [53]. The present study discovered that the S5p level dominates the chromosomal occupation of RNPII and further affects its transcription. These findings indicate that S5p located in the unique heptapeptide repeats of RNPII CTD has an important role in transcriptional regulation and provides a novel model of the importance of the characteristic RNPII modulation in development, which could further contribute to the design of better control strategies on the pathogens.

RNPII is the core element of transcription machinery. The CTD of RNPII is extremely important for its function since it could serve as a scaffold to interact with different proteins to participate in multiple functions [54]. The various phosphorylation states of the RNPII CTD coordinate co-transcriptional processes with the stages of transcription [15]; for example, the transition of RNPII from initiation to elongation is accompanied by phosphorylation of the CTD by CDK7 and CDK9 [55]. Phosphorylation of the CTD has been shown to affect its interaction with hydrogels formed by the low-complexity domains of FUS, EWS and TAF15 proteins, which indicates that phosphorylation could affect the condensate-interaction properties of the RNPII CTD [56]. Different CTD phosphorylation patterns have been proposed to orchestrate the transcription of virtually all genes through complex interactions with different mediators, splicing factors or other proteins [16,53]. However, the manner in which specific proteins interact with RNPII CTD having different phosphorylation states in *Phytophthora* to control expression is worth further elucidation, especially considering that heptapeptide repeats located in the *Phytophthora* RNPII CTD differ from those in animals or plants. We found that single or dual amino acid mutations in the CTD did not affect the interaction between RNPII and PcCDK7, which indicated that the physical interaction is largely rely on the heptapeptide repeats but not their exact sequence. Moreover, Ser2 or Ser5 (the modified sites of CDK7) mutations could impair the phosphorylation of the CTD, confirming that the second and fifth serine in RNPII CTD heptapeptide repeats are crucial for the proper phosphorylation of RNPII. However, the phylogenetic origination of the uncanonical heptapeptide repeats in *Phytophthora* and whether the variation in the repeats could induce changes in the protein interactome of RNPII warrant further study.

In the study, we found that unphosphorylated RNPII was elevated in kopcamiR1 mutants but declined in the oePcCDK7 isolate compared to WT *P*. *capsici*. It is readily comprehensible that PcCDK7 could more efficiently transform the unphosphorylated RNPII to the hyper-phosphorylated state and cause the reduction of the unphosphorylated proteins. Interestingly, RNPII and RNPII S5p abundance in kopcamiR1 were more dramatically elevated than koPcDCL1 when compared with wild-type *P*. *capsici*, respectively, which imply that pcamiR1 is a more specific regulator of RNPII. However, the accumulation of unphosphorylated RNPII in kopcamiR1 remains enigmatic; presumably, the deficiency of pcamiR1 could lead to the overexpression of RNPII, and thus, the total amount of RNPII in kopcamiR1 was increased regardless of phosphorylation states. Although studies in mammalian cells have indicated that hyper-phosphorylated RNPII is preferentially ubiquitinated and more likely to be involved in proteasome-dependent degradation [57], whether the elevation of unphosphorylated RNPII in kopcamiR1 compared to wild-type *P*. *capsici* is associated with protein degradation, as well as the mechanism of overexpression of RNPII in kopcamiR1, need to be further studied.

It has been reported that CTD kinases and phosphatases, along with GTPase, are associated with CTD phosphorylation during transcription [15]. However, the direct regulator of CTD phosphorylation is rarely reported. Here, we confirmed that pcamiR1 could efficiently modulate CTD phosphorylation and the pcamiR1-CDK7-RNPII axis is conserved in transcriptional regulation in different species. The existing studies on the function of miRNAs in plant pathogens mainly focused on their role in the cross-kingdom modulation of immunity gene in plants, and their silencing activity was based on miRNA-mRNA perfect matching and target degradation [8]. Here, we discovered that miRNAs of microbes could also induce translational repression of autologous target genes through imperfect binding like in animals, which could broaden our understanding of gene silencing in lower eukaryotes and further benefit the exploration of newly discovered miRNA targets in microbes. Moreover, pcamiR1 is upregulated in line with *PcCDK7* during sporangia development independent of directly RNPII regulation, and through further silencing of *PcCDK7* via complexing with PcAGO1, the direct model of RNPII phosphorylation modulation could provide a rapid pathway to control the dynamic CTD phosphorylation state and ensure appropriate gene transcription. This post-transcriptional-level regulatory module enables the RNPII phosphorylation regulation more direct and faster than the well-known classical model (Ras/Rho-MAPK-transcription factors) [19]. Moreover, the positive correlation between the pcamiR1 precursor and *PcCDK7* in different life stages of *P*. *capsici* further confirms that pcamiR1 is indispensable for the dynamic control of the important kinase CDK7. However, as cell signaling and hormone sensing are involved in sporangia development in *Phytophthora* [25], whether they induce the constitutive heavy upregulation of *PcCDK7* and pacmiR1 during the morphological transition remains to be explored.

CDK7 is a component of the CDK7-CyclinH-MAT1 complex, which is involved in cell cycle control, transcription, and DNA damage repair [58]. The phosphorylation of RNPII CTD Ser5 and Ser7 (Ser7 does not exist in *Phytophthora*) by CDK7 could modulate transcriptional initiation, elongation, and termination by interacting with different factors [59]. Because its dysfunction could induce malignant tumors such as breast cancer, CDK7 inhibitors including flavopiridol, roscovitine, and THZ1 [60] are important anti-tumor drugs. In the current study, we confirmed that pcamiR1 and its analogue are efficient in regulating CDK7 expression, which indicates that anti-tumor drugs designed by noncoding RNAs have great potential. Cross-kingdom microRNA-mediated gene expression regulation is confirmed in humans/mice, showing that plant miRNA MIR168a could bind to low-density lipoprotein receptor adapter protein 1 (LDLRAP1) mRNA in mammals, inhibit LDLRAP1 expression and consequently decrease low-density lipoprotein removal from plasma [61]. Additionally, the abundance of dietary plant-derived miR159 in the serum was inversely correlated with breast cancer incidence and progression in patients, and oral administration of a miR159 mimic significantly suppressed the growth of xenograft breast tumors in mice [62]. Therefore, exogenous miRNAs could be potential medications with high sequence specificity (different from endogenous miRNAs that target various genes in a certain mammal) and low development cost, making them a hot topic in commercial therapeutics design and development, with research into the application of MRX34, miravirsen, and others already ongoing [63]. As shown in this study, the *in vitro* application of pcamiR1 antagomir could efficiently repress sporangia formation in *P*. *capsici*, which could strongly impair its virulence due to the importance of sporangia in the disease cycle. Considering the importance of CDK7 in cell cycle control, whether the system could be effective in treating human diseases warrants further exploration. Another miRNA, pcamiR4, is acts as a hypha-specific miRNA, whether it is involved in the hypha-sporangia transition and could be used as a potential control target remain to be further studied. Moreover, despite pcamiR1 analogues are existed in mammals, they only trigger weak gene silencing by targeting the CDS region but not the canonical miRNA target region UTR in mammals; and the existence of physiological and biochemical barriers in mammals could strongly ensure the non-target safety of pcamiR1 as a fungicide. However, the safety issue still should be further evaluated as long as pcamiR1 analogues were applied comply with the safe crop period.

## Conclusion

**Fig 10:**
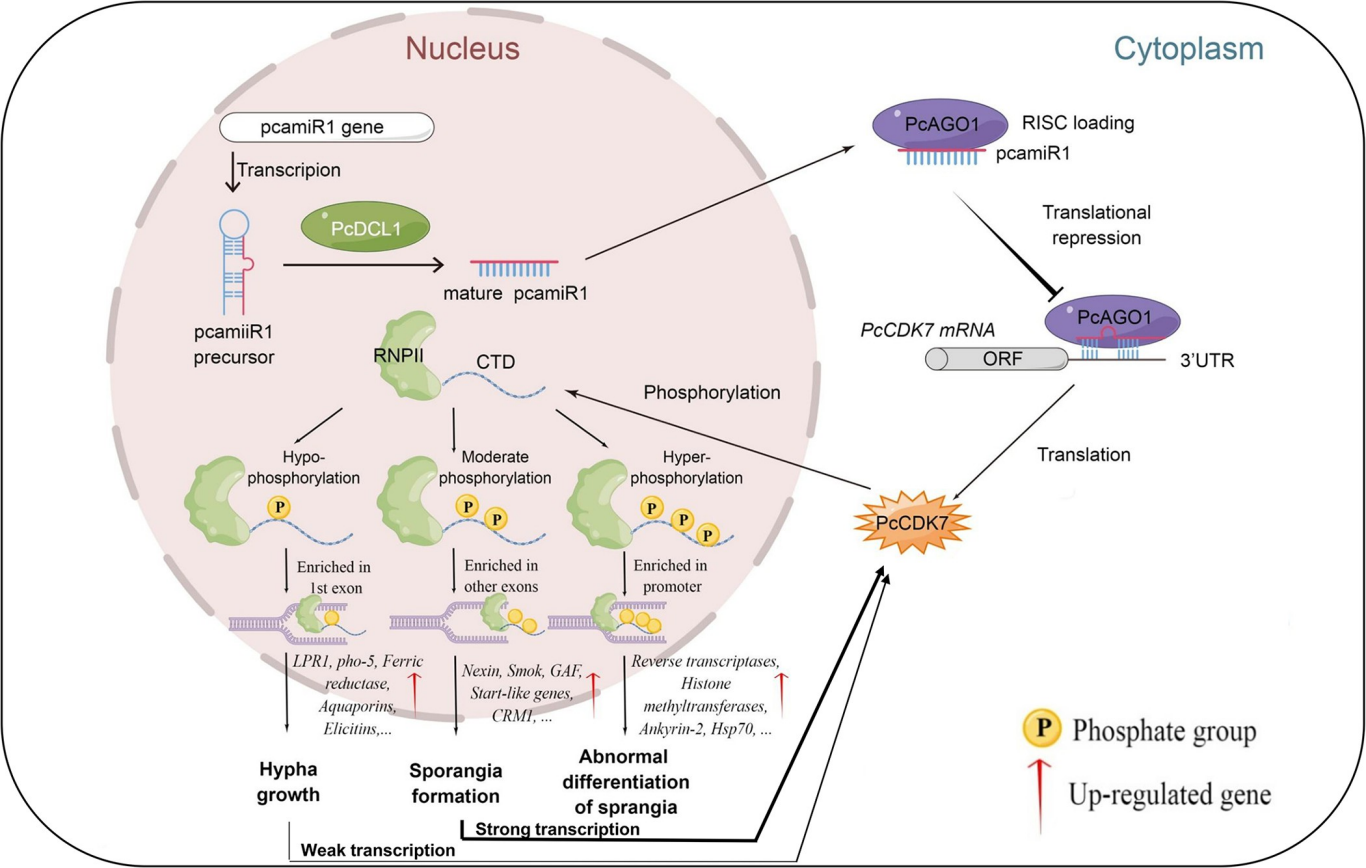
The model of RNPII phosphorylation modulation to ensure proper transcription and development. Sporangia are indispensable for the development and pathogenesis of *P*. *capsici*. *P*. *capsici* produces sporangia in medium or infected plants after the vegetative growth of hyphae. During the transition from hypha growth to sporangia formation, the transcriptional profile changes dramatically, largely depending on the phosphorylation states of RNPII. During this morphological transition, the cyclin-dependent kinase PcCDK7 is highly induced and promotes the phosphorylation of Ser5 in the characteristic YSPTSPA repeats located in RNPII CTD. As a feedback regulatory machinery, the novel DCL1-dependent microRNA pcamiR1 is also upregulated in line with *PcCDK7* and further silences *PcCDK7* through complexing with PcAGO1, which ensures the moderate level of PcCDK7. The pcamiR1-PcCDK7 module precisely modulates the phosphorylation state of RNPII and ensures appropriate gene transcription. Therefore, sporangia formation can only be accomplished by accurate gene expression, and any disturbance of the balance between pcamiR1 and *PcCDK7* could result in an improper phosphorylation state of RNPII and subsequently lead to the impairment of the normal growth of *P*. *capsici*. Due to the conservation of this module in *Phytophthora* and animalia, pcamiR1 may be a promising target for disease control in the future. Figure created using Figdraw (www.figdraw.com).

In this study, it is demonstrated that the phosphorylation of the unique heptapeptide repeats in RNPII are regulated by a cell cycle kinase CDK7 in the transition from hypha growth to sporangia formation in *Phytophthora* in feedback. Specifically, the phosphorylation plasticity largely relies on an evolutionarily conserved and DCL1-dependent novel miRNA in both *Phytophthora* and mammals. Here also elucidated the fine-tuned transcriptional modulation involved in the expression of certain genes through regulation of the chromosomal occupation of RNPII via phosphorylation regulation (Fig 10). Considering that CDK7 dysregulation is correlated with various important diseases, the novel CDK7 regulator identified in this study, pcamiR1, or its analogue may represent an evolutionarily conserved target of new anti-tumor drugs. In brief, the novel miRNA-dependent module that ensures rapid and precise RNPII CTD phosphorylation further guarantees appropriate transcription and normal cellular development or pathogenesis. The discovery of the evolutionarily conserved and effective regulatory module could strongly benefit the control of both human and plant diseases.

## Material and methods

### *Phytophthora* cultivation and phenotypic analysis

*P*. *capsici* isolates (listed in Table D in S1 Text) were grown on V8 medium at 25°C in the dark. For sporangia quantification, the number of sporangia was calculated under a microscope per view (100× amplified). Values shown are the average (mean ± SD) of at least 10 views of each isolate. In consideration of the different hypha growth rates in different transformants, we performed the sporangia analysis after they had reached the same colony diameter in the V8 plate (for instance, 9 cm) to eliminate differences induced by the growth rate. Other phenotypic assays were performed according to Wang el al. [24]. *P*. *infestans* isolate MZ and *P*. *sojae* isolate P6497 were cultured on V8 agar medium at 18°C or 25°C, respectively.

### Gene analysis

*Phytophthora* gene sequences were retrieved from the DOE Joint Genome Institute (JGI, http://www.jgi.doe.gov/) database. Genes from other species were retrieved from the National Center for Biotechnology Information (NCBI) database. The information of these genes was listed in Table E in S1 Text. The amino acid sequences encoded by these genes were aligned with ClustalW2 (http://www.ebi.ac.uk/Tools/msa/clustalw2/) [64]. In addition, phylogenetic trees were constructed with the maximum likelihood evolution algorithm in MEGA 7.0 [65]. A Poisson correction was used for multiple substitution models and pairwise deletion was used for handling missing data. Statistical accuracy was assessed with 1,000 bootstrap replicates.

### Transformation of *P*. *capsici*

Primers for gene manipulation and mutant identification were designed and synthesized (listed in Table F in S1 Text). Gene knockout isolates were generated by the CRISPR-Cas9 system, while gene complement or overexpression and sRNA transformation were all performed by PEG-mediated protoplast transformation as previously described [66]. To complement the deletion mutants, the full-length *PcDCL1* or pcamiR1 precursor fragment with its native promoter sequence was inserted into *pTOR-OSBP1* and introduced into the protoplast of the koPcDCL1 or kopcamiR1 mutant, respectively. Overexpression was performed via a complement assay but the *HAM34* promoter was used instead of the native promoter, and *pTOR*::*HAM34*::*PcCDK7* or *pTOR*::*HAM34*::*pcamiR1* were transformed into LT1534 or oePcCDK7, respectively. For sRNA transformation, 25 μg of the *pTOR* plasmid DNA and 100 ng of miRNA antagomir or control miRNA were added into 1 mL of protoplast suspension (5 × 10^5^/mL) for transformation. The resulting G418-/oxathiapiprolin-resistant transformants were sub-cultured on V8 at 25°C in the dark and confirmed by PCR.

### THZ1 inhibitory assay

Ten milligrams of THZ1 (MCE, Shanghai, China) was dissolved in 1 mL DMSO to prepare the stock; the solution was then added to V8 agar medium at concentrations of 0, 0.1, 1, 5, and 20 μM. *P*. *capsici* strains were inoculated onto the plates and incubated at 25°C with a 12-h light/12-h dark cycle for 5 or 7 days (5 days for the wild type [WT] and 7 days for the mutants). Phenotypic analyses were performed according to Wang [24].

### GFP reporter assay

Plasmids harboring the GFP coding sequence followed by the 120 nt *PcCDK7*-3’-UTR sequence that comprised the potential pcamiR1 target site (CDK7ts) or a control sequence similar to CDK7ts but mutated the 36-nt target site with reshuffling sequence (ckts) were constructed using pEARLYGATE100 (pEG). They were transiently expressed in *N*. *benthamiana* after transforming into agrobacterium GV3101. PcamiR1 was co-expressed in the tobacco leaves with pEG-GFP-CDK7ts or pEG-GFP-ckts. MiRNA expression was performed by expressing an artificial miRNA precursor with substitutions in the mature miRNA sequence and its reverse-complementary sequence in the precursor of miR171 [67]. The GFP signal intensity and expression level were detected by confocal microscopy and western blotting, respectively. The four treatments were performed and compared in a single leaf to avoid disturbance caused by leaf state. Five replicates were included in each expreiment and the experiment was individually performed three times with similar results.

### RNA extraction and gene expression analysis

To assess the gene transcripts and pre-miRNA and mature miRNA expression profiles during various *P*. *capsici* developmental stages, hypha, sporulated hypha, sporangium, zoospore, and cyst samples were collected [68]. To measure the transcript levels during a *P*. *capsici* infection, each pepper (cv. Xichengdaniujiao) leaflet was inoculated with 20 μL LT1534 zoospore suspension (10^4^/mL) and then incubated at 25°C with a 12-h light/12-h dark cycle for 3 days. Leaves were collected at 0, 6, and 12 h as well as at 1, 2, and 3 days and immediately placed in liquid nitrogen. Total RNA was extracted and gene expression was analyzed as previously described [24]. For the mature miRNA expression assay, total RNA was reverse-transcribed using a miRcute Plus miRNA First-Strand cDNA Kit (KR211, TIANGEN, Beijing, China) and quantified with a miRcute Plus miRNA qPCR Kit (FP411, TIANGEN, Beijing, China) according to the manufacturer’s protocols. *WS21* and *Ubc* were used as internal controls for gene and pre-miRNA measurement, and *5S rRNA* was used for mature miRNA measurement [69]. Three replicates were used for each treatment and the experiment was repeated three times.

### sRNA-seq and mRNA-seq

The small RNA libraries were prepared with a TruSeq small RNA library prep kit (Illumina, San Diego, USA). Small RNA library size selections were performed using the BluePippin System (SAGE Science, Shanghai, China). Illumina sequencing was performed by BGI Genomics (Shenzhen, China) with a HiSeq4000 platform. Small RNA reads were checked by FastQC (version 0.11.4, https://www.bioinformatics.babraham.ac.uk/projects/fastqc/) and MultiQC [70], then mapped with Bowtie (version 1.1.2) [71] to rRNA, tRNA, snoRNA, and snRNA from RFAM (version 14.1) [72]. Unmapped reads were then mapped, also with Bowtie, to the databases for precursor and mature miRNAs for all species from miRBase (release 21). The remaining reads were analyzed by stem-loop structure prediction using Mirdeep and Mireap. Target prediction was performed by RNAhybrid and Miranda, the genes simultaneously screened by the two tools were treated as potential target genes of miRNAs.

mRNA libraries were prepared with an in-house, scaled-down version of Illumina’s Tru-seq reaction. Paired-end Illumina sequencing was performed by Gene Denovo (Guangzhou, China) with HiSeq4000 or Illumina Novaseq6000 platforms. Reads were mapped to the *P*. *capsici* genome with STAR (version 2.5.2b) [73] and gene counts were obtained with featureCounts (version 1.6.2). Finally, differentially expressed genes were identified using DESeq2 (version 1.20.0) within the R statistical programming environment (R Core Team, 2018), filtering genes with 10 or less counts in all samples. Two biological replicates of each sample were prepared and sequenced by sRNA-seq and RNA-seq, the overlap genes or sRNAs with the same shifty trend in the two biological replicates were further analyzed.

### Northern blotting

Digoxin-labeled DNA oligonucleotides were synthesized by BGI Write (Beijing, China). Northern blots were performed as previously described [74]. Briefly, 10 μg of total RNA was resolved in 17% (v/v) polyacrylamide gels under denaturing conditions (7 M urea) and then transferred to HyBond-N^+^ charged nylon membranes (Amersham, Beijing, China) by semidry electroblotting. RNA was covalently fixed to the membranes by EDC crosslinking. Membranes were hybridized overnight with DIG-labeled DNA oligonucleotides. The signal was detected using CSPD ready-to-use solution (Roche, Shanghai, China).

### Protein extraction and western blotting

Protein was extracted from 50 mg ground tissue using a Minute total protein extraction kit for microbes with thick cell walls (Invent Biotechnologies, Beijing, China) according to the manufacturer’s protocol. Total protein levels were quantified using a BCA protein assay kit (Beyotime, Shanghai, China). The standard western blotting procedure was followed [75]. Anti-RNPII (to detect unphosphorylated RNPII) and anti-Ser5 phosphorylated RNPII (both from SantaCruz, Beijing, China), anti-β-tubulin, anti-3×FLAG, anti-GST and anti-6×His (all from Proteintech, Wuhan, China) were used to detect proteins. The following secondary antibodies were used: goat anti-mouse HRP-conjugated and goat anti-rabbit HRP-conjugated antibody (both from CWBIO, Beijing, China). After antibody incubation, the membranes were developed with Femto ECL substrate (CWBIO, Beijing, China) and imaged using a CCD camera. The intensity of the bands was measured with ImageJ and normalized to β-tubulin.

### Co-immunoprecipitation

After protein extraction, immunoprecipitation was performed using 3×FLAG magnetic beads (Sigma, Shanghai, China) following Gu [76]. Proteins were eluted from the beads with 50 μL of 2× SDS loading buffer (without DTT) and detected by SDS-PAGE after 100 mM DTT was added.

### GST pull-down assay

Full length 1323-nt PcCTD, single amino acid-mutated CTD^S2A^/CTD^S5A^/ CTD^T4A^, and the truncated protein CTD^LLC^ (in which the typical YSPTSPS sequence located in the last of the heptapeptide repeats was deleted) were fused with a GST tag and expressed in *E*. *coli* BL21. The constructs were purified using glutathione-sepharose beads (GE Healthcare, Beijing, China). The 6×His fusion protein encoding PcCDK7 was expressed in *E*. *coli* and purified using Ni sepharose beads (Thermo, Shanghai, China). To test *in vitro* binding between GST- and His-tagged proteins, 3 μg of GST-tagged protein or GST (negative control) that was still bound to the glutathione beads was mixed with 10 μg of His-tagged protein and rocked for 2 h at 4°C. The beads were washed six times with cold TBS buffer and resuspended in 5× SDS sample buffer. GST pull-down proteins were then analyzed by SDS-PAGE.

### Subcellular localization detection of PcCDK7

PcCDK7 was labelled with GFP in its carboxyl end, the localization of PcCDK7 was examined under confocal microscopy, GFP fluorescence was excited at a wavelength of 488 nm. Moreover, protein was extracted from oePcCDK7 (0.5 g tissue of sporulated hypha), the nuclear fraction and cytoplasmic fraction were isolated according to a previous study [77]. The two fractions were adjusted to obtain equal concentrations, and the fractions (1 mg) were analysed by 10% SDS–PAGE and immunoblotted using a 3×FLAG antibody (Proteintech, Wuhan, China) for PcCDK7 detection, GAPDH (SantaCruz, Beijing, China, ~36 kDa) and Histone H3 (Abcam, Shanghai, China, ~17 kDa) antibodies were used as markers to characterized the cytosolic fraction and the nucleus, respectively.

### ChIP-seq

ChIP-seq assay were adapted from Abcam (https://www.abcam.com/). One gram of *P*. *capsici* hyphal tissue in V8 liquid media was crosslinked with formaldehyde. Enriched nuclei were sonicated in an ultrasonic cell disruptor (10 s on/10 s off pulses at high intensity for 60 cycles). Samples were incubated and immunoprecipitated with anti-Ser5 phosphorylated RNPII conjugated agarose beads (SantaCruz, Beijing, China) for 12 h at 4°C. Reverse crosslinking was performed with proteinase K (Transgene, Beijing, China). Immunoprecipitated DNA was recovered using a phenol:chloroform:isoamyl alcohol mix (25:24:1) followed by ethanol precipitation and sequenced by Gene Denovo (Guangzhou, China). Untreated sonicated chromatin was processed in parallel and considered the input sample. ChIP DNA-end was repaired to overhang a 3’-dA, then adapters were ligated to the end DNA fragemnts. DNA fragments with 100–300 bp were selected after PCR amplification and subsequently used for sequencing. The bioinformatics analysis of ChIP-seq data was performed according to previous literatures [78]. Two replicates of each sample were sequenced, the common peaks appeared in both replicates with similar copy numbers were extracted and further analyzed.

### Ribosome/polysome fractionation and quantitation of *PcCDK7* mRNA

One gram of *P*. *capsici* sporulated hypha tissues from LT1534 or kopcamiR1 isolates were collected. Cytoplasmic lysate preparation, ribosome/polysome fractionation, and RNA extraction from sucrose gradient harboring different states of ribosome were performed according to Panda et al. [79]. RT-qPCR was performed to quantify the *PcCDK7* mRNA and the housekeeping gene *WS21* mRNA in total RNA, none-ribosome, 40–80S ribosome, and polysome fractions, the relative abundance was calculated as previously described [24].

### PcAGO expression, *in vitro* pcamiR1-AGO1 assembly, and translation inhibition assays

3×FLAG-tagged *PcAGOs* were amplified using the primers listed in Table F in S1 Text and inserted into a pF3A WG (BYDV) Flexivector. The four proteins were expressed using the TNT SP6 high-yield wheat germ protein expression system (WGE system) following the manufacturer’s protocol (Promega, Beijing, China).

*In vitro* pcamiR1-AGO1 assembly and translation inhibition assays were also performed with the WGE system, with slight modifications. PcamiR1 mimics (100 nM, 5’ phosphonate) and 20 μL PcAGO1/3/4/5 were added to 100 μL reaction buffer (25 mM HEPES-KOH [pH 7.5], 5 mM MgCl_2_, 50 mM KCl, 5 mM DTT, 0.2 mM EDTA, 0.05 mg/mL BSA, and 5 U/μL RNase inhibitor), then incubated at 37°C for 1 h. Subsequently, the reaction mixture was incubated with anti-Flag M2 beads (Sigma-Aldrich, Shanghai, China) on a rotator at 4°C for 1 h. Then, the beads were washed three times with lysis buffer (30 mM HEPES [pH 7.4], 100 mM KOAc and 2 mM Mg(OAc)_2_) containing 1% Triton X-100 and 800 mM NaCl. After treatment with proteinase K, the samples were mixed with an equal volume of 2× formamide dye, incubated for 5 min at 68°C, and loaded onto a 15% urea PAGE denaturing gel. After drying, the gel contents were transferred onto a membrane and detected by northern blotting using a probe DIG-pcamiR1. For the translation inhibition assay, RISCs contain pcamiR1 or miR8788 mimics (5’ phosphonate) and PcAGO1 or PcAGO4 were assembled as previously described, respectively. One microliter RISC (the amounts were varied in the RISC gradient assay) was added to the expression system containing pF3A::GFP-CDK7ts or pF3A::GFP-ckts (the same with GFP reporter interaction assay). The translated proteins were then detected by western blotting after incubation for 3 h at 25°C.

### *In vitro* phosphorylation assay

His-tagged PcCDK7 and its substrate peptide RNPII CTD were expressed and purified as described above. Fifty millimolar Tris-HCl, 5 mM DTT, 500 μM ATP and AMP, 5 μM RNPII CTD proteins, and 0.8 pM CDK7 were mixed and incubated at 30°C for 10 min. Then, SDS loading buffer was added and boiled for 5 min to terminate the reaction. The phosphorylation state of RNPII CTD was detected using an anti-pan phosphoserine antibody (Proteintech, Wuhan, China) by western blotting.

### Enrichment and sequencing of phosphorylated proteins in *P*. *capsici*

Enrichment of phosphorylated proteins was performed using the Qiagen PhosphoProtein Purification System (Shanghai, China) according to the manufacturer’s protocol. The enriched proteins were hydrolyzed by trypsin and the tryptic peptides were sequenced by tandem mass spectrometry (MS/MS) in Q Exactive Plus (Thermo) coupled online to an EASY-nLC 1000 UPLC system (PTM Bio, Hangzhou, China). The resulting MS/MS data were processed using Maxquant search engine (v.1.5.2.8). The mass tolerance for precursor ions was set as 20 ppm in First search and 5 ppm in Main search, and the mass tolerance for fragment ions was set as 0.02 Da. FDR was adjusted to < 1% and minimum score for modified peptides was set > 40. Samples were collected from 10 replicates of each isolate, proteins and modification sites were filtered with localization probability > 0.75 and threshold value of expression fold change > 2 or < 0.5 (WT/koPcDCL1).

### Human cell cultures and miRNA transfection

The human 293T cell line was acquired from Jishishengming (Beijing, China) and grown in a humidified atmosphere at 37°C with 5% CO_2_ using MEM medium (Gibco, Shanghai, China) supplemented with 10% fetal bovine serum (Corning, Shanghai, China) and 1% penicillin and streptomycin antibiotics (Gibco, Shanghai, China). The culture medium was replaced every other day. PcamiR1, its analogue miRNA (has-mir-365a-5p), and a control miRNA were designed and supplied by Jishishengming (Beijing, China). Their sequences are listed in Table F in S1 Text. The SiLentFect lipid reagent (Bio-Rad, California, USA) was used for transfection, and the experiments were performed as per Vinther [80]. Protein was extracted from the water-treated cells or transfected cells after 48 h and detected by western-blotting.

### Statistical analysis

Details for specific statistical tests are found in the legends associated with each figure. The data is stored in Dryad database (https://doi.org/10.5061/dryad.k3j9kd5gk) [81]. In brief, data represent an average of at least three biological replicates, each with three technical replicates. Bars indicate mean ± SD. The unpaired t test or analysis of variance with multiple comparisons test was conducted using Graphpad Prism v. 8.0.2 software to determine significant differences between groups. The P values represented in each figure are shown in the figure legends.

## Supporting information

S1 Text Tables A-F and Figs A-N. (DOCX)

S1 Table The potential target genes of pcamiR1. (XLSX)

S1 Data The underlying numerical data and statistical analysis for Figs 1F, 2B, 2C, 2G, 3C, 3D, 3E, 3G, 4B, 4C, 4E, 4H, 5C, 5D, 5F, 5H, 6G, 8A, 8B, 8C, 8D, 9A, 9B and 9F. (XLSX)
