# Supplementary material for: A novel and ubiquitous miRNA-involved regulatory module ensures precise phosphorylation of RNA polymerase II and proper transcription
Source: PLoS Pathog. 2024 Apr 19;20(4):e1012138. doi: 10.1371/journal.ppat.1012138 (PMC11062530; doi:10.1371/journal.ppat.1012138)
Supplement: S1 Text — (DOCX) [file ppat.1012138.s001.docx]

**Supplementary information**

Table A. Information on the genes with altered expression levels in RNA-seq and shifty binding abundance in ChIP-seq in the moderately phosphorylated RNPII vs hypo-phosphorylated RNPII group.

Table B. Information on the genes with altered expression levels in RNA-seq and shifty binding abundance in ChIP-seq in the hyper-phosphorylated RNPII vs moderately phosphorylated RNPII group.

Table C. Ct value of pcamiR1 detected in *Fusarium graminearum*, *Botrytis cinerea* and *Magnaporthe grisea*

Table D. *Phytophthora capsici* isolates generated and used in this study

Table E. *Phytophthora capsici* and *Homo sapiens* genes list

Table F. Primers and probes used in this study

Fig A. RNA-seq assays show distinct transcriptome patterns during sporangium development in *Phytophthora capsici*.

Fig B. PcDCL2 is not responsible for the generation of the canonical *Phytophthora* microRNA miR8788.

Fig C. Construction and identification of *PcDCL1* deletion and complemented mutants.

Fig D. PcDCL1 is involved in hyphal growth, zoospores production, oospores production and virulence in *Phytophthora capsici*.

Fig E. Small RNA-seq assay show distinct small RNA profiles between hypha stage (HY) and sporangium stage (SP) in *Phytophthora capsici*.

Fig F. Identification of pcamiR1 in wild-type LT1534 and koPcDCL1 mutants (A) and pcamiR1-expressing *Nicotiana benthamiana* plants (B) by northern blotting.

Fig G. Construction and identification of pcamiR1 deletion and complemented mutants.

Fig H. pcamiR1 is involved in hyphal growth, zoospores production and virulence in *Phytophthora capsici*.

Fig I. Construction and identification of *PcCDK7* overexpression in LT1534 and pcamiR1 overexpression in oePcCDK7.

Fig J. Expression and purification of PcCDK7 and CTD (carboxyl terminal domain of RNPII largest subunit 1) and its mutated proteins.

Fig K. miR8788-AGO1 could not inhibit target gene translation regardless of its concentration.

Fig L. Transcriptomic comparisin of wild-type LT1534 and kopcamiR1 in hypha (HY) and sporulated hypha (HYSP) stages.

Fig M. The transcriptome differences happened between wild-type *Phytophthora capsici* and the important regulator pcamiR1 deficient mutant during sporangia development.

Fig N. Gene ontology (GO) analysis of the positively related genes both significantly changed in ChIP-seq and RNA-seq.

**Table A. Information on the genes with altered expression levels in RNA-seq and shifty binding abundance in ChIP-seq in the moderately phosphorylated RNPII vs hypo-phosphorylated RNPII group.**

| Gene ID | Change in RNA-seq^a^ | Peak | Change in ChIP-seq^b^ | Annotation | Function description | Classification | Reference |
| --- | --- | --- | --- | --- | --- | --- | --- |
| **upregulated genes when RNPII is moderately phosphorylated compared to hypophosphorylated state** | | | | | | | |
| 545125 | 3.59504 | scaffold17: 197212-199191; 196549-196801 | 1.62177; 1.26497 | Sorting nexin-1 | Membrane coat complex Retromer; intracellular trafficking, secretion, and vesicular transport. | Cell components | (1) |
| 541558 | 3.82908 | scaffold7: 483892-484184 | 1.57959 | Smok, Serine/threonine protein kinase | Components of a signal cascade which control sperm motility in animals. | Cell signaling | (2) |
| 533673 | 13.37857 | scaffold16: 457377-457640 | 0.88578 | GAF domain-like protein | Involved in signal transduction pathways and protein regulatory/sensory systems, including gas sensor. | Cell signaling | (3) |
| 41912 | 12.45121 | scaffold74: 176467-176701; 177312-177568 | 1.48439; 1.46814 | START-like domain protein | Intracellular lipid and sterol transport; transcriptional regulation. | Transportation | (4, 5) |
| 575529 | 4.83631 | scaffold74: 178097-178416 | 1.34422 | START-like domain protein |  |  |  |
| 537120 | 16.96776 | scaffold74: 101919-102229 | 1.28971 | START-like domain protein |  |  |  |
| 511072 | 6.33599 | scaffold74: 94340-94761 | 0.98736 | START-like domain protein |  |  |  |
| 129734 | 14.05070 | scaffold86: 155504-156078 | 1.16059 | CRM1 C terminal Exportin 1 protein | A major receptor for the export of proteins/RNAs out of the nucleus; involved in various steps during mitosis. | Transportation | (6) |
| 128239 | 3.39474 | scaffold74: 114899-115235 | 1.02015 | Histidine phosphatase superfamily | Membrane recruitment of cytosolic proteins by binding to PI3P. Involved in metabolic regulations and development. | Transportation | (7) |
| 539488 | 2.92616 | scaffold3: 954167-961172 | 1.03396 | zinc finger (ccch type) motif-containing protein | Regulatory functions at all stages of mRNA metabolism. | Nucleic acid binding | (8) |
| 131247 | 15.25008 | scaffold103: 8480-9535 | 0.55891 | 12-oxophytodienoate reductase 1 | Involved in the biosynthesis of the plant hormone jasmonic acid. | Metabolism | (9, 10) |
| 560843 | 3.98588 | scaffold5: 1002271-1006890 | 1.20358 | Uncharacterized protein | Unknown. | Unclear |  |
| 96751 | 12.64611 | scaffold1: 403989-405616 | 0.92407 | Uncharacterized protein | Unknown. | Unclear |  |
| **downregulated genes when RNPII is moderately phosphorylated compared to hypophosphorylated state** | | | | | | | |
| 64839 | -7.63336 | scaffold17: 759206-762054 | -0.98801 | LPR1, Spore coat protein A | Spore coat assembly; manganese catalases, peroxiredoxin and chitinase. | Cell component | (11, 12) |
| 507010 | -1.18735 | scaffold24: 3727-9592 | -1.53307 | Carbohydrate-binding protein | Deciphering complex structural pattern of glycans, involved in embryogenesis, immune response, protein trafficking. | Cell signaling | (13) |
| 502829 | -2.82676 | scaffold1: 1775198-1776078 | -1.46938 | pho-5, Major facilitator superfamily protein | Transport of simple sugars, oligosaccharides, inositols, drugs, amino acids, nucleosides, organophosphate esters, Krebs cycle metabolites, anions, cations, and toxins. | Transportation | (14) |
| 118653 | -3.56296 | scaffold36: 187114-189083 | -1.45949 | Ferric reductase | Involved in a reduction step in cellular iron absorption. | Transportation | (15, 16) |
| 506701 | -3.64956 | scaffold21: 342019-343725 | -1.21565 | fcyB, Purine-cytosine permease | cytosine scavenger and purine uptake. | Transportation | (17) |
| 511729 | -9.80429 | scaffold96: 27519-29196 | -1.21065 | AQP10, Aquaporin | Transportation of water, small neutral solutes (urea, boric acid, and silicic acid) and gases (ammonia and carbon dioxide). | Transportation | (18) |
| 511725 | -6.04655 | scaffold96: 17897-18252 | -0.94012 | AQP3, Aquaporin |  |  |  |
| 536142 | -5.44437 | scaffold48: 136999-141244 | -1.07418 | R000093, Amino acid/polyamine transporter family 1 | Transporting amino acids, polyamines, nucleotides, peptides, and inorganic anions or cations across the plasma membrane, including amino acid. | Transportation | (19) |
| 509954 | -9.37950 | scaffold52: 117220-129185 | -1.60062 | Zinc finger protein, RING/FYVE/PHD-type | DNA binding and zinc ion binding; regulation of transcription | Nucleic acid binding | (20) |
| 508441 | -1.82532 | scaffold35: 135814-137930 | -1.28396 | Gba, Beta-glucosidase/xylosidase | Cellulose, specific for substrates with a beta-(1,4)-glucosidic linkages, completes the final step during cellulose hydrolysis by converting the cellobiose to glucose. | Metabolism | (21) |
| 508057 | -15.12181 | scaffold32: 121100-123930 | -0.55936 | HINT4, Histidine triad nucleotide-binding protein | Adenylate and phosphoramidate hydrolases; intracellular receptors for purine mononucleotides | Metabolism;  Cell signaling | (22) |
| 508763 | -1.00935 | scaffold38: 58210-60575 | -1.56411 | B20, Elicitin | Elicit a hypersensitive response -like necrosis and systemic acquired resistance in a restricted number of plants. | Virulence | (23) |
| 570597 | -4.65010 | scaffold38: 56971-57845 | -1.00480 | B26, Elicitin |  |  |  |
| 529073 | -1.46073 | scaffold38: 48037-51978 | -1.33368 | B20, Elicitin |  |  |  |
| 503750 | -4.09213 | scaffold4: 1060093-1060417 | -1.43004 | EO, Reticulon-4-interacting protein 1 | A mitochondrial protein that reduces the anti-apoptotic activity, involved in axonal regeneration by promoting dendrite outgrowth on neurons. | Unclear |  |

^a^ Change in gene expression level was determined by log2(fold change), fold change = mean fpkm in moderate phosphorylation/mean fpkm in hypophosphorylation.

^b^ Change in DNA binding abundance was determined by log2(read density ratio), read density ratio = mean read density in moderate phosphorylation/mean read density in hypophosphorylation.

**Table B. Information on the genes with altered expression levels in RNA-seq and shifty binding abundance in ChIP-seq in the hyper-phosphorylated RNPII vs moderately phosphorylated RNPII group.**

| Gene ID | Change in RNA-seq^a^ | Peak | Change in ChIP-seq^b^ | Annotation | Function description | Classification | Reference |
| --- | --- | --- | --- | --- | --- | --- | --- |
| **upregulated genes when RNPII is hyperphosphorylated compared to moderately phosphorylated state** | | | | | | | |
| 50676 | 15.19749 | scaffold75: 253558-254032 | 4.03881 | Retrotransposon gag domain protein | Chromosomal rearrangements. | Epigenetic regulation | (24) |
| 123614 | 4.98703 | scaffold51: 81997-82244 | 3.9326 | reverse transcriptase | Conversion of RNA to DNA; required for the replication of transposable elements. | Epigenetic regulation | (25) |
| 44449 | 7.33021 | scaffold51: 80101-81010 | 2.8097 | reverse transcriptase |  |  |  |
| 22069 | 13.44800 | scaffold580: 3039-3260 | 2.7188 | reverse transcriptase |  |  |  |
| 70901 | 18.15795 | scaffold15: 625179-629591 | 3.51139 | Histone-lysine N-methyltransferase | Methylating lysine residues in histone proteins, involved in heterochromatin formation, X-chromosome inactivation, and transcription regulation. | Epigenetic regulation | (26) |
| 47625 | 1.63103 | scaffold75: 249178-250068 | 2.62346 | Histone H3-K79 methyltransferase | Involved in telomere silencing, meiotic checkpoint control, and mitotic cell cycle. | Epigenetic regulation | (27) |
| 80550 | 14.84700 | scaffold5: 1580215-1581404 | 2.20420 | RE2, Integrase catalytic core protein | Recombinases that catalyze intermolecular DNA rearrangements without energetic input. | Epigenetic regulation | (28) |
| 50912 | 13.13971 | scaffold75: 197286-199236 | 2.84784 | Ankyrin-2 | Link the spectrin/actin cytoskeleton to the cytoplasmic domain of the anion exchanger; cellular organization of the sodium pump, the sodium/calcium exchanger, and inositol-1,4,5-trisphosphate receptors; Ca^2+^ signaling. | Cell component; Transportation; Cell signaling | (29, 30) |
| 73036 | 17.89253 | scaffold15: 1029038-1029299; 1028109-1028820 | 4.17124;  4.11326 | hsp70-like protein | Activates MAPK and NF-κB pathways; promote dendritic cell maturation and activation. | Cell signaling | (31) |
| 102562 | 15.53776 | scaffold7: 435699-436163; 436762-437170 | 1.31090; 1.30935 | Dual specificity protein phosphatase 7 | Involved in multiple signaling pathways including MAP kinase signaling; cell cycle regulation. | Cell signaling | (32) |
| 507193 | 3.26761 | scaffold25: 794717-795498 | 2.64635 | Winged helix-turn-helix DNA-binding domain | Transcriptional regulation. | Nucleic acid binding | (33) |
| 125689 | 14.01585 | scaffold59: 4376-4918 | 1.34994 | Zinc finger, CCHC-type | RNA transcription, polyadenylation, splicing, RNA export, translation, degradation, microRNA and ribosomal RNA biogenesis, and gene silencing. | Nucleic acid binding | (34) |
| 126428 | 14.97813 | scaffold63: 200385-200629 | 1.22768 | Methionine aminopeptidase | Removal of the N-terminal methionine during protein synthesis in eukaryotes; cell growth control. | Metabolism | (35, 36) |
| 20884 | 10.00973 | scaffold75: 204321-206117 | 4.54305 | Uncharacterized protein | Unknown. | Unclear |  |
| 100562 | 11.66622 | scaffold5: 596287-598517 | 2.55712 | Uncharacterized protein | Unknown. | Unclear |  |
| 18735 | 15.12787 | scaffold40: 166745-167043 | 1.53619 | Uncharacterized protein | Unknown. | Unclear |  |
| **downregulated genes when RNPII is hyperphosphorylated compared to moderately phosphorylated state** | | | | | | | |
| 56587 | -10.86110 | scaffold55: 307751-308042 | -7.39108 | Nucleolar GTPase/ATPase p130 | GTPase/ATPase activities; involved in nucleolar disassembly and reassembly. | Cell component | (37) |
| 123971 | -12.57460 | scaffold52: 100655-100950 | -1.10234 | mucin-21 | Has antiadhesive property and antiapoptotic activity; acts as epiglycanin. | Cell component | (38) |
| 11736 | -14.56000 | scaffold80: 65549-66063; 66318-66840 | -1.24999; -1.19833 | Pre-mRNA splicing factor SYF1 | Pre-mRNA splicing and cell cycle progression. | Nucleic acid binding | (39) |
| 553345 | -14.56240 | scaffold52: 359816-365911 | -1.65615 | PLAC8 family protein | Transport of heavy metals (cadmium or zinc), determination of organ size and growth, response to infection, Cd resistance. | Transportation | (40) |
| 129734 | -14.05100 | scaffold86: 155504-156078 | -1.02494 | CRM1 C terminal Exportin 1 protein | A major receptor for the export of proteins/RNAs out of the nucleus; involved in various steps during mitosis. | Transportation | (6) |
| 504089 | -15.28360 | scaffold6: 74666-75840 | -2.09592 | Kinesin protein | Has adenosine triphosphatase activity and microtubule-dependent plus-end motion ability. Involved in mitosis, meiosis and the transport of macromolecules. | Metabolism; Transportation | (41) |
| 96751 | -12.64610 | scaffold1: 403989-405616 | -1.59168 | Uncharacterized protein | Unknown. | Unclear |  |

^a^ Change in gene expression level was determined by log2(fold change), fold change = mean fpkm in hyperphosphorylation/mean fpkm in moderate phosphorylation.

^b^ Change in DNA binding abundance was determined by log2(read density ratio), read density ratio = mean read density in hyperphosphorylation/mean read density in moderate phosphorylation.

**Table C. Ct value of pcamiR1 detected in *Fusarium graminearum*, *Botrytis cinerea* and** ***Magnaporthe grisea***^a^

| Species | Ct value of pcamiR1 | | |
| --- | --- | --- | --- |
| *Fusarium graminearum* | 38.93 | 39.15 | 38.47 |
| *Botrytis cinerea* | 40.07 | 40.15 | 39.91 |
| *Magnaporthe grisea* | 39.65 | 39.33 | 39.51 |

^a^ The target is absent in the sample when Ct value > 35.

**Table D. *Phytophthora capsici* isolates generated and used in this study**

| *P. capsici* isolate | Description |
| --- | --- |
| LT1534 | Wild-type *P. capsici* |
| koPcDCL1 | *PcDCL1* knockout mutant generated from LT1534 background, geneticin resistant |
| comPcDCL1 | *PcDCL1* complementary isolate generated from koPcDCL1 background, geneticin and oxathiapiprolin resistant |
| kopcamiR1 | pcamiR1 knockout mutant generated from LT1534 background, geneticin resistant |
| compcamiR1 | pcamiR1 complementary isolate generated from kopcamiR1 background, geneticin and oxathiapiprolin resistant |
| oePcCDK7 | *PcCDK7* overexpression isolate generated from LT1534 background, geneticin resistant |
| oePcCDK7/pcamiR1 | pcamiR1 overexpression isolate generated from oePcCDK7 background, geneticin and oxathiapiprolin resistant |

**Table E. *Phytophthora capsici* and *Homo sapiens* genes list**

| Gene | ID | Database |
| --- | --- | --- |
| *PcDCL1* | 511433 | JGI |
| pcamiR1 precursor | PHYCAscaffold_14:73272-73346 | JGI |
| *PcCDK7* | 547497 | JGI |
| *PcRNPII largest subunit 1* | 527499 | JGI |
| *PcAGO1* | 525087 | JGI |
| *PcAGO3* | 106472 | JGI |
| *PcAGO4* | 566737 | JGI |
| *PcAGO5* | 533987 | JGI |
| *HsaCDK7* | 1022 | NCBI |
| *HsaRNPII largest subunit 1* | 5430 | NCBI |

**Table F. Primers and probes used in this study**

| Name | Sequence (5’-3’) | Function |
| --- | --- | --- |
| koPcDCL1-5’f | CCCAAGCTTGCTCTGCTGACTCTACATT | homologous recombination vector (HRV) for *PcDCL1* knock-out (5’) |
| koPcDCL1-5’r | CCGGAATTCGGTGTTAGTGTTGCCAGTA |  |
| koPcDCL1-3’f | CGCGGATCCTGAGGATGGTAAGGTGAGAT | HRV for *PcDCL1* knock-out (3’) |
| koPcDCL1-3’r | TGCTCTAGAGGAGAACGGCATTGTGAA |  |
| sgPcDCL1f | CTAGCCTGCATCTGATGAGTCCGTGAGGACGAAACGAGTAAGCTCGTCATGCAGCGCAACAATGACGA | sgRNA for *PcDCL1* knock-out |
| sgPcDCL1r | AAACTCGTCATTGTTGCGCTGCATGACGAGCTTACTCGTTTCGTCCTCACGGACTCATCAGATGCAGG |  |
| RPL41_Pseq_F | CAAGCCTCACTTTCTGCTGACTG | sgRNA vector identification |
| comPcDCL1f | AAGAGCTCGCAGAAAATGCGT | complement of *PcDCL1* |
| comPcDCL1r | CCTAAGGGTTACCGAATTCTGCT |  |
| pTORf | TGCAAGGCGATTAAGTTGG | identification of vector for gene complement |
| pTORr | TTGCCACGAACTGGATTTC |  |
| idPcDCL1f-1 | TACTGGCAACACTAACACC | identification of *PcDCL1* knock-out mutants (primer1) |
| idPcDCL1r-1 | ATCTCACCTTACCATCCTCA |  |
| idPcDCL1f-2 | AGTAACAACATGCAGATGCCTC | identification of *PcDCL1* knock-out mutants (primer2) |
| NPT II-R | TCAGAAGAACTCGTCAAGAAG |  |
| kopcamiR1-5’f | GTTCATTGAGACTAACTGGTACAA | HRV for pcamiR1 knock-out (5’) |
| kopcamiR1-5’r | GTCCACTCCCAGAAGAAGGAAA |  |
| kopcamiR1-3’f | TAGTAAGCCGTATCACGGATCAA | HRV for pcamiR1 knock-out (3’) |
| kopcamiR1-3’r | CTGTTCATCTCCCAGCGCTT |  |
| sgpcamiR1f | CTAGCCACACCCTGATGAGTCCGTGAGGACGAAACGAGTAAGCTCGTCGGTGTGACCCTACCATTGAG | sgRNA for pcamiR1 knock-out |
| sgpcamiR1r | AAACCTCAATGGTAGGGTCACACCGACGAGCTTACTCGTTTCGTCCTCACGGACTCATCAGGGTGTGG |  |
| compcamiR1f | CCATTCTTTTCCAGGGTGC | complement of pcamiR1 |
| compcamiR1r | GCATCACGTCCTTCAGAACC |  |
| idpcamiR1f-1 | GCAGGTCTTCAATCGGTT | identification of pcamiR1 knock-out mutants (primer1) |
| idpcamiR1r-1 | TCAAGTCCAGTTCGCTACG |  |
| idpcamiR1f-2 | GCCACTTTCACCTCATTCA | identification of pcamiR1 knock-out mutants (primer2) |
| NPTR | ACCGTAAAGCACGAGGAA |  |
| NPTF | TCTCCTGTCATCTCACCTTG | identification of pcamiR1 knock-out mutants (primer3) |
| idpcamiR1r-3 | GCATCACGTCCTTCAGAA |  |
| idpcamiR1f-4 | CAAAAAGATCGTGGATCGC | identification of pcamiR1 complementary mutants (primer4) |
| idpcamiR1r-4 | CTGCCAAGGCTCATCTAGA |  |
| idCDK7inmiR1f | TGATATCTCTGATGGACAAAGG | identification of pcamiR1 overexpression in PcCDK7 |
| idCDK7inmiR1r | CAAGCAGATCCAAACTGGACA |  |
| oePcCDK7f | GGACTAGTATGGGTAGCGACGCGCGGAA | overexpression of *PcCDK7* or labelling PcCDK7 with GFP |
| oePcCDK7r | TGCTCTAGACTACATCAATCGTCGCCCTTTG |  |
| PcCDK7-GFPr | CATCAATCGTCGCCCTTTG |  |
| idoePcCDK7f | GGGTACCATTTAAATCCTAGG | identification of *PcCDK7* overexpression |
| idoePcCDK7r | AATCGTCGCCCTTTGATCA |  |
| gfpCDK7tsF | CCGGAATTCACCGAACACGTGAGTTA | expressing *GFP-CDK7ts* in *Nicotian a benthamiana* |
| gfpCDK7tsR | TGCTCTAGACTAACGTGCTCGACGGTT |  |
| CDK7ckts | ACCGAACACGTGAGTTAAATGCACAAGCGAAGGATGCGTGTTGCTTAACGGCAATAGCACACTGACTCCCAACTATGACGTATCATAATGCACGCGCTGCGTATAACCGTCGAGCACGTTAG | expressing *GFP-CDK7ckts* in *N. benthamiana* |
| oemiR1inNbF | ATGAGAGAGTCCCTTTGAGGCGGAAAGGGACTTCTGGGGCTTCTTACCTGACCACACACGTAGATATACATTATTCTCTCTAGATTAAGCCCCATAAGTCCCCTTCCGCCTCTCAGTACTCTCTCGT | expressing pcamiR1 in *N. benthamiana* |
| oemiR1inNbR | ACGAGAGAGTACTGAGAGGCGGAAGGGGACTTATGGGGCTTAATCTAGAGAGAATAATGTATATCTACGTGTGTGGTCAGGTAAGAAGCCCCAGAAGTCCCTTTCCGCCTCAAAGGGACTCTCTCAT |  |
| qPcDCL1f | ATGTCTTCATGGGTCCGTTAC | quantify the expression level of *PcDCL1* |
| qPcDCL1r | TCCTCATTACCCGCATAACT |  |
| qpcamiR1f | GGAAAGGGACTTCTGGGGCT | quantify the expression level of pcamiR1 |
| universal reverse primer | provided by TIANGEN FP411 KIT |  |
| qprepcamiR1f | TTAAATATGAGGCGGAAAGG | quantify the expression level of *pcamiR1 precursor* |
| qprepcamiR1r | GGCGCCCGAAGGCAGCA |  |
| qPcCDK7f | ATTGTGGATGCTCCTGAC | quantify the expression level of *PcCDK7* |
| qPcCDK7r | TTGCTATTGCCGTTAAGCA |  |
| qPcWS21f | GGAAAGAACAAACGCCTGAC | quantify the expression level of *WS21* |
| qPcWS21r | GTTGCGCTCCGAGAAGATA |  |
| qPcUbcf | GAAGCGGATCAACAAAGAGC | quantify the expression level of *Ubc* |
| qPcUbcr | AAGCAGTGAGCAGATCGACA |  |
| qPc5SrRNAf | CAGTAAGAATGGCTGGACGATGGA | quantify the expression level of *5S rRNA* |
| qPc5SrRNAr | TTGCTCTCGATGTCTTGCTGTGT |  |
| DIG-pcamiR1 | 5’DIG-AGCCCCAGAAGTCCCTTTCCGCC-3’DIG | Norther-blot probe to detect pcamiR1 |
| DIG-Pc5.8SrRNA | 5’DIG-GCTTAACTTCACAGAGCAGAC-3’DIG | Norther-blot probe to detect *5S rRNA* |
| DIG-NbU6 | 5’DIG-CGATTTGTGCGTGTCATCCTTG-3’DIG | Norther-blot probe to detect *NbU6* |
| expPcCTDf | TTACGGCGAATACGCAGGCGT | expressing *PcCTD* and its mutated proteins in *Escherichia coli* |
| expPcCTDr | TTACGGTGCATACGCAGGCGT |  |
| expPcCTDLLCf1 | ATGCGTTATTCTTTCGATGGTGCAT | expressing *PcCTD-LLC* in *E. coli* |
| expPcCTDLLCr1 | TGGGGAAGCAGGGCTGTACGCAGGAGAGGTTGGCGAATAA |  |
| expPcCTDLLCf2 | TACAGCCCTGCTTCCCCA |  |
| expPcCTDLLCr2 | TTACTTGGAGTCCTCTTCTTC |  |
| expPcCDK7f | ATGGGTAGCGACGCGCGGAA | expressing *PcCDK7* in *E. coli* |
| expPcCDK7r | CTACATCAATCGTCGCCCTTTG |  |
| expAGO1f | GTTGCGATCGCATGCATCATCATCATCATCACATGCCAGGAAGGCGCAAC | expressing *PcAGO1* by WGE system |
| expAGO1r | AGCTTTGTTTAAACCTACACGAAGTACATCGCACC |  |
| expAGO3f | GTTGCGATCGCATGCATCATCATCATCATCACATGGATGCAGGCTTAAGACA | expressing *PcAGO3* by WGE system |
| expAGO3r | AGCTTTGTTTAAACTCAGATGAAATACATCGAGTCCT |  |
| expAGO4f | GTTGCGATCGCATGCATCATCATCATCATCACATGGATCCTCGCGTGCAG | expressing *PcAGO4* by WGE system |
| expAGO4r | AGCTTTGTTTAAACTTACACGAAGAACATGCAATCAAG |  |
| expAGO5f | GTTGCGATCGCATGCATCATCATCATCATCACATGAGCTACAGCGGGG | expressing *PcAGO*5 by WGE system |
| expAGO5r | AGCTTTGTTTAAACTTAGATGAAAAACATGCAGTTTTTG |  |
| gfpF | GTTGCGATCGCATGGGCAAGGGCGAGGAA | expressing *GFP-CDK7ts* and *GFP-ckts* by WGE system |
| gfpCDKtsR | AGCTTTGTTTAAACCTAACGTGCTCGACGGTTATAC |  |
| gfpCDKcktsR | CTAACGTGCTCGACGGTTATACGCAGCGCGTGCATTATGATACGTCATAGTTGGGAGTCAGTG |  |
| mimicpcamiR1 | GGCGGAAAGGGACUUCUGGGGCU | pcamiR1 mimics, 5’ phosphonate |
| mimicmir365a | AGGGACUUUUGGGGGCAGAUGUG | has-mir-365a-5p mimics, 5’ phosphonate |
| mimicCKmir | GUGUCUAGGAGGAGGCGGUAGCU | control miRNA for human cell assay, 5’ phosphonate |
| mimicmiR8788 | CUGGUUCGCUUGGUACGCUAA | miR8788 mimics, 5’ phosphonate |
| antamiR1 | AGCCCCAGAAGUCCCUUUCCGCC | pcamiR1 antagomir |
| antamiRck | GCAACAGGCCCCUCCGCUUAUCC | pcamiR1 antagomir control |
| GFPf  GFPr | ATGGTGAGCAAGGGCGAG  TTACTTGTACAGCTCGTC | labelling GFP in the carboxyl terminal of PcCDK7 |


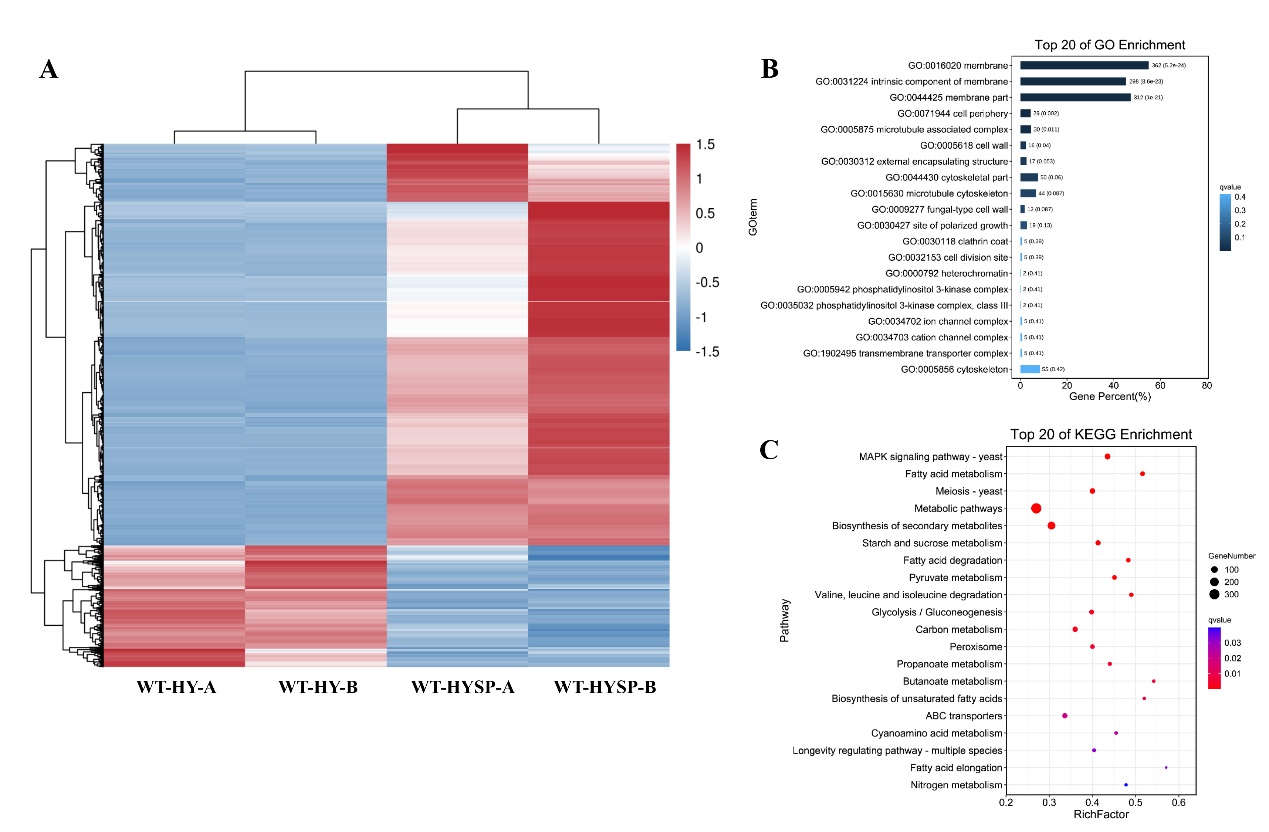


**Fig A. RNA-seq assays show distinct transcriptome patterns during sporangium development in *Phytophthora capsici*.** (A) Heat map showing the differentially expressed genes (DEGs) before or after sporangia emerge using RNA-seq. *P. capsici* LT1534 samples of hypha-only (HY) or hypha with plenty of sporangia (HYSP) were collected at 48 h or 72 h growing in liquid V8 medium to represent the morphological transition, respectively. Red color indicates relatively high expression, and blue indicates relatively low expression. (B) Gene ontology (GO) analysis of the DEGs during sporangium development. (C) KEGG (Kyoto Encyclopedia of Genes and Genomes) analysis of the DEGs during sporangium development.


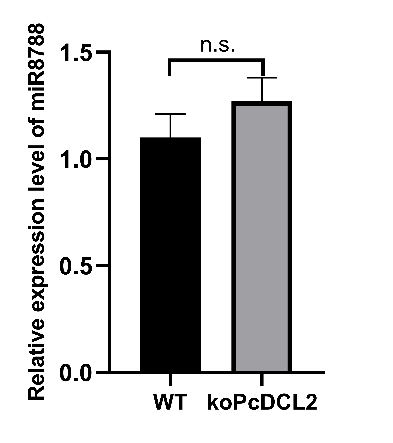


**Fig B. PcDCL2 is not responsible for the generation of the canonical *Phytophthora* microRNA miR8788.** Data presented are the mean ± standard deviation from three biological replicates. Data was analyzed according to the t-test (*p* < 0.05).


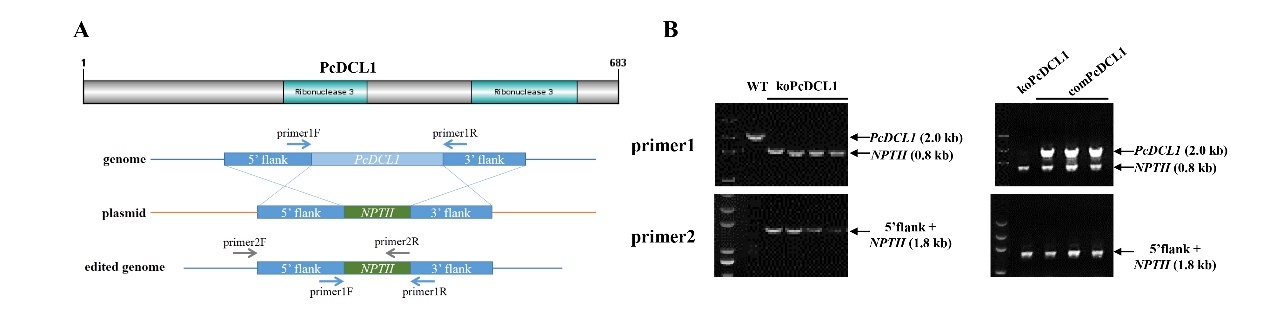


**Fig C. Construction and identification of *PcDCL1* deletion and complemented mutants.** (A) Schematic diagram and gene replacement/complement strategy of PcDCL1. Different domains or gene fragments are denoted by different colors. (B) PCR analysis of the wild-type LT1534, the deletion mutants (koPcDCL1), and complemented isolates (comPcDCL1) using the primers designed as shown in (A).

**
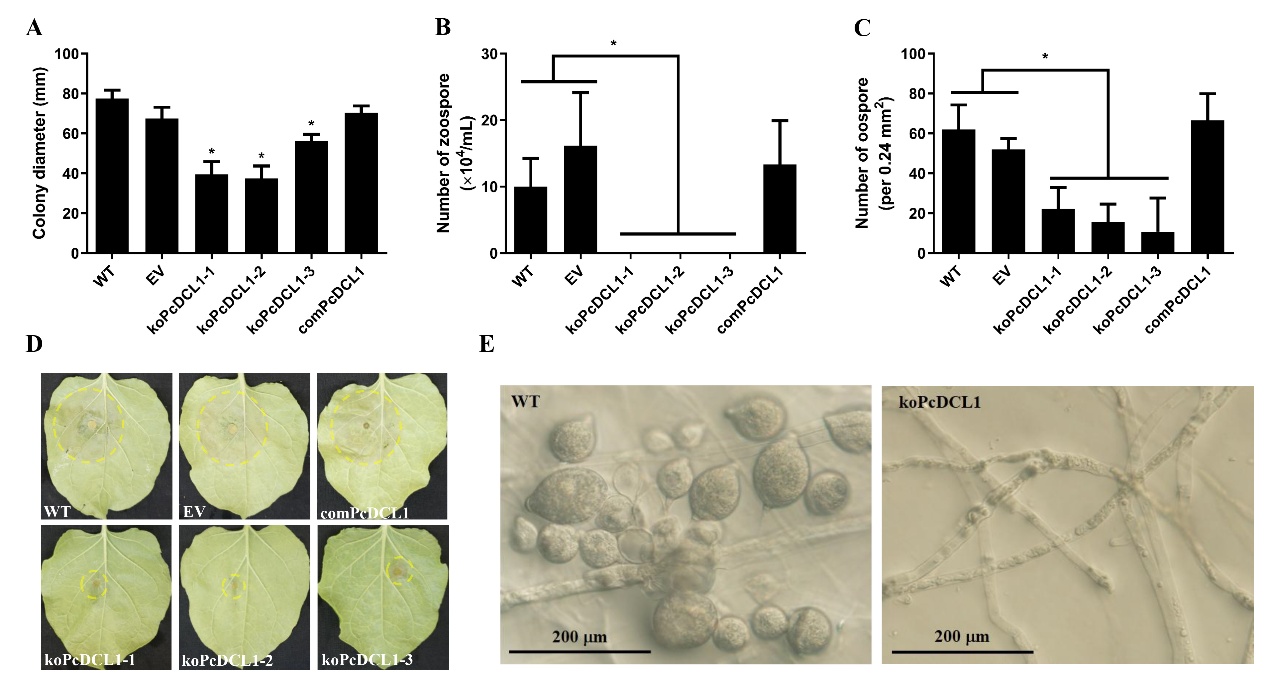
**

**Fig D. PcDCL1 is involved in hyphal growth, zoospores production, oospores production and virulence in *Phytophthora capsici*.** (A-D) The deletion of *PcDCL1* severely impaired hypha growth (A), zoospore production (B), oospore production (C) and virulence (D) in *P. capsici*. Data presented are the mean ± standard deviation from at least five biological replicates. Asterisk represent statistically significant differences according to the one-way ANOVA followed by Fisher’s LSD test (*p* < 0.05). (E) The representative images of sporulated hypha stage of LT1534 (WT) and koPcDCL1. LT1534 was grown for 3.5 days and koPcDCL1 was grown for 7 days on V8 medium under light, respectively. Plenty of sporangia were generated by LT1534 and almost no canonical sporangium could produced by koPcDCL1.


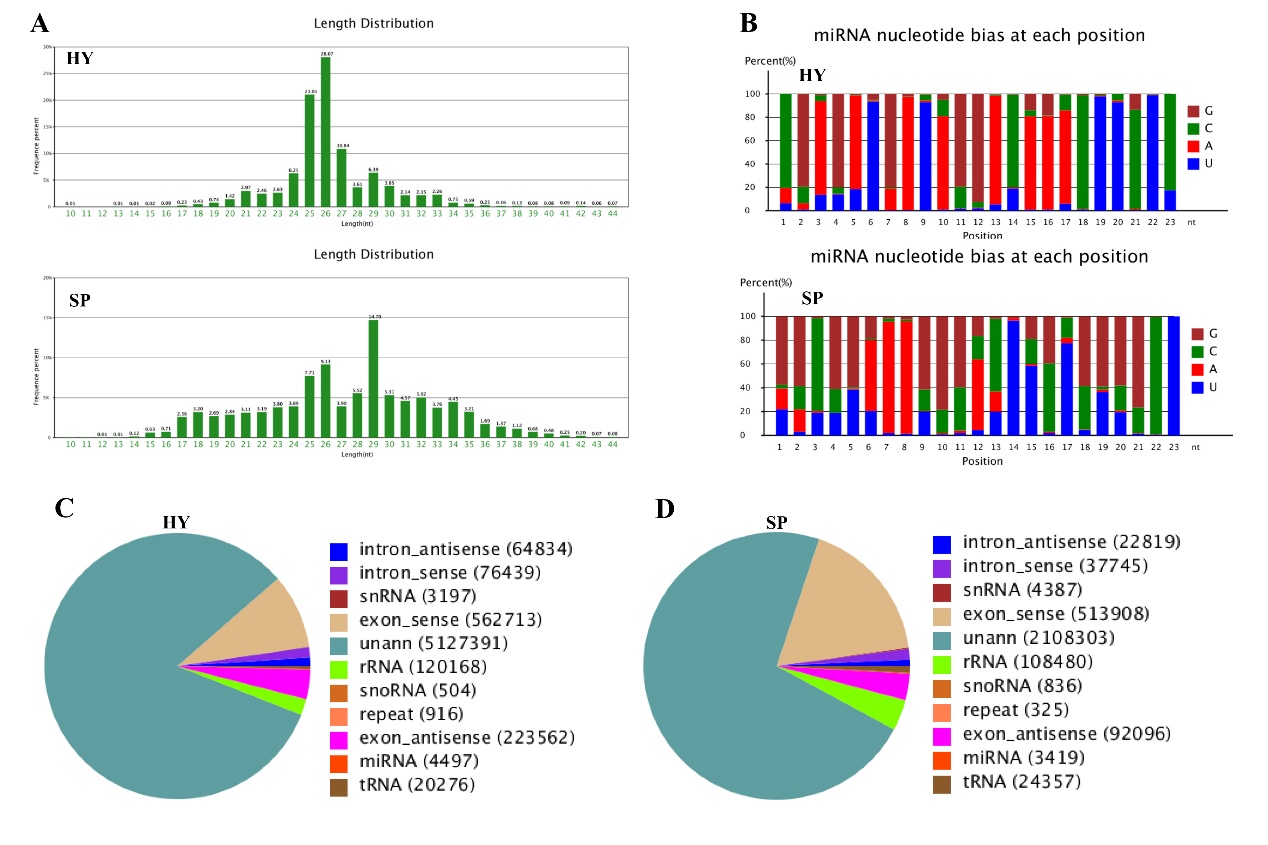


**Fig E. Small RNA-seq assay show distinct small RNA profiles between hypha stage (HY) and sporangium stage (SP) in *Phytophthora capsici*.** (A) The length distribution of small RNAs identified in HY or SP stages. (B) The nucleotide bias at each position in the screened microRNAs identified in HY or SP stages. microRNA is screened by database filtrate and novel microRNA prediction. (C and D) The classification of small RNAs identified in HY (C) or SP (D) according to their genomic distribution. *P. capsici* LT1534 samples of hypha (HY) or sporangia (SP) were prepared and sequenced. HY sample is collected from hypha tissues growing in liquid V8 medium for 48 h. SP sample is collected from sporangia suspension, which prepared by rinsing the sporulated hypha (growing in liquid V8 for 72 h) with pipette for several times and filtered through a 200 μm filter membrane. Sample of SP stage is relatively pure but a handful of small broken hypha are still existed, but > 90% of the total tissue are sporangia according to microscopic observation.


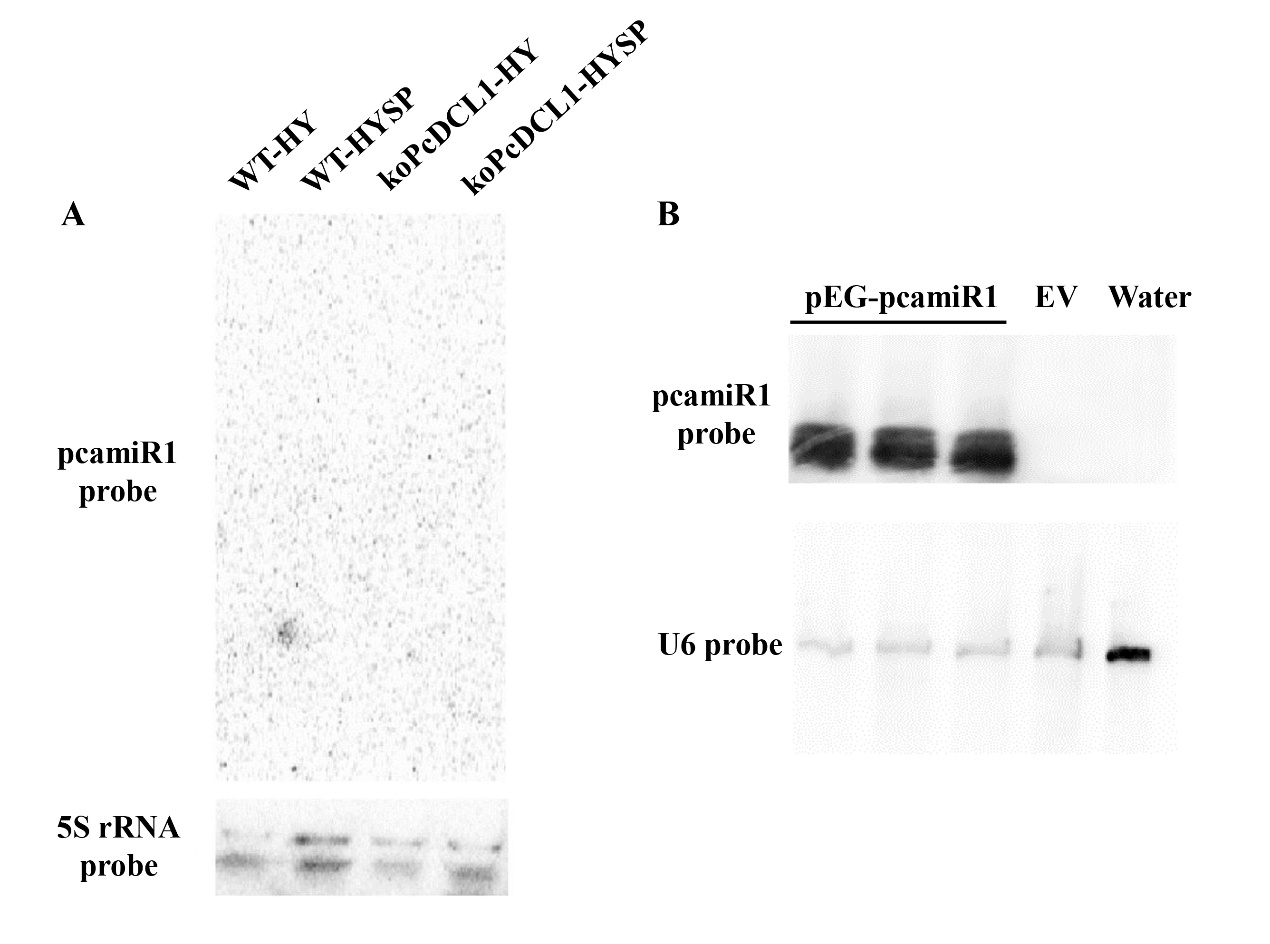


**Fig F. Identification of pcamiR1 in wild-type LT1534 and koPcDCL1 mutants (A) and pcamiR1-expressing *Nicotiana benthamiana* plants (B) by northern blotting.** ^32^P labeled or Digoxin labeled DNA oligo that are reverse-complement with the mature sequence of pcamiR1 are used as probes to detect the expression of pcamiR1 in *Phytophthora capsici* or *N. benthamiana*, respectively. *5S rRNA* gene and *U6* gene are used as loading control in *P. capsici* and *N. benthamiana*, respectively. Their 21-22-nt probes are also reverse-complement to the target genes and labeled with digoxin.


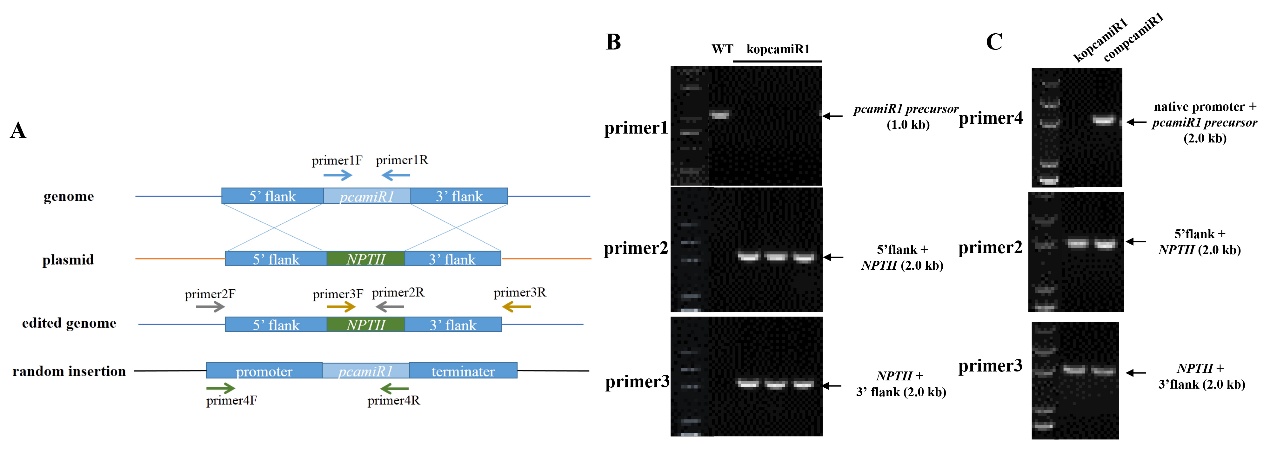


**Fig G. Construction and identification of pcamiR1 deletion and complemented mutants.** (A) Schematic diagram and gene replacement/complement strategy of pcamiR1. Different domains or gene fragments are denoted by different colors. (B and C) PCR analysis of the wild-type LT1534, the deletion mutants (kopcamiR1; B), and complemented isolates (compcamiR1; C) using the primers designed as shown in (A).


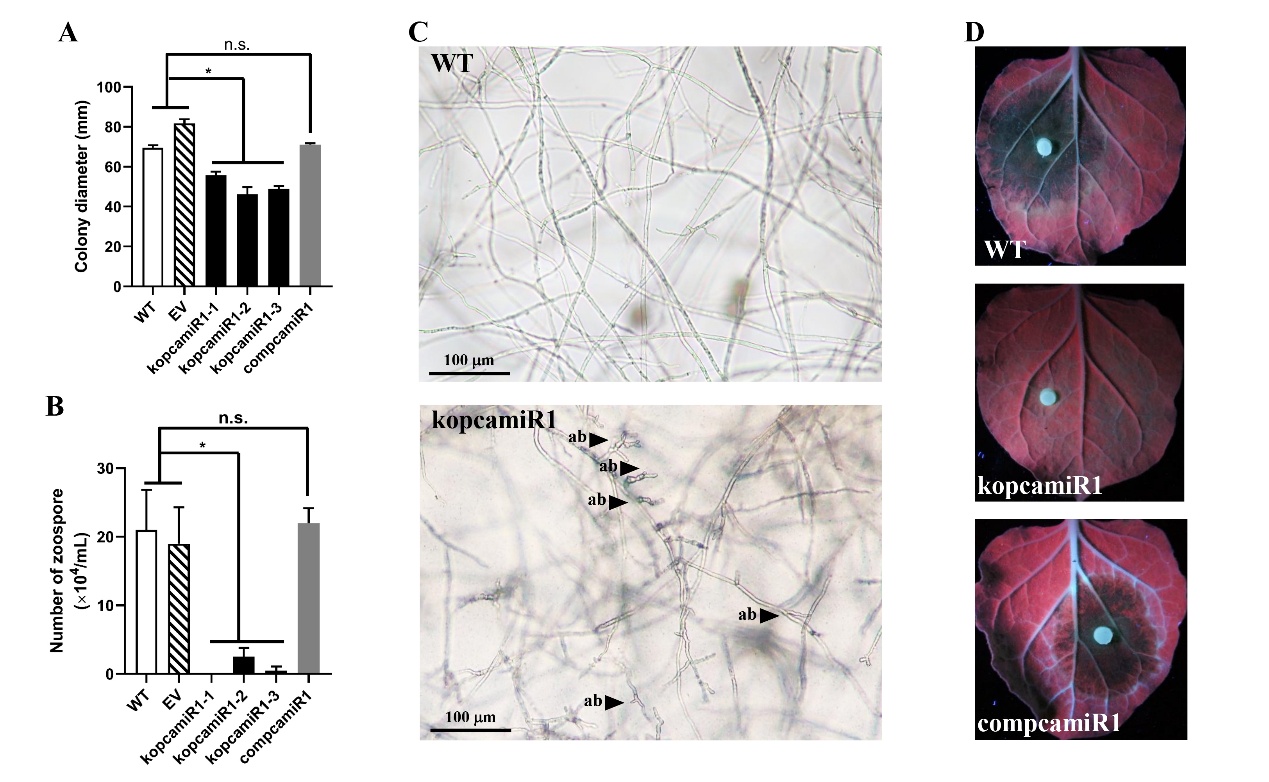


**Fig H.** **pcamiR1 is involved in hyphal growth, zoospores production and virulence in *Phytophthora capsici*.** (A, B and D) The deletion of *PcDCL1* severely impaired hypha growth (A), zoospore production (B), and virulence (D) in *P. capsici*. Data presented are the mean ± standard deviation from at least five biological replicates. Asterisk represent statistically significant differences according to the one-way ANOVA followed by Fisher’s LSD test (*p* < 0.05). (C) The morphology of wild-type LT1534 and koPcDCL1 in hypha stage (HY). To eliminate the difference caused by growth rate, LT1534 was grown for 2 days and koPcDCL1 was grown for 3 days on V8 medium under light, respectively. A plenty of abnormal hyphal branches (ab; indicated by black triangles) that might be sporangiophores or their precursors were observed only in pcamiR1 mutants.


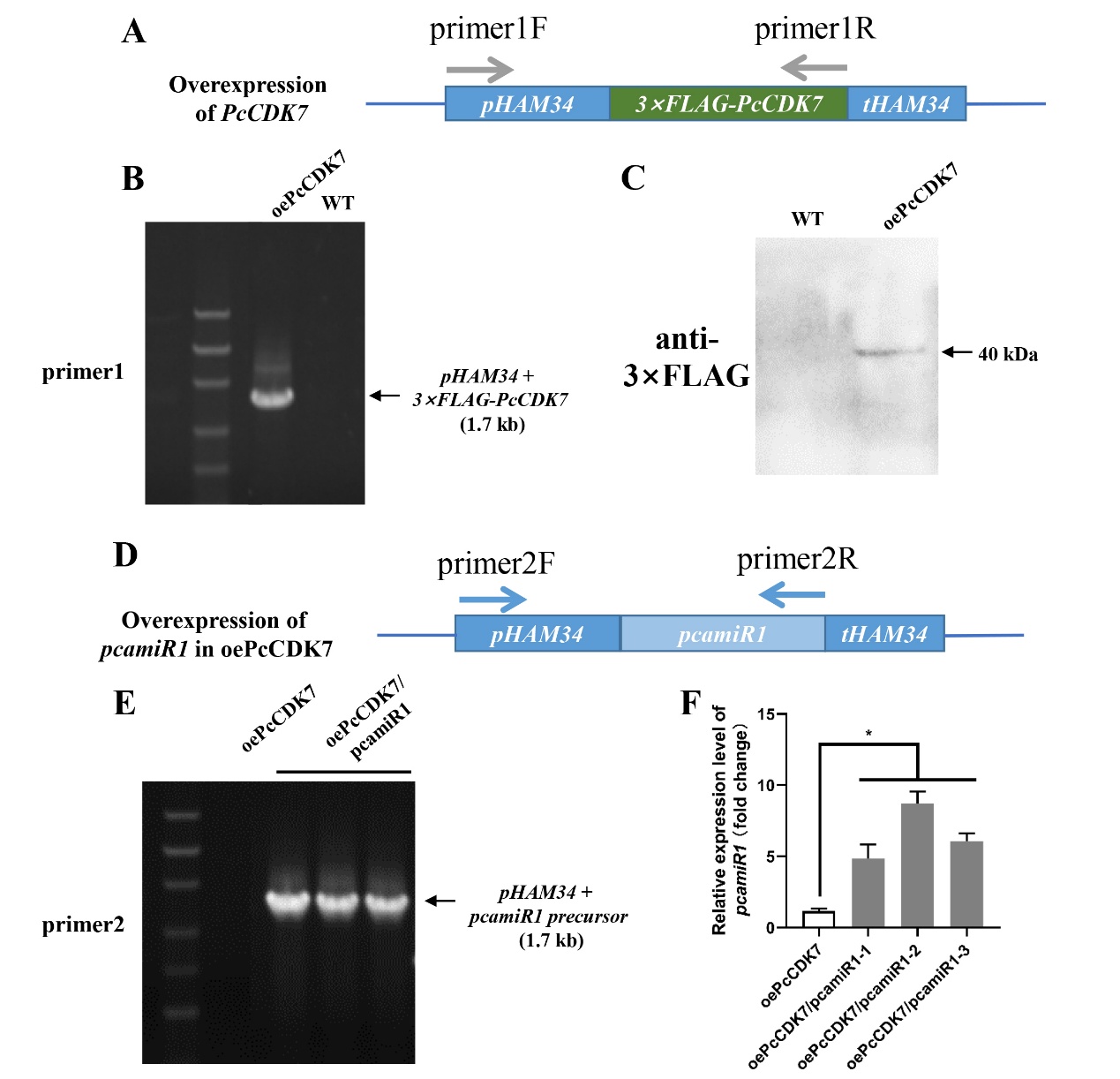


**Fig I. Construction and identification of *PcCDK7* overexpression in LT1534 and pcamiR1 overexpression in oePcCDK7.** (A) Schematic diagram of gene overexpression strategy of *PcCDK7*. (B and C) Verification of *PcCDK7* overexpression by PCR (B) and western blot (C). (D) Schematic diagram of gene overexpression strategy of pcamiR1 in *PcCDK7* overexpression mutant. (E) PCR analysis of the double genes overexpression using the primers designed as shown in (D). (F) Confirmation of the overexpression of pcamiR1 in oePcCDK7 by qRT-PCR. Data presented are the mean ± standard deviation from three biological replicates. Asterisk represent statistically significant differences according to the one-way ANOVA followed by Fisher’s LSD test (*p* < 0.05).


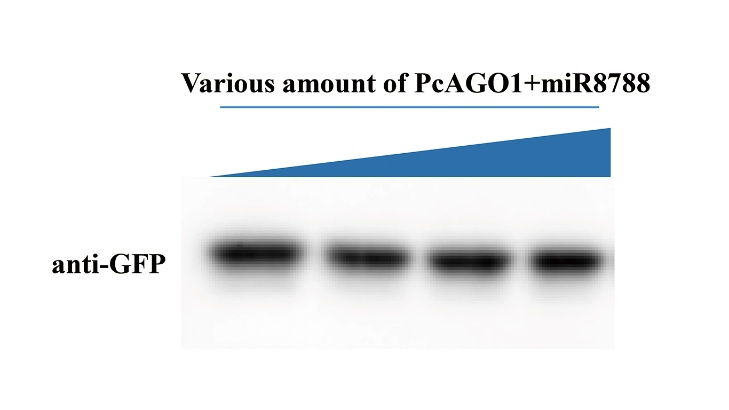


**Fig J. miR8788-AGO1 could not inhibit target gene translation regardless of its concentration.**


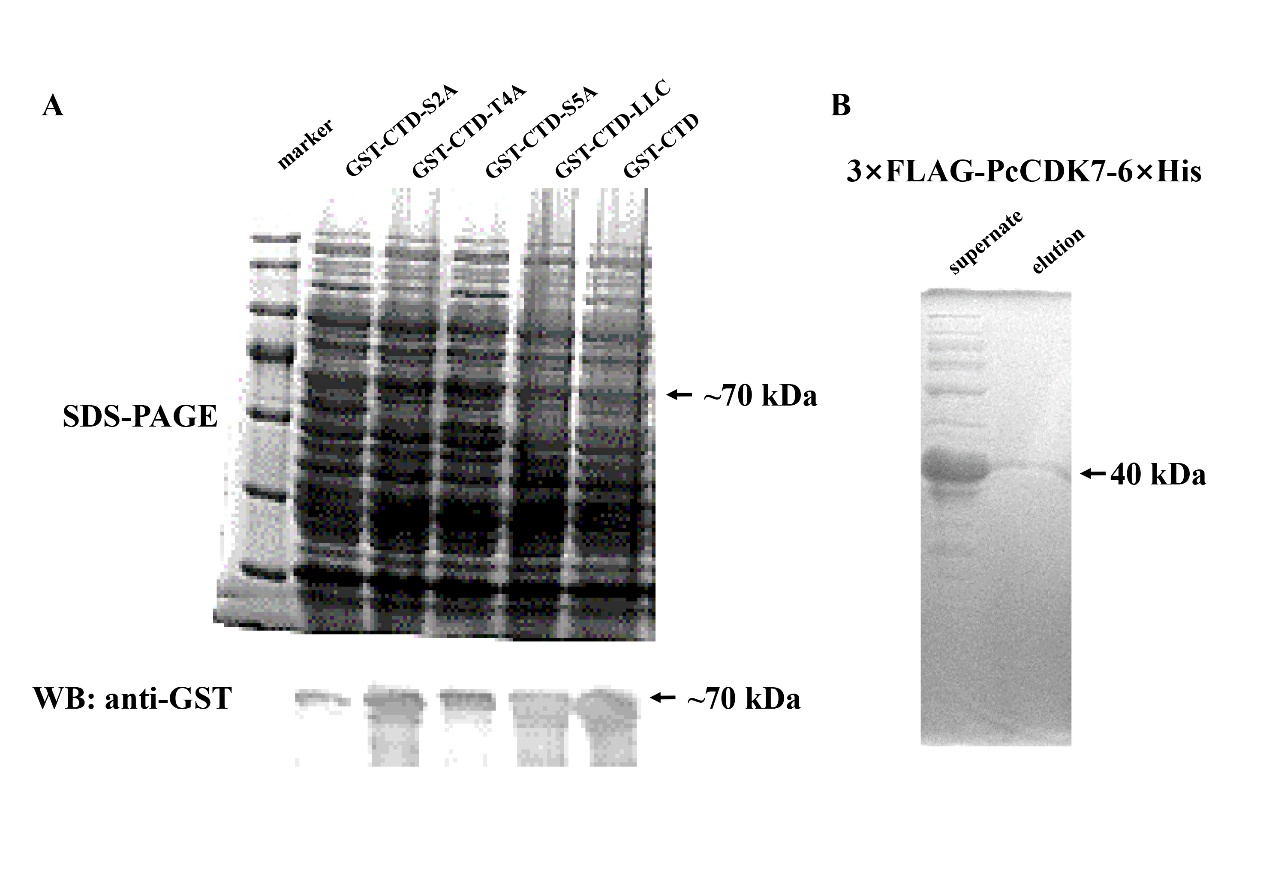


**Fig K. Expression and purification of PcCDK7 and CTD (carboxyl terminal domain of RNPII largest subunit 1) and its mutated proteins.** (A) GST tagged CTD and mutated proteins (S2A/T4A/S5A single amino acid mutated proteins in the heptapeptide and the truncate protein without the last copy of the heptapeptide) were expressed in *Escheria coli* BL21 and detected by SDS-PAGE (upper panel), then they were purified by using GST-sepharose beads and detected by western blot (lower panel). (B) Carboxyl 3×FLAG and amino terminal 6×His fused PcCDK7 was expressed in *E. coli* BL21 and purified by using Ni-NTA affinity chromatography. Coomassie Brilliant Blue G-250 staining showed the protein samples resolved by SDS-PAGE.


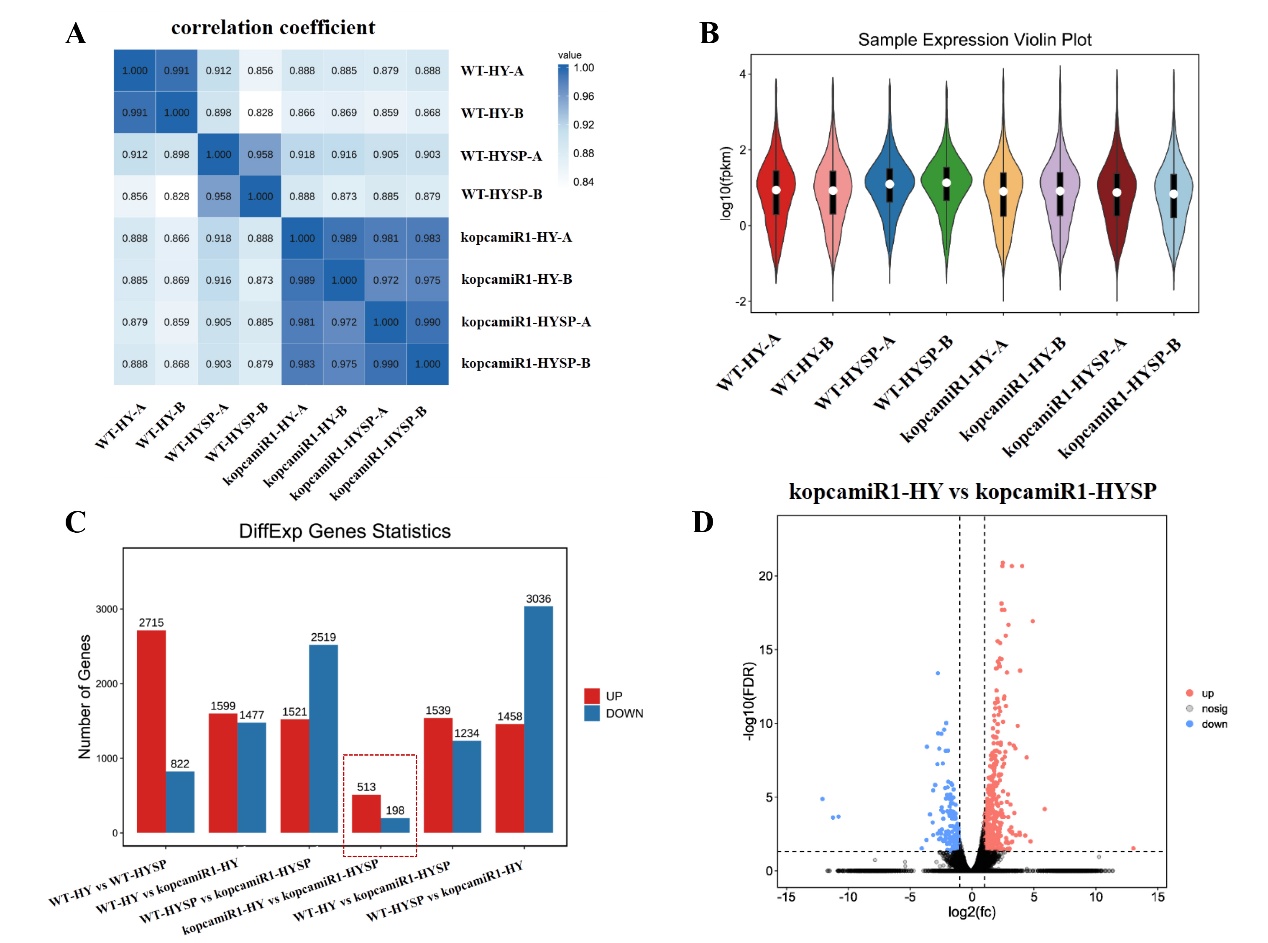


**Fig L. Transcriptomic comparison of wild-type LT1534 and kopcamiR1 in hypha (HY) and sporulated hypha (HYSP) stages.** Three phosphorylation states of RNPII (hypo-phosphorylation: LT1534-HY, moderate-phosphorylation: LT1534-HYSP, hyper-phosphorylation: kopcamiR1-HY and HYSP) were characterized in the two isolates in two stages, their roles in transcriptional regulation were explored by RNA-seq. (A) Correlation heat map analysis of the transcriptomic samples. Pearson correlation coefficient was calculated after pairwise comparison. The stronger correlation showed as darker color. (B) Violin plot analysis of global gene expression levels. The average gene expression level and the level range were compared by the log10(fpkm) of each samples. (C) The statistics of differentially expressed genes (DEGs) in each pairwise comparisons. For each comparison group, genes with FDR < 0.05 and |log2FC| > 1 were selected as DEGs and further been analyzed. In each A vs B groups, up means gene was upregulated in B compared to A, down means gene was downregulated in B compared to A. (D) The volcano map of the DEGs in kopcamiR1-HY vs kopcamiR1-HYSP group. As shown in D (also labeled in C), DEGs in the two stages of kopcamiR1 are both few, and almost all the differentially expressed genes in the group were only slightly changed (1 < |log2FC| < 5). The genes were jointly upregulated or downregulated in both the two biological repeats were analyzed in C and D.


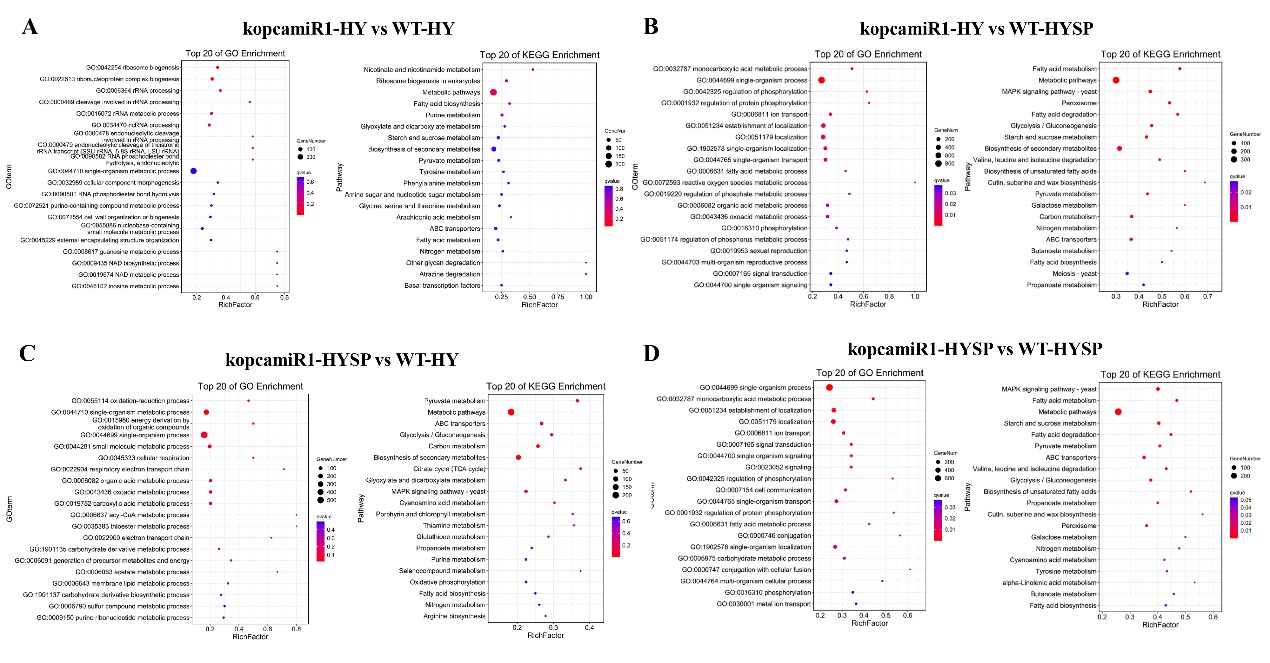


**Fig M. The transcriptome differences happened between wild-type *Phytophthora capsici* and the important regulator pcamiR1 deficient mutant during sporangia development.** Gene ontology (GO) analysis and KEGG (Kyoto Encyclopedia of Genes and Genomes) analysis of the differentially expressed genes (DEGs) in wild-type LT1534 (WT) and kopcamiR1 in hypha (HY) or sporulated hypha (HYSP) stages were performed. Genes with FDR < 0.05 and |log2FC| > 1 were selected as DEGs and further been analyzed in pairwise comparisons. Top20 GO and KEGG terms (with lowest Q values) were shown in kopcamiR1-HY vs WT-HY group (A), kopcamiR1-HY vs WT-HYSP group (B), kopcamiR1-HYSP vs WT-HY group (C), and kopcamiR1-HYSP vs WT-HYSP group (D), which could comprehensively show the transcriptome profile discrepancies and the importance of pcamiR1 in regulating sporangium development. The genes were jointly upregulated or downregulated in both the two biological repeats were analyzed.


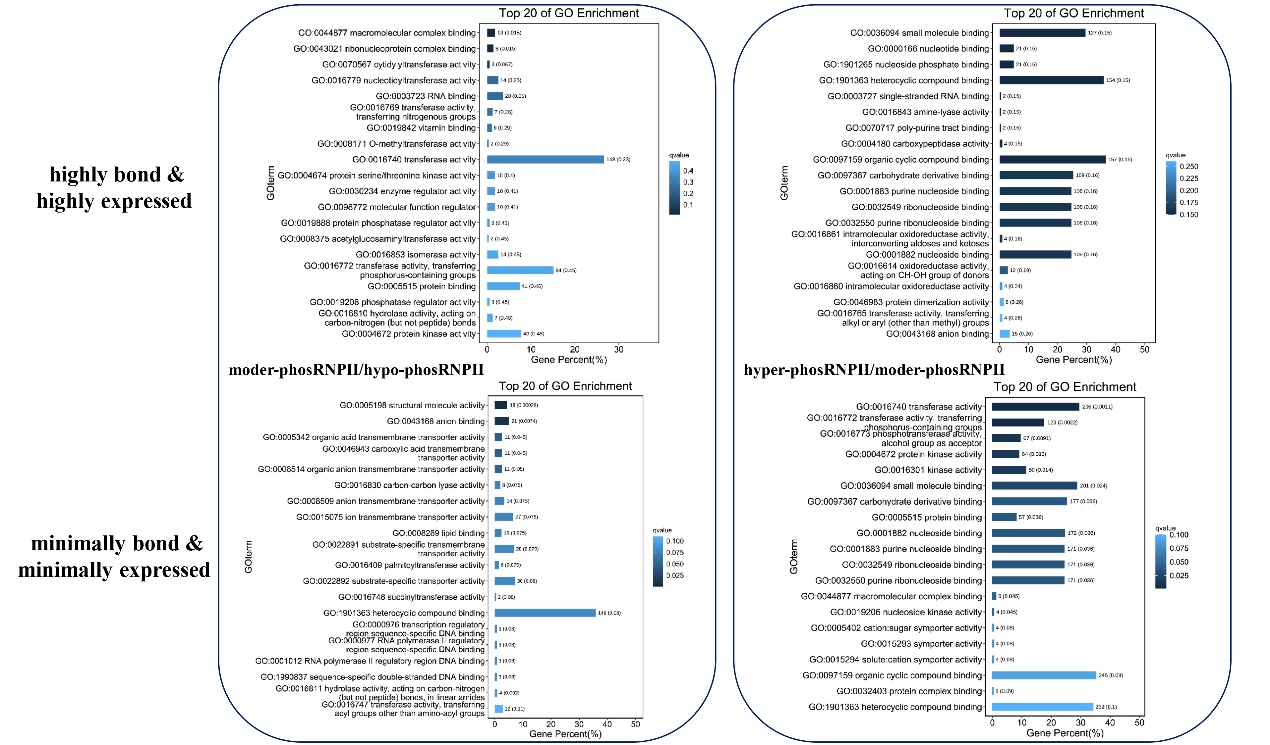


**Fig N. Gene ontology (GO) analysis of the positively related genes both significantly changed in ChIP-seq and RNA-seq.** The genes that enriched in chromatin immunoprecipitation with RNPII and with upregulated gene expression level, or reduced in chromatin immunoprecipitation with RNPII and with down-regulated gene expression level were screened by comparison in two groups and analyzed. The two groups were: (A) moderate-phosphorylated RNPII vs hypo-phosphorylated RNPII and (B) hyper-phosphorylated RNPII vs moderate-phosphorylated RNPII. The top20 GO terms were shown, which could exhibit the pathways most likely involved in normal development of sporangium in *Phytophthora capsici*. The genes were jointly upregulated or downregulated in the two biological repeats of RNA-seq and the peaks called in both the two sequencing data of each sample were analyzed.

**Reference**

1. Bujny MV, Ewels PA, Humphrey S, Attar N, Jepson MA, Cullen PJ. Sorting nexin-1 defines an early phase of Salmonella-containing vacuole-remodeling during Salmonella infection. Journal of cell science. 2008;121(12):2027-36.

2. Herrmann BG, Koschorz B, Wertz K, McLaughlin KJ, Kispert A. A protein kinase encoded by the t complex responder gene causes non-mendelian inheritance. Nature. 1999;402(6758):141-6.

3. Aono S. The Dos family of globin-related sensors using PAS domains to accommodate haem acting as the active site for sensing external signals. Advances in Microbial Physiology. 2013;63:273-327.

4. Calderon-Dominguez M, Gil G, Medina MA, Pandak WM, Rodríguez-Agudo D. The StarD4 subfamily of steroidogenic acute regulatory-related lipid transfer (START) domain proteins: new players in cholesterol metabolism. The international journal of biochemistry & cell biology. 2014;49:64-8.

5. Gatta AT, Wong LH, Sere YY, Calderón-Noreña DM, Cockcroft S, Menon AK, et al. A new family of StART domain proteins at membrane contact sites has a role in ER-PM sterol transport. elife. 2015;4:e07253.

6. Hutten S, Kehlenbach RH. CRM1-mediated nuclear export: to the pore and beyond. Trends in cell biology. 2007;17(4):193-201.

7. Rigden DJ. The histidine phosphatase superfamily: structure and function. Biochemical Journal. 2008;409(2):333-48.

8. Kramer S, Kimblin NC, Carrington M. Genome-wide in silico screen for CCCH-type zinc finger proteins of Trypanosoma brucei, Trypanosoma cruzi and Leishmania major. BMC genomics. 2010;11(1):1-13.

9. Breithaupt C, Strassner J, Breitinger U, Huber R, Macheroux P, Schaller A, et al. X-ray structure of 12-oxophytodienoate reductase 1 provides structural insight into substrate binding and specificity within the family of OYE. Structure. 2001;9(5):419-29.

10. Breithaupt C, Kurzbauer R, Schaller F, Stintzi A, Schaller A, Huber R, et al. Structural basis of substrate specificity of plant 12-oxophytodienoate reductases. Journal of molecular biology. 2009;392(5):1266-77.

11. Permpoonpattana P, Phetcharaburanin J, Mikelsone A, Dembek M, Tan S, Brisson M-C, et al. Functional characterization of Clostridium difficile spore coat proteins. Am Soc Microbiol; 2013. p. 1492-503.

12. Kim H, Hahn M, Grabowski P, McPherson DC, Otte MM, Wang R, et al. The Bacillus subtilis spore coat protein interaction network. Molecular microbiology. 2006;59(2):487-502.

13. Copoiu L, Torres PH, Ascher DB, Blundell TL, Malhotra S. ProCarbDB: a database of carbohydrate-binding proteins. Nucleic acids research. 2020;48(D1):D368-D75.

14. Pao SS, Paulsen IT, Saier Jr MH. Major facilitator superfamily. Microbiology and molecular biology reviews. 1998;62(1):1-34.

15. McKie AT, Barrow D, Latunde-Dada GO, Rolfs A, Sager G, Mudaly E, et al. An iron-regulated ferric reductase associated with the absorption of dietary iron. Science. 2001;291(5509):1755-9.

16. Dancis A, Klausner R, Hinnebusch A, Barriocanal J. Genetic evidence that ferric reductase is required for iron uptake in Saccharomyces cerevisiae. Molecular and Cellular Biology. 1990;10(5):2294-301.

17. Vlanti A, Diallinas G. The Aspergillus nidulans FcyB cytosine‐purine scavenger is highly expressed during germination and in reproductive compartments and is downregulated by endocytosis. Molecular microbiology. 2008;68(4):959-77.

18. Yue C, Cao H, Wang L, Zhou Y, Hao X, Zeng J, et al. Molecular cloning and expression analysis of tea plant aquaporin (AQP) gene family. Plant physiology and biochemistry. 2014;83:65-76.

19. Jack DL, Paulsen IT, Saier MH. The amino acid/polyamine/organocation (APC) superfamily of transporters specific for amino acids, polyamines and organocations. Microbiology. 2000;146(8):1797-814.

20. Zang D, Wang C, Ji X, Wang Y. Tamarix hispida zinc finger protein ThZFP1 participates in salt and osmotic stress tolerance by increasing proline content and SOD and POD activities. Plant Science. 2015;235:111-21.

21. Singhania RR, Patel AK, Sukumaran RK, Larroche C, Pandey A. Role and significance of beta-glucosidases in the hydrolysis of cellulose for bioethanol production. Bioresource technology. 2013;127:500-7.

22. Chou T-F, Cheng J, Tikh IB, Wagner CR. Evidence that human histidine triad nucleotide binding protein 3 (Hint3) is a distinct branch of the histidine triad (HIT) superfamily. Journal of molecular biology. 2007;373(4):978-89.

23. Kamoun S, van West P, de Jong AJ, de Groot KE, Vleeshouwers VG, Govers F. A gene encoding a protein elicitor of Phytophthora infestans is down-regulated during infection of potato. Molecular Plant-Microbe Interactions. 1997;10(1):13-20.

24. Nakayashiki H, Matsuo H, Chuma I, Ikeda K, Betsuyaku S, Kusaba M, et al. Pyret, a Ty3/Gypsy retrotransposon in Magnaporthe grisea contains an extra domain between the nucleocapsid and protease domains. Nucleic acids research. 2001;29(20):4106-13.

25. Telesnitsky A, Goff S. Reverse transcriptase and the generation of retroviral DNA. 2011.

26. Cho S, Park JS, Kang Y-K. Dual functions of histone-lysine N-methyltransferase Setdb1 protein at promyelocytic leukemia-nuclear body (PML-NB): maintaining PML-NB structure and regulating the expression of its associated genes. Journal of Biological Chemistry. 2011;286(47):41115-24.

27. Zhang L, Chen L, Gao C, Chen E, Lightle AR, Foulke L, et al. Loss of histone H3 K79 methyltransferase Dot1l facilitates kidney fibrosis by upregulating endothelin 1 through histone deacetylase 2. Journal of the American Society of Nephrology. 2020;31(2):337-49.

28. Kwon HJ, Tirumalai R, Landy A, Ellenberger T. Flexibility in DNA recombination: structure of the lambda integrase catalytic core. Science. 1997;276(5309):126-31.

29. Mohler PJ, Schott J-J, Gramolini AO, Dilly KW, Guatimosim S, duBell WH, et al. Ankyrin-B mutation causes type 4 long-QT cardiac arrhythmia and sudden cardiac death. Nature. 2003;421(6923):634-9.

30. Koch I, Schwarz H, Beuchle D, Goellner B, Langegger M, Aberle H. Drosophila ankyrin 2 is required for synaptic stability. Neuron. 2008;58(2):210-22.

31. Wu Y, Wan T, Zhou X, Wang B, Yang F, Li N, et al. Hsp70-like Protein 1 fusion protein enhances induction of carcinoembryonic antigen–specific CD8+ CTL response by dendritic cell vaccine. Cancer Research. 2005;65(11):4947-54.

32. Yuvaniyama J, Denu JM, Dixon JE, Saper MA. Crystal structure of the dual specificity protein phosphatase VHR. Science. 1996;272(5266):1328-31.

33. Littlefield O, Nelson H. A new use for the'wing'of the'winged'helix-turn-helix motif in the HSF–DNA cocrystal. Nature structural biology. 1999;6(5):464-70.

34. Aceituno-Valenzuela U, Micol-Ponce R, Ponce MR. Genome-wide analysis of CCHC-type zinc finger (ZCCHC) proteins in yeast, Arabidopsis, and humans. Cellular and Molecular Life Sciences. 2020;77(20):3991-4014.

35. Lowther WT, Matthews BW. Structure and function of the methionine aminopeptidases. Biochimica et Biophysica Acta (BBA)-Protein Structure and Molecular Enzymology. 2000;1477(1-2):157-67.

36. Chang S, McGARY EC, Chang S. Methionine aminopeptidase gene of Escherichia coli is essential for cell growth. Journal of bacteriology. 1989;171(7):4071-2.

37. Chen H-K, Yeh N-H. The nucleolar phosphoprotein P130 is a GTPase/ATPase with intrinsic property to form large complexes triggered by F− and Mg2+. Biochemical and biophysical research communications. 1997;230(2):370-5.

38. Tian Y, Denda-Nagai K, Tsukui T, Ishii-Schrade KB, Okada K, Nishizono Y, et al. Mucin 21 confers resistance to apoptosis in an O-glycosylation-dependent manner. Cell death discovery. 2022;8(1):1-12.

39. Srivastava A, Ambrósio DL, Tasak M, Gosavi U, Günzl A. A distinct complex of PRP19-related and trypanosomatid-specific proteins is required for pre-mRNA splicing in trypanosomes. Nucleic acids research. 2021;49(22):12929-42.

40. Cabreira-Cagliari C, Dias NdC, Bohn B, Fagundes DGdS, Margis-Pinheiro M, Bodanese-Zanettini MH, et al. Revising the PLAC8 gene family: from a central role in differentiation, proliferation, and apoptosis in mammals to a multifunctional role in plants. Genome. 2018;61(12):857-65.

41. Vale RD, Reese TS, Sheetz MP. Identification of a novel force-generating protein, kinesin, involved in microtubule-based motility. Cell. 1985;42(1):39-50.
